# Recommendations for research studies on treatment of idiopathic scoliosis: Consensus 2014 between SOSORT and SRS non–operative management committee

**DOI:** 10.1186/s13013-014-0025-4

**Published:** 2015-03-07

**Authors:** Stefano Negrini, Timothy M Hresko, Joseph P O’Brien, Nigel Price, SOSORT Boards, SRS Non-Operative Committee

**Affiliations:** Clinical and Experimental Sciences Department, University of Brescia, Viale Europa 32, 25123 Brescia, Italy; IRCCS Fondazione Don Gnocchi, Milan, Italy; Department of Orthoapedic Surgery, Harvard Medical School, Boston Children’s Hospital, Boston, USA; National Scoliosis Foundation, Stoughton, MA USA; Children’s Mercy Hospital, University of Missouri Kansas City, Kansas, USA

## Abstract

The two main societies clinically dealing with idiopathic scoliosis are the Scoliosis Research Society (SRS), founded in 1966, and the international Society on Scoliosis Orthopedic and Rehabilitation Treatment (SOSORT), started in 2004. Inside the SRS, the Non-Operative Management Committee (SRS-NOC) has the same clinical interest of SOSORT, that is the Orthopaedic and Rehabilitation (or Non-Operative, or conservative) Management of idiopathic scoliosis patients. The aim of this paper is to present the results of a Consensus among the best experts of non-operative treatment of Idiopathic Scoliosis, as represented by SOSORT and SRS, on the recommendation for research studies on treatment of Idiopathic Scoliosis. The goal of the consensus statement is to establish a framework for research with clearly delineated inclusion criteria, methodologies, and outcome measures so that future meta- analysis or comparative studies could occur. A Delphi method was used to generate a consensus to develop a set of recommendations for clinical studies on treatment of Idiopathic Scoliosis. It included the development of a reference scheme, which was judged during two Delphi Rounds; after this first phase, it was decided to develop the recommendations and 4 other Delphi Rounds followed. The process finished with a Consensus Meeting, that was held during the SOSORT Meeting in Wiesbaden, 8–10 May 2014, moderated by the Presidents of SOSORT (JP O’Brien) and SRS (SD Glassman) and by the Chairs of the involved Committees (SOSORT Consensus Committee: S Negrini; SRS Non-Operative Committee: MT Hresko). The Boards of the SRS and SOSORT formally accepted the final recommendations. The 18 Recommendations focused: Research needs (3), Clinically significant outcomes (4), Radiographic outcomes (3), Other key outcomes (Quality of Life, adherence to treatment) (2), Standardization of methods of non-operative research (6).

**Electronic supplementary material:**

The online version of this article (doi:10.1186/s13013-014-0025-4) contains supplementary material, which is available to authorized users.

## Introduction

The two main societies clinically dealing with idiopathic scoliosis are the Scoliosis Research Society (SRS), founded in 1966, and the international Society on Scoliosis Orthopedic and Rehabilitation Treatment (SOSORT), started in 2004. Inside the SRS, the Non-Operative Management Committee (SRS-NOC) has the same clinical interest of SOSORT, that is the Orthopaedic and Rehabilitation (or Non-Operative, or conservative) Management of idiopathic scoliosis patients.

SOSORT, after verifying the gradual reduction of scientific research in the area of the so-called non-operative treatment [1,2], started producing Consensuses with the aim of reaching a minimum agreement among scientists and clinicians engaged in the field [3-11]. In this respect, in 2011 SOSORT published the Clinical Guidelines that offer a general framework of reference to clinicians treating patients with idiopathic scoliosis [12].

The SRS Non-Operative Management Committee (SRS-NOC) published in 2005 the SRS Criteria for Bracing Studies that constitute the first effort to define precise criteria for conservative treatment research [13]. After this development, a series of papers have been produced respecting these inclusion criteria [14-19]. Also a randomized clinical trial in Bracing Adolescent Scoliosis Trial (BrAIST Study) has been planned accordingly [20], although the inclusion criteria had to be enlarged to aide in recruitment [20,21]. The main strength of the SRS criteria is to focus research on the most important population of patients at risk for progression to a surgical level of treatment. Conversely, the strict inclusion criteria squelched research efforts on non-operative treatment of scoliosis in other populations of patients. Scientific journals abiding to the SRS criteria would not accept manuscript for publication on other populations limiting advancement of knowledge in those areas. In addition, clinicians restricting their treatment regimens to patients within these inclusion criteria run the risk of failing to offer treatment to some patients.

Since the 2nd SOSORT Meeting in Boston 2007, the SRS Presidents have been invited speakers by SOSORT to start collaboration between the two Societies. In the 48th SRS Meeting in Lyon 2013, the SRS-NOC and SOSORT had the first combined educational Meeting on the Non-Operative management of IS. On this occasion it was decided to create a Committee to prepare the first joint SOSORT-SRS Consensus, with the aim to guide future research in the treatment of IS.

The aim of this paper is to present the results of a Consensus among the best experts of non-operative treatment of Idiopathic Scoliosis, as represented by SOSORT and SRS, on the recommendation for research studies on treatment of IS. The goal of the consensus statement is to establish a framework for research with clearly delineated inclusion criteria, methodologies, and outcome measures so that future meta- analysis or comparative studies could occur.

## Methods

### Design

A Delphi method was used to generate a consensus to develop a set of recommendations for clinical studies on treatment of Idiopathic Scoliosis. It included the development of a reference scheme, which was judged during two Delphi Rounds; after this first phase, it was decided to develop the recommendations and 4 other Delphi Rounds followed. The process finished with a Consensus Meeting, that was held during the SOSORT Meeting in Wiesbaden, 8–10 May 2014, moderated by the Presidents of SOSORT (JP O’Brien) and SRS (SD Glassman) and by the Chairs of the involved Committees (SOSORT Consensus Committee: S Negrini; SRS Non-Operative Committee: MT Hresko). The Boards of the SRS and SOSORT formally accepted the final recommendations.

### Participants

The participants were the two main scientific Societies dealing with scoliosis: SOSORT through the Executive Committee and the Advisory Board (SOSORT Boards), its members and the participants at their 2014 Annual Meeting; the SRS through the Non-Operative Management Committee, and the members who participated at the Consensus Meeting during the 2014 SOSORT Annual Meeting in Wiesbaden, 8–10 May 2014.

### The Delphi consensus procedure

#### First reference scheme

The main idea behind the first reference scheme for research studies was to solicit authors to focus on meaningful clinical populations while writing their papers. This did not mean that that they should limit their studies to these groups of patients: while producing data with different groupings, the idea was to require them to report their data according to this specific reference scheme, to facilitate future meta-analysis and pooling of data.

#### Recommendations

After the first two Delphi Rounds, it was clear that it was not possible to reach any Consensus about the reference scheme. Nevertheless, according to the suggestions received, and the previous discussions inside the SOSORT and with the Chair of the SRS Non-Operative Committee, it was possible to develop a series of recommendations that were submitted to the Delphi Consensus procedure.

#### Delphi process

The Consensus procedure followed the Delphi principles [22]. All stages have been be coordinated by the main author (S Negrini), Chair of the SOSORT Consensus Committee, in strict collaboration with a Joint SOSORT-SRS Commission (JSSC) involving another member nominated by the SOSORT Board (JP O’Brien) and two members nominated by the SRS (T Hresko, N Price); in some phases also the SRS President (SD Glassman) has been involved.

The procedure included 8 Rounds. Documents distributed among the participants to the Delphi process were drafted by the first author (SN), and reviewed and approved by the JSSC. Table 1 reports the details of each single Delphi Round (Additional files 1, 2, 3, 4, 5, 6, 7, 8, 9, 10, 11, 12, 13, 14, 15 and 16). SOSORT Boards include the Executive Committee and the Advisory Board.

**Table 1:** **Details of each single Delphi Round performed**

**Delphi Round**	**Methods**	**Participants**	**Material discussed**	**Additional file**
1	Email discussion	SOSORT-SRS Joint Commission	Reference scheme	1
			Consensus Methods ver. 1	2
2	Email questionnaire	SOSORT Boards	Consensus Methods ver. 2	3
		SRS Non-Operative Committee	Discussion ver 1	4
			Questionnaire 1	5
3	Email discussion	SOSORT-SRS Joint Commission	Discussion ver 2	6
			Recommendations ver 1	7
4	Email questionnaire	SOSORT Boards	Consensus Methods ver. 3	8
		SRS Non-Operative Committee	Recommendations ver 2	9
			Discussion ver 3	10
			Questionnaire 2	11
5	SurveyMonkey	SOSORT Boards	Questionnaire 3	12
		SRS Non-Operative Committee		
		SRS Presidential Line		
		SOSORT members		
		SOSORT Meeting participants		
6	Email questionnaire	SOSORT Boards	Questionnaire 4	13
		SRS Non-Operative Committee		
7	Consensus Session	SOSORT Boards	Discussion ver 4	14
		SRS Non-Operative Committee	Questionnaire 5	15
		SRS Presidential Line		
		SOSORT members		
8	Boards approvals	SOSORT Board	Final results	16
		SRS Board		

### Agreement and importance of the recommendations

The SSJC decided to rate the recommendations according to the agreement reached at each stage and the importance defined by the participants at the Delphi procedure.

Definitions of Agreement reached for recommendations are reported in Table 2. The answers to the questions were mutually exclusive (Yes/No): if a recommendation did not reach at least 80% of Agreement it was rejected and not considered any more.

**Table 2:** **Definitions of Agreement reached for recommendations**

**Answers**	**Rating**
100%	A - Complete
95-99.9%	B - High
90-94.9%	C - Good
80-89.9%	D - Weak
Below 80%	Absent

Definitions of the importance of recommendations are reported in Table 3. Importance of the Recommendation was defined using a 5 point Likert scale: 1-Very Low; 2-Low; 3-Medium; 4-High; 5-Very High.

**Table 3:** **Definitions of the importance of recommendations**

**Answers**	**Rating**
4.5-5	1- Very High
3.5-4.4	2- High
2.5-3.4	3- Medium
1.5-2.4	4- Low
1-1.4	5- Very Low

## Results

The number of responders to each Delphi Round is listed in Table 4, with their gender and profession; the rate of responders per group involved is reported in Table 5. All persons who participated at the Consensus and gave consent to be cited are listed in Table 6.

**Table 4:** **Number of responders to each Delphi Round, with their gender and profession**

**Delphi Round**	**Respondents**	**Gender**		**Profession**					
		**Males**	**Females**	**OS**	**PRM**	**PT**	**ORT**	**PhD**	**Others**
1	4	100%	0	50%	25%	0	0	0	25%
2	14	85%	15%	35.5%	28.5%	14%	7%	7%	7%
3	4	100%	0	50%	25%	0	0	0	25%
4	16	94%	6%	37.5%	31%	12.5%	6%	6%	6%
5	146	55.5%	45.5%	17.5%	17%	36%	15%	11%	19.5%
6	7	100%	0	42.5%	42.5%	0	0	15%	0
7	47	65.5%	33.5%	23.5%	17%	27.6%	21.3%	10.5%	15%

OS: Orthopedic Surgeons; PRM: Physical and Rehabilitation Medicine Specialists, PT: Physiotherapists; ORT: Orthotists.

**Table 5:** **Rate of responders per group involved**

**Delphi round**	**Group participants**	**Number**	**Rate of responders**
1	SOSORT-SRS Joint Commission	4	100%
2	SOSORT Boards	13	100%
	SRS Non-Operative Committee	13	47%
3	SOSORT-SRS Joint Commission	4	100%
4	SOSORT Boards	13	100%
	SRS Non-Operative Committee	13	54%
5	SOSORT Boards	13	100%
	SRS Non-Operative Committee	13	100%
	SRS Presidential Line	4	50%
	SOSORT members	150	56%
	SOSORT Meeting participants	180	46%
6	SOSORT Boards	13	46%
	SRS Non-Operative Committee	13	31%
7	SOSORT Boards	13	100%
	SRS Non-Operative Committee	13	46%
	SRS Presidential Line	4	25%
	SOSORT members	180	33%

**Table 6:** **Professionals who participated at the Consensus and gave consent to be cited**

**N**	**Family name**	**Name**	**N**	**Family name**	**Name**	**N**	**Family name**	**Name**
**1**	Abraham	Roby	**50**	Flach	Sabine	**99**	Orban	Judit
**2**	Amorim	Alessandra	**51**	Glassman	Steven	**100**	Orthwein	Patricia
**3**	Apelyan	Mari	**52**	Glinkowski	Wojciech	**101**	Papadopoulos	Dimitris
**4**	Auler	Silke	**53**	Grandinot	Patricia	**102**	Papoulias	Lampros
**5**	Aulisa	Lorenzo	**54**	Grivas	Theodoros	**103**	Parent	Eric
**6**	Aulisa	Angelo Gabriele	**55**	Güttinger	Kathrin	**104**	Parzini	Silvana
**7**	Auner-Gröbl	Petra	**56**	He	Xiaohua (Shawn)	**105**	Pizzetti	Paolo
**8**	Bernard	Jean-Claude	**57**	Hennes	Axel	**106**	Price	Nigel
**9**	Berto	Sofia	**58**	Henning	Susan	**107**	Rexing	Michael
**10**	Bettany-Saltikov	Josette	**59**	Hewitt	Steve	**108**	Rivett	Louann
**11**	Betts	Tony	**60**	Hresko	Timothy	**109**	Roberts	Peter
**12**	Betz	Joseph	**61**	Ibarrondo	Irantzu	**110**	Roig Oliver	Maria Magdalena
**13**	Białek	Marianna	**62**	Iljazi	Harun	**111**	Roller	Matthias
**14**	Bissolotti	Luciano	**63**	Ishihara	Chiiko	**112**	Romano	Michele
**15**	Black	Jason	**64**	Kaefer	Sandra	**113**	Rosellini	Guerrino
**16**	Boltezar	Edita	**65**	Karavidas	Nikos	**114**	Sanchez	Judith
**17**	Boogaart	Mark	**66**	Kerstholt	Janine	**115**	Satyawati	Rwahita
**18**	Bradley	Michael	**67**	Kim	Donghyun	**116**	Schrander	Dirk
**19**	Brox	Jens Ivar	**68**	Kinel	Edyta	**117**	Shackerley-Bennett	Lisa
**20**	Chan	Wing Yan	**69**	Kluszczyński	Marc	**118**	Sieteski	Wojciech
**21**	Chou	Chungwai	**70**	Knott	Patrick	**119**	Silvane	Alina
**22**	Christine	Chenot	**71**	Korbel	Krzysztof	**120**	Simony	Ane
**23**	Claudepierre	Marie-Rose	**72**	Kotwicki	Tomasz	**121**	Speers	David
**24**	Cohen	Larry	**73**	Kozinoga	Mateusz	**122**	Stępień	Agnieszka
**25**	Colomer	Marc	**74**	Landauer	Franz	**123**	Stikeleather	Luke
**26**	Czaprowski	Dariusz	**75**	Laura	Laura Djuriantina	**124**	Stoliński	Łukasz
**27**	D'agata	Elisabetta	**76**	Lebel	Andrea	**125**	Swaminathan	Narasimman
**28**	Dairiany	Tetty Murniaty	**77**	Lind	Tiina	**126**	Talwalkar	Vishwas
**29**	De Lucia	Taissa	**78**	Luchsinger-Lang	Cornelia	**127**	Tassone	Channing
**30**	De Maldè	Daniele	**79**	Lusini	Monia	**128**	Tello	Carlos
**31**	De Mauroy	Jean Claude	**80**	Marcotte	Louise	**129**	Tomasz	Karski
**32**	De Ru	Esther	**81**	Marti	Cindy	**130**	Torres	Beatriz
**33**	De Seze	Mathieu	**82**	Maruyama	Toru	**131**	Tunggawidjaja	Armyn Trimulia Atmadja
**34**	Deceuninck	Julie	**83**	Matthews	Martin	**132**	Ugras	Akin
**35**	Diarbakerli	Elias	**84**	Maude	Erika	**133**	Urban	Bernd
**36**	Diers	Helmut	**85**	Mayr	Maria	**134**	Valer	Beatriz
**37**	Dolan	Lori	**86**	Mentges	Patricia	**135**	Van De Braak	Jan
**38**	Donzelli	Sabrina	**87**	Minnella	Salvatore	**136**	Van Dijk-De Jonge	Marjan
**39**	Doucet	Chantal	**88**	Mols	Francois	**137**	Van Loon	Piet
**40**	Dr Fodor	Janosné	**89**	Monken	Mônica	**138**	Verska	Joseph
**41**	Drake	Shawn	**90**	Monroe	Marcia	**139**	Voets	Helma
**42**	Drobyshevskij	Valerij	**91**	Muccio	Marissa	**140**	Wajchenberg	Marcelo
**43**	Durmala	Jacek	**92**	Negrini	Alessandra	**141**	Wong	M. S.
**44**	Eisenberger	Ossi	**93**	Negrini	Stefano	**142**	Wood	Grant
**45**	Ericson	Sara Rebecca	**94**	Neuhaus Sulam	Lior	**143**	Wynne	James
**46**	Esoinoza	Pamela	**95**	Neuhous	Tamar	**144**	Yilmaz	Hurriyet
**47**	Espinoza	Pamela	**96**	Ng	Shu Yan	**145**	Young	Merlin
**48**	Etemadifar	Mohammadreza	**97**	Novotná	Jitka	**146**	Zaharieva	Darina
**49**	Fabris Monterumici	Daniele	**98**	O'Brien	Joseph	**147**	Zaina	Fabio

Details on the results on each single Round can be found in Attachment 16.

### Recommendations for research studies on treatment of idiopathic scoliosis

#### Research needs

1. We recommend ongoing high quality research and development focused on innovative non operative treatments for scoliosis and related spinal deformities (B2)
2. We recommend that indications and contraindications for non-operative approaches are continuously researched by high quality studies (B2)
3. We recommend that risks and benefits of non-operative treatments be continuously researched by high quality studies (B2)

#### Clinically significant outcomes

4. We recommend that prognostic factors for consequences of the deformity in adulthood on primary patient-centred outcomes (such as aesthetics, deformity progression, disability, pain and quality of life) are continuously researched and better defined by high quality studies (A2)
5. We recommend to systematically report in clinical studies the primary patient-centred (such as aesthetics, disability, pain and quality of life), and the secondary predictive (such as clinical, radiological and topographic data) outcomes of treatment approaches (B2)
6. We recommend that non-operative clinics should focus primarily on clinical outcomes relevant to patients (such as aesthetics, disability, pain and quality of life), and secondarily on predictive outcomes (such as radiographic and topographic data). Clinical, radiological and topographic parameters must be all taken into account for clinical decisions (D2)
7. We recommend to report research results in the clinically significant terms of number of patients at start and end of treatment exceeding 10°, 30° and 50° Cobb: epidemiology recognises these as risk thresholds for possible health consequences in adulthood like back pain and curve progression [3,12,20,21,23-27]. In everyday clinics, the importance of these thresholds should be defined case by case in front of single patients according to many parameters other than Cobb degrees (C2)

#### Radiographic outcomes

8. We recommend that radiographic research outcomes are presented in terms of number of patients improved (6° or more), unchanged (+/−5°) and progressed (6° or more) (C2)
9. We recommend the adoption of the “Risser+” staging. This is the result of the confluence between the original US Risser staging, and the so-called European version of Risser staging as modified by Stagnara [28-30]. Fusion of the tri-radiate cartilage has also been added, as it has been shown to be an important and prognostic subdivision of Risser staging 0. (D2) (***NOTE:****The SRS and SOSORT propose this Consensus Recommendation for further studies of repeatability of the Risser + test proposal before certifying its validity. The main authors are engaged to perform this study. As soon as this study will be performed, the SRS and SOSORT will check it for final approval of the Recommendation*)

10. We recommend that radiographic research outcomes are presented also split in tables according to Cobb degrees at start of treatment (group of 5° Cobb) and bone age (Risser + staging), like the following one (D2):

#### Other key outcomes (Quality of Life, adherence to treatment)

11. We recommend that standardised and validated questionnaires are used to report Quality of Life results (B2)
12. We recommend in clinical research to include data on adherence to treatment: statistical analysis should include these data. Prospective bracing studies must use objective means to monitor adherence. Exercises studies must report data on adherence to number and length of assisted sessions, and home-exercise (B2)

#### Standardization of methods of non-operative research

13. In the introduction of a new non-operative treatment for patients during growth, for the radiographic outcome we recommend that the following research steps are followed: (B2)

14. We recommend in research on non-operative treatment this table, from the Oxford Centre for Evidence-Based Medicine 2011 Levels of Evidence [31] (B2)

15. In the introduction of a new brace, we recommend to focus research on the following SRS inclusion criteria [13]: above 10 years of age, Risser 0–2, curves 25-40° Cobb. (D2)
16. In presenting research results on bracing, we recommend to answer to the questionnaire in Appendix of the SOSORT Guidelines for Management of braced patients [8] to understand how team managed patients. (B3)
17. In presenting results on bracing, we recommend to specify results according to the dosage of bracing in terms of impact on patients’ social life. (B2)

18. At this stage of research on non-operative approaches during growth other than bracing, we strongly recommend to present radiographic results (C2)

## Discussion

In this section we summarize the recommendations in the light of the discussion amongst the participants to the Delphi procedure and the Consensus session.

### Research needs

The first three recommendations have been grouped under the title “research needs”, since they all focus on what and how research should be performed in the near futur.. After a long period in which research on conservative treatment of IS continuously decreased [1], the situation changed in the last 10 years [2]: SOSORT was founded in 2004, and can be a cause or an effect of this change. Since the Cochrane reviews that painted the situation only a few years ago, (2009 for bracing [32,33], 2011 for exercising [34,35]), RCTs published in the literature showed the effectiveness of bracing [21,36] and also of scoliosis specific exercises [37,38]. Nevertheless, there is a strong need to continue this research, and these recommendations focus specifically on this point: they stress the need for innovations (new non-operative treatments), for searching the correct indications and contraindications. These three recommendations also stress the need for high quality studies, and not simply studies with low level of evidence.

1. We recommend ongoing high quality research and development focused on innovative non operative treatments for scoliosis and related spinal deformities (B2)
2. We recommend that indications and contraindications for non-operative approaches are continuously researched by high quality studies (B2)
3. We recommend that risks and benefits of non-operative treatments be continuously researched by high quality studies (B2)

### Clinically significant outcomes

The Cochrane Institute and modern epidemiology stress the need to focus on primary outcomes, the patient centered results, those that really change the life of patients [32,34]. In this perspective, secondary or surrogate outcomes are the biological parameters that predict the primary outcome, but are not directly and immediately connected to the life of patients. In scoliosis research, examples of primary outcomes are Quality of Life, back pain, disability, pulmonary disorders; and progression to surgical treatment. Examples of secondary outcomes are radiographic angles like the Cobb degrees or pelvis parameter, or surface measurements like the Angle of Trunk Rotation (ATR).

This set of recommendations should focus the attention of researchers on primary outcomes, since generally speaking most of the published research is focused on Cobb degrees and other secondary outcomes [3,12]. Nevertheless, since scoliosis treatment during growth is mainly prevention of primary outcomes that will happen in adulthood due to the deformity gradually developed before bone maturity, it must be recognised that secondary outcomes are crucial. In this perspective, it is also recommended to focus research on the real prognostic value of these secondary outcomes for consequences in adulthood in terms of primary outcomes.

4. We recommend that prognostic factors for consequences of the deformity in adulthood on primary patient-centred outcomes (such as aesthetics, deformity progression, disability, pain and quality of life) are continuously researched and better defined by high quality studies (A2)
5. We recommend to systematically report in clinical studies the primary patient-centred (such as aesthetics, disability, pain and quality of life), and the secondary predictive (such as clinical, radiological and topographic data) outcomes of non-operative approaches (B2)
6. We recommend that non-operative clinics should focus primarily on clinical outcomes relevant to patients (such as aesthetics, disability, pain and quality of life), and secondarily on predictive outcomes (such as radiographic and topographic data). Clinical, radiological and topographic parameters must be all taken into account for clinical decisions (D2)
7. We recommend to report research results in the clinically significant terms of number of patients at start and end of treatment exceeding 10°, 30° and 50° Cobb Angle as epidemiology recognises these as risk thresholds for possible health consequences in adulthood like back pain and curve progression [3,12,20,21,23-27]. In everyday clinics, the importance of these thresholds should be defined case by case based on individual patients according to many parameters other than Cobb degrees (C2)

The three thresholds reported have been quite discussed, specifically the 30° Cobb limit. In fact, concerns have been raised about focusing clinicians on this threshold, beyond the classical 50° used by surgeons [20,21,39]. Nevertheless, it must be recognised that the 30° limit has been reported as the boundary before which rarely there are health consequences in adulthood, like back pain or deformity progression. This makes this threshold particularly important for non-operative treatment that should aim at possibly maintaining patients below the limit significant for health.

Another concern about these thresholds was the fear of focusing clinicians mainly on Cobb degrees rather than on other parameters. Each patient is an individual, clinical decision must be made on a case by case basis for each patient.

### Radiographic outcomes

8. We recommend that radiographic research outcomes are presented in terms of number of patients improved (6° or more), unchanged (+/−5°) and progressed (6° or more) (C2)

For some years, it has been emphasized within the SOSORT community that there is the possibility to improve patients with conservative treatment [8,12,40]. For this reason, the classical SRS outcome criteria [13] were perceived as inadequate since they did not allow a description of cases where improvement was achieved. Therefore, while maintaining the classical 5° Cobb threshold to describe a clinically meaningful variation [41], it was decided to add a descriptor of improvement to stability and progression of deformity.

9. We recommend the adoption of the “Risser+” staging. This is the result of the confluence between the original US Risser staging, and the so-called European version of Risser staging as modified by Stagnara [28-30]. It has been added also the tri-radiate cartilage fusion, that has been shown to be an important and prognostic subdivision of Risser staging 0. (D2) (*NOTE: The SRS and SOSORT propose this Recommendation come out as a result of the Consensus for further studies of repeatability of the Risser + test proposal before certifying its validity. The main authors are engaged to perform this study. As soon as this study will be performed, the SRS and SOSORT will check it for final approval of the Recommendation*)

The Risser + staging was an idea that came out during discussion when the importance of other radiographic signs like the tri-radiate cartilage was considered [30,42,43]. Many researchers in the SOSORT community use the so-called European Risser sign [28-30] that is born from a reported by Stagnara, a variation of the original Risser sign (here called US Risser sign). The consequence is that in many studies there is no clear definition of which Risser sign is considered. Unifying all these data coming from the pelvis evaluation was felt to be the first step toward a solution of these discrepancies. Obviously, the already well reported limited value of the Risser sign in describing bone growth and maturity [44-46] is recognised. But the reality remains that most of the studies, even the best ones [13], continue to use this sign since it is readily available in the same x-ray of the spine, without added exposure. The Risser + is simply defined as the convergence of the US and European Risser, adding the tri-radiate cartilage fusion: future studies should focus on this Risser + sign to check its repeatability and validity.

10. We recommend that radiographic research outcomes are presented also split in tables according to Cobb degrees at start of treatment (group of 5° Cobb) and bone age (Risser + staging), like the following one (D2):

The evolution of knowledge in Medicine relies mainly on the research developments carried out by single groups, but the introduction of new statistical techniques allow today to perform so-called meta-studies: specifically, meta-analysis, permit to reach higher level of evidence joining little studies in bigger ones. In this evolution, respecting strict definitions and inclusion criteria, internationally acceptable, is very important to allow proper meta-studies to be performed. This is the reason for the reference scheme reported in the recommendation and proposed to all researchers.

### Other key outcomes (Quality of Life, adherence to treatment)

11. We recommend that standardised and validated questionnaires are used to report Quality of Life results (B2)

The literature on Quality of Life during scoliosis treatments is continuously increasing, as well as the questionnaires to measure it. The gold standard in surgical approaches is the SRS-22 [47,48], although there are doubts of the usefulness in non-operative care [49]. Other questionnaires have been developed and maybe useful in this specific setting if and when validated [50-53].

12. We recommend in clinical research to include data on adherence to treatment: statistical analysis should include these data. Prospective bracing studies must use objective means to monitor adherence. Exercises studies must report data on adherence to number and length of assisted sessions, and home-exercise (B2)

Adherence to treatment should be distinguished from compliance [54]:”Adherence” is the result of the active choice of the patient to comply with the prescribed treatment which is necessary for brace treatment, while “compliance” is a passive behaviour. In non-operative treatment there is the need to look at adherence, since deciding to use a brace and or to perform exercises regularly is a choice to be made every day by the patient. Adherence also underlines the need (stressed in the literature) to help the patient in active participation in the treatment. In clinical research, adherence must be strictly monitored to better describe the treatment performed. A good adherence is not only a matter of feasibility of the non-operative treatment, but it is also a quality check of the entire approach. While for bracing there are now some monitors for adherence [55-60], in case of exercises, diaries are the only means at this point in time.

### Standardization of methods of non-operative research

13. In the introduction of a new non-operative treatment for patients during growth, for the radiographic outcome we recommend that the following research steps are followed: (B2)

One of the problems with assessing the effectiveness of non-operative treatment is the long duration in time in treatment. If the prevailing idea is to publish only final results, then research can be only rare and very sparse, thereby reducing the possibility of knowledge and improvement of treatments. On the other hand, it is not possible to consider as really relevant any research made only on the immediate in-brace results (even if in-brace results can have some predictive validity, still to be well explored) [61-63]. Even less reliable are very short term results immediately after a session, or a period of exercises, or other non-operative treatments (like manual treatments), that can change posture for a while, but without possibility to maintain this change in time like bracing [64,65]: in these cases what is researched is a training [66], and only stable results at least in the short term (12 months) can have some reliability. For this reason, knowing the timing of the results obtained is of high relevance, and very short term results are considered only for bracing.

14. We recommend in research on non-operative treatment this table, from the Oxford Centre for Evidence-Based Medicine 2011 Levels of Evidence [31] (B2)

Level of Evidence is usually required by many spine journals: it was decided to adopt the table most used in the scientific literature.

15. In the introduction of a new brace, we recommend to focus research on the following SRS inclusion criteria [13]: above 10 years of age, Risser 0–2, curves 25-40° Cobb. (D2)

During the Consensus it was decided to exclude the 1-year post-menarche limit for the inclusion criteria for bracing studies, since it was found poorly reliable by some studies presented during SOSORT Meetings [67].

16. In presenting research results on bracing, we recommend to answer to the questionnaire in Appendix of the SOSORT Guidelines for Management of braced patients [14] to understand how team managed patients. (B3)

Adherence to treatment is recognised for a long time as a key factor of bracing: nevertheless, results published using sensors are really different among the research groups [55-60]. It has been shown that the management of patients is crucial for adherence to treatment [8,68]. While searching for Consensus on bracing, SOSORT was not able to find one, neither on the type of brace, nor on the brace’s biomechanical actions [5], although it was possible to agree on the management of patients [8]. The SOSORT criteria for Management of braced patients offer an understanding of how patients were managed, and in the Appendix a questionnaire is given, whose usage would allow researchers to better paint their clinical approach, and readers to really understand the results obtained.

17. In presenting results on bracing, we recommend to specify results according to the dosage of bracing in terms of impact on patients’ social life. (B2)

This was one of the most difficult recommendations, since all clinicians use their own definitions, with different number of hours and dosages. After long discussion, it was decided to maintain this recommendation to reach some kind of agreement as a baseline to move on. This scheme was found as the most reasonable (and voted) since it is quite coherent with real-life habits of patients and not too complicated. Such a scheme is offered to the research community for further understanding.

18. At this stage of research on non-operative approaches during growth other than bracing, we strongly recommend to present radiographic results (C2)

While non-operative treatments can seek results other than the deformity (such as back pain reduction, pulmonary function improvement, quality of life increase and so on) it has been decided after long discussion to give this recommendation, since we cannot ignore the deformity that could progress while treating other key health problems. This eventual progression (or contrarily, improvement) should never be ignored, and always reported, specifically for treatments whose results on the deformity are not yet known in the literature.

## Additional files

Additional file 1:
**First reference scheme.**
Additional file 2:
**Consensus Methods version 1.**
Additional file 3:
**Consensus Methods version 2.**
Additional file 4:
**Discussions version 1.**
Additional file 5:
**Questionnaire 1.**
Additional file 6:
**Discussions version 2.**
Additional file 7:
**Recommendations version 1.**
Additional file 8:
**Consensus Methods version 3.**
Additional file 9:
**Recommendations version 2.**
Additional file 10:
**Discussion version 2.**
Additional file 11:
**Questionnaire 2.**
Additional file 12:
**Questionnaire 3.**
Additional file 13:
**Questionnaire 4.**
Additional file 14:
**Discussion version 4.**
Additional file 15:
**Questionnaire 5.**
Additional file 16:
**Final results.**
