# Supplementary material for: Recommendations for research studies on treatment of idiopathic scoliosis: Consensus 2014 between SOSORT and SRS non–operative management committee
Source: Scoliosis. 2015 Mar 7;10:8. doi: 10.1186/s13013-014-0025-4 (PMC4360938; doi:10.1186/s13013-014-0025-4)
Supplement: Additional file 2: — Consensus Methods version 1. [file 13013_2014_25_MOESM2_ESM.doc]

Questions for you in yellow. Please, feel free to propose any change to the text, using the revision function of word (already activated)

(ref) means references to be added

# First joint SOSORT – SRS Consensus 2014. Inclusion Criteria for clinical studies on Orthopedic and Rehabilitation Treatments of Idiopathic Scoliosis

1. Do you agree with the title ?
2. What is best/correct ? Can we write in general SRS, or it’s correct to write only SRS Non Operative Committee ?
3. I propose to work at this time only on the Inclusion Criteria, since working on the Outcome Criteria would require an extensive search of the literature to compare and chose the best outcome criteria: this is a very difficult exercise particularly if we look at Quality of Life or Esthetics…Outcome Criteria could be the aim of the Second Joint SOSORT-SRS Consensus. Do you agree ?

## Introduction

The evolution of knowledge in Medicine relies mainly on the research development carried out by single groups, but the introduction of statistical techniques allowing to perform in general meta-studies, and specifically meta-analysis, permit today to reach higher level of evidence joining little studies in bigger ones (ref). In this evolution, respecting strict inclusion criteria, possibly internationally defined, is very important to allow proper meta-studies to be performed.

The international Society on Scoliosis Orthopedic and Rehabilitation Treatment (SOSORT), after verifying the gradual reduction of scientific research in the area of the so-called conservative treatment (ref), started producing Consensuses with the aim of reaching a minimum agreement among scientists and clinicians engaged in the field (ref). In this respect, it published the 2011 Clinical Guidelines that offer a general framework of reference to clinicians (ref).

The Scoliosis Research Society (SRS) – Non-Operative Committee (SRS-NOC) published in 1995 the SRS Criteria for Bracing Studies, that constitute the first effort to define precise criteria for research (ref). After this development, a series of papers have been produced respecting these inclusion criteria (ref), and a first meta-analysis has been proposed (ref). Also a RCT (BrAIST Study) has been planned accordingly (ref), even if the inclusion criteria had to be enlarged due to difficulties in recruitment (ref). The main strength of the SRS criteria is in focusing research on the most important population; the main limitation is that all research in other populations, that in any case have to be performed to increase knowledge in this specific research area, have no strict indication, so precluding the possibility to perform meta-studies.

Since the 2^nd^ SOSORT Meeting in Athens 2007 the SRS Presidents have been hosted by SOSORT to start a collaboration between the two Societies (ref). In the 48^th^ SRS Meeting in Lyon 2013, the SRS-NOC and SOSORT had the first combined educational Meeting. In this occasion it was decided to create a Committee to prepare the first joint SOSORT/SRS-NOC Consensus, with the aim to answer to the needs previously presented.

The aim of this paper is to present the results of a Consensus among the best experts of Conservative Treatment of Idiopathic Scoliosis, as represented by SOSORT and SRS-NOC, on the inclusion criteria for studies on Non-Operative approaches.

1. Do you agree with this introduction ?

## Methods

### Design

A Delphi Consensus to develop a reference scheme for the inclusion criteria for clinical studies on Orthopedic and Rehabilitation Treatments of Idiopathic Scoliosis.

### The reference scheme

The main idea beyond the reference scheme for research studies, is to solicit authors writing their contribution focusing on meaningful clinical populations. Even if we solicit authors to focus on these populations, this effort does not mean that they should limit their works to these groups of patients; in any case, producing data with different groupings, we require in any case authors to produce their data also according to this reference scheme, so to facilitate future metanalysis and pooling of data.

This reference scheme is based on the actual knowledge about:

- aims of treatment, that include immediate aesthetics and pain, and risk of future health problems (ref);
- prognosis of IS, that is based mainly on the age at discovery and Cobb degrees (ref);
- risk of health problems, that comes from the Cobb degrees (ref);

In terms of aims of treatment, the reference scheme consider aesthetics, pain and Cobb degrees, the last being the most important prognostic factors for health problems in the future (ref).

While pain and deformity are considered in all ages, aesthetic is considered an important aim from Risser 3-4 stages (European Risser 3) when the patients acquire knowledge and conscience of his/her aesthetic: from this age, treatments exclusively for aesthetic reasons only could be proposed, while before the main focus is on limiting the possible evolution of deformity.

In terms of age, we propose for conservative treatment the classification that split IS in Infantile (age 0-2), Juvenile 1 (age 3-4), 2 (age 5-7) and 3 (age 8-9), and Adolescent from age 10. There are many reasons for this decision: in fact, the prognosis of these curves is different (apart some auto-resolving cases in Infantiles, the earlier the appearance, the worst the prognosis), as well as the evolution and management: Infantiles are rapidly evolving due the rapid growth, and require closer attention, than Juveniles; moreover, Juvenile 1 can still be rapidly evolving, while this is not any more true in Juveniles 2 and 3. All these differences make conservative management different. Also in terms of treatment, there are differences, since active approaches based on exercises cannot be proposed before age 7-8, that is the passage from Juveniles 2 to 3. Due to all these reasons, the actually apparently prevailing classification of Early Onset Scoliosis for what is not Adolescent IS (ref), is not considered valid in the context of conservative treatment.

In splitting Adolescent curves, we considered valid the original SRS criteria (ref), so we had an anchor according to the following inclusion criteria: Age 10, Risser 0-2, curves 25°-40° Cobb, no more that 1 years post-menarche (ref). We do not use here only menarche, since it has been shown that it is not a really valid reference point (ref).

Risser staging is listed considering either the original US version, and the modified European version (ref): in fact, in Europe Risser 2 (partial coverage of the iliac crest by its apophysis) includes US Risser 2 (50% coverage) and 3 (75%), while European Risser 3 is equal to US Risser 4 (100% coverage); moreover, in Europe Risser 4 is starting of fusion, while Risser 5 is complete fusion of the iliac crest apophysis. This difference came from Stagnara, and is widely used in clinical studies in Europe.

Finally, two specific clinical situations have been added to the classification, beyond the general one based on age:

- Flexed posture: this is the situation in which the ability of the spine to counteract the gravity force is failed, and the patients is not any more able to recover a normal standing posture (ref).
- Pregnancy: this specific period is possibly coincident with a progression of the curve (ref), even if this is not well documented in the literature. We propose to consider this stage important for at least one year after delivery, i.e. two years from start of pregnancy.

The degree of deformity has been considered important according to the actual knowledge on adult consequences of IS. We are now aware of three main thresholds, that have been considered important:

- 11° Cobb: significant for IS definition (ref)
- 30° Cobb: as far as we know, below this threshold IS do not create problems in adulthood in terms of back pain and evolution of deformity (ref)
- 45°-50°: surgical threshold (ref)

Consequently, the deformity according to Cobb degrees has been categorized as follows:

- 0: very low degree (and very low clinical importance)
- L: low degree, preventive importance to avoid the 30° threshold (ref)
- M: medium degree, therapeutic importance: a reduction of deformity is searched (if possible), with the absolute aim of avoiding surgery (ref)
- H: high degree: a reduction is strongly searched to avoid surgery, if possible (ref) – surgical threshold if conservative treatment fails
- VH: only for Adolescent with Risser 0-4 (European 3), where the evolution is very high and this threshold is even more important then H

These degrees of deformity have been considered variable according to age and clinical situations, considered in term of risk of progression.

### Consensus Procedure

The Consensus reaching procedure will follow the Delphi principles (ref). All stages will be coordinated by the main author with a Committee involving another member nominated by the SOSORT Board and two members nominated by the SRS. The procedure will include four stages as follows.

#### First stage: SOSORT Board and SRS Non Operative Committee

A first methodological document will be drafted by the Committee including the proposal for the reference scheme of the Consensus. It will be sent to all members of the SOSORT Board and the SRS-NOC to receive suggestions for improvement. As soon as a first Consensus will be achieved, a specific questionnaire will be developed to achieve the best possible Consensus. A Consensus of 75% of agreement will be considered enough for a choice to be immediately accepted. If the this agreement will not be reached, the two most voted choices will be maintained and a second round performed to chose the most preferred.

#### Second stage: SOSORT members and ?

The agreed document will be submitted to all members of SOSORT with a questionnaire to receive other suggestions. After collecting the suggestions and modifying the document accordingly, a questionnaire will be sent. A Consensus of 75% of agreement will be considered enough for a choice to be immediately accepted. If the this agreement will not be reached, the two most voted choices will be maintained and a second round performed to chose the most preferred.

1. Do we leave this stage only at SOSORT or shall we introduce somehow also the SRS members, or part of them (e.g. past SRS Non Operative Committee Presidents/members – I mean, we need tro introduce the SRS members interested in Conservative) ?

#### Third stage: SOSORT Consensus Session during SOSORT Meeting

The final document will be sent, before next SOSORT Meeting, to all participants. It will be presented during the Consensus session and participants will vote their agreement and give their final suggestions.

1. Do we leave this stage only at SOSORT ? I think so, since I do not see how to involve at this stage the SRS…

#### Fourth stage: SOSORT and SRS Boards

The final document will be formally approved by the SOSORT and SRS Boards.

1. SRS-NOC or SRS Board ?
2. Any other suggestion at this stage ?
