# Supplementary material for: Recommendations for research studies on treatment of idiopathic scoliosis: Consensus 2014 between SOSORT and SRS non–operative management committee
Source: Scoliosis. 2015 Mar 7;10:8. doi: 10.1186/s13013-014-0025-4 (PMC4360938; doi:10.1186/s13013-014-0025-4)
Supplement: Additional file 5: — Questionnaire 1. [file 13013_2014_25_MOESM5_ESM.doc]

# First round questionnaire

# SOSORT – SRS Consensus 2014. Inclusion Criteria of non-operative treatment of Idiopathic Scoliosis

We are defining the inclusion criteria for Conservative (Non Operative) Treatment studies. This means that all the following definitions relate only to Conservative Treatment. Please, consider that Conservative Treatment include not only bracing, but also exercises and other treatments. Moreover, please consider that research doesn’t mean clinical application: obviously we should be able to make research in many different group of patients, but grouping them will give a framework of reference for all our future searches.

Please, read the attached document, with the methodology of this year Consensus, if you want to better understand all the program.

The aim of this process is to build a scheme like that in the next page (fully based on the 2011 SOSORT Guidelines), where the group of patients are classified according to the age and the importance of the deformity (expressed in terms of Cobb degrees), the aesthetic impact and the presence of pain. This scheme is a first proposal (see the attached document to better understand how it has been defined). Please answer to the following questionnaire to reach this aim.

|  |  |  | **Deformity** | | | | | | | | | | | | **Aesthetic compromise** | **Pain** |
| --- | --- | --- | --- | --- | --- | --- | --- | --- | --- | --- | --- | --- | --- | --- | --- | --- |
|  |  |  | *Hump >4°* | *10°* | *15°* | *20°* | *25°* | *30°* | *35°* | *40°* | *45°* | *50°* | *-->* |  |  |  |
| IN | **Infantile** | Age 0-2 | 0 | L | | | M | | | | H | | | |  | P |
| J1 | **Juvenile 1** | *Age 3-4* | 0 | L | | | M | | | | H | | | |  | P |
| J2 | **Juvenile 2** | *Age 5-7* | 0 | L | | | M | | | H | | VH | | |  | P |
| J3 | **Juvenile 3** | *Age 8-9* | 0 | L | | | M | | | H | | VH | | |  | P |
| A0 | **Adolescent** | *Risser 0-2* | 0 | L | | | M (SRS) | | | H | | VH | | |  | P |
| A3 | **Adolescent** | *Risser 3-4 (European 3)* | 0 | | L | | M | | | | H | | | | E | P |
| A4 | **Adolescent** | *European Risser 4* | 0 | | | L | | M | | | H | | | | E | P |
| AD | **Adult** | *Risser 5 to menopause (55 years)* | 0 | | | | | L | | | H | | | | E | P |
| PM | **Post-Menopause** | *Age 55-70* | 0 | | | | | L | | | H | | | | E | P |
| EL | **Elderly** | *Age above 70* | 0 | | | | | L | | | H | | | | E | P |
| FP | **Flexed posture** |  | 0 | | | L | | M | | | H | | | |  | P |
| PR | **Pregnancy** | *0-2 years from start of pregnancy* | 0 | | | L | | M | | | H | | | |  | P |

# First round questionnaire

# General

1. Do you agree with the title of this Consensus: SOSORT – SRS Consensus 2014. Inclusion Criteria of non-operative treatment of Idiopathic Scoliosis ?

<> Yes <> No Suggestions: _____________________________________________ ________________________________________________________________________________________________________________________________________________________________

1. What do you prefer to define the so-called non-operative treatment of scoliosis ?

<> by exclusion (e.g. conservative, non-operative)

<> by inclusion (e.g. orthopedic, rehabilitation…) Suggestions: ______________________ ________________________________________________________________________________________________________________________________________________________________

1. Which one of the following is your preferred definition of the so-called conservative / non-operative treatment of scoliosis ?

<> conservative <> non-operative <> orthopedic

<> rehabilitation <> orthopedic and rehabilitation <> physiotherapic

Suggestions: _____________________________________________________________________ ________________________________________________________________________________________________________________________________________________________________

# The reference scheme (in yellow the actual text)

The main idea beyond the reference scheme for research studies, is to solicit authors writing their contribution focusing on meaningful clinical populations. Even if we solicit authors to focus on these populations, this effort does not mean that they should limit their works to these groups of patients; in any case, producing data with different groupings, we require in any case authors to produce their data also according to this reference scheme, so to facilitate future metanalysis and pooling of data.

1. Do you agree with this statement ?

<> Yes <> No Suggestions: _____________________________________________ ________________________________________________________________________________________________________________________________________________________________

This reference scheme is based on the actual knowledge about:

- aims of treatment, that include immediate aesthetics and pain, and risk of future health problems (ref);
- prognosis of IS, that is based mainly on the age at discovery and Cobb degrees (ref);
- risk of health problems, that comes from the Cobb degrees (ref);

1. Do you agree with this statement ?

<> Yes <> No Suggestions: _____________________________________________ ________________________________________________________________________________________________________________________________________________________________

In terms of aims of treatment, the reference scheme consider aesthetics, pain and Cobb degrees, the last being the most important prognostic factors for health problems in the future (ref).

1. For the Inclusion Criteria of research studies on conservative treatment of idiopathic scoliosis: do you agree on considering the following different therapeutic aims ?

| **Deformity** | **Aesthetic compromise** | **Pain** |
| --- | --- | --- |

<> Yes <> No Suggestions: _____________________________________________ ________________________________________________________________________________________________________________________________________________________________

While pain and deformity are considered in all ages, aesthetic is considered an important aim from Risser 3-4 stages (European Risser 3) when the patients acquire knowledge and conscience of his/her aesthetic: from this age, treatments exclusively for aesthetic reasons only could be proposed, while before the main focus is on limiting the possible evolution of deformity.

1. For the Inclusion Criteria of research studies on conservative treatment of idiopathic scoliosis: do you agree on considering significant aesthetics from the last part of adolescence (Risser 3-4 – European 3) and not before ?

<> Yes <> No Suggestions: _____________________________________________ ________________________________________________________________________________________________________________________________________________________________

In terms of age, we propose for conservative treatment the classification that split IS in Infantile (age 0-2), Juvenile 1 (age 3-4), 2 (age 5-7) and 3 (age 8-9), and Adolescent from age 10. There are many reasons for this decision: in fact, the prognosis of these curves is different (apart some auto-resolving cases in Infantiles, the earlier the appearance, the worst the prognosis), as well as the evolution and management: Infantiles are rapidly evolving due the rapid growth, and require closer attention, than Juveniles; moreover, Juvenile 1 can still be rapidly evolving, while this is not any more true in Juveniles 2 and 3. All these differences make conservative management different. Also in terms of treatment, there are differences, since active approaches based on exercises cannot be proposed before age 7-8, that is the passage from Juveniles 2 to 3. Due to all these reasons, the actually apparently prevailing classification of Early Onset Scoliosis for what is not Adolescent IS (ref), is not considered valid in the context of conservative treatment.

1. For the Inclusion Criteria of research studies on conservative treatment of idiopathic scoliosis: do you agree on avoiding the general term Early Onset Scoliosis for age below 10 years ?

<> Yes <> No Suggestions: _____________________________________________ ________________________________________________________________________________________________________________________________________________________________

1. For the Inclusion Criteria of research studies on conservative treatment of idiopathic scoliosis: do you agree the following general classification ?

| **Infantile** | *Age 0-2* |
| --- | --- |
| **Juvenile 1** | *Agre 3-4* |
| **Juvenile 2** | *Age 5-7* |
| **Juvenile 3** | *Age 8-9* |
| **Adolescent** | *Risser 0-2* |
| **Adolescent** | *Risser 3-4 (European 3)* |
| **Adolescent** | *European Risser 4* |
| **Adult** | *Risser 5 to menopause (55 years)* |
| **Post-Menopause** | *Age 55-70* |
| **Elderly** | *Age above 70* |
| **Elderly** | *Flexed posture* |
| **Adult** | *Pregnancy (0-2 years)* |

<> Yes <> No Suggestions: _____________________________________________ ________________________________________________________________________________________________________________________________________________________________

In splitting Adolescent curves, we considered valid the original SRS criteria (ref), so we had an anchor according to the following inclusion criteria: Age 10, Risser 0-2, curves 25°-40° Cobb, no more that 1 years post-menarche (ref).

1. Do you agree to maintain the SRS-criteria as a stable anchor ?

<> Yes <> No Suggestions: _____________________________________________ ________________________________________________________________________________________________________________________________________________________________

We do not use here only menarche, since it has been shown that it is not a really valid reference point (ref).

1. Do you agree with this statement ?

<> Yes <> No Suggestions: _____________________________________________ ________________________________________________________________________________________________________________________________________________________________

Risser staging is listed considering either the original US version, and the modified European version (ref): in fact, in Europe Risser 2 (partial coverage of the iliac crest by its apophysis) includes US Risser 2 (50% coverage) and 3 (75%), while European Risser 3 is equal to US Risser 4 (100% coverage); moreover, in Europe Risser 4 is starting of fusion, while Risser 5 is complete fusion of the iliac crest apophysis. This difference came from Stagnara, and is widely used in clinical studies in Europe.

1. Do you agree with this statement ?

<> Yes <> No Suggestions: _____________________________________________ ________________________________________________________________________________________________________________________________________________________________

Finally, two specific clinical situations have been added to the classification, beyond the general one based on age:

- Flexed posture: this is the situation in which the ability of the spine to counteract the gravity force is failed, and the patients is not any more able to recover a normal standing posture (ref).
- Pregnancy: this specific period is possibly coincident with a progression of the curve (ref), even if this is not well documented in the literature. We propose to consider this stage important for at least one year after delivery, i.e. two years from start of pregnancy.

1. Do you agree with this decision ?

<> Yes <> No Suggestions: _____________________________________________ ________________________________________________________________________________________________________________________________________________________________

1. For the Inclusion Criteria of research studies on conservative treatment of idiopathic scoliosis: do you agree on considering the first level of deformity and hump >4° with a radiological curve even if below 11° ?

<> Yes <> No Suggestions: _____________________________________________ ________________________________________________________________________________________________________________________________________________________________

The degree of deformity has been considered important according to the actual knowledge on adult consequences of IS. We are now aware of three main thresholds, that have been considered important:

- 11° Cobb: significant for IS definition (ref)
- 30° Cobb: as far as we know, below this threshold IS do not create problems in adulthood in terms of back pain and evolution of deformity (ref)
- 45°-50°: surgical threshold (ref)

1. Do you agree with this statement ?

<> Yes <> No Suggestions: _____________________________________________ ________________________________________________________________________________________________________________________________________________________________

1. For the Inclusion Criteria of research studies on conservative treatment of idiopathic scoliosis: do you agree on considering the first level of deformity and hump >4° with a radiological curve even if below 11° ?

<> Yes <> No Suggestions: _____________________________________________ ________________________________________________________________________________________________________________________________________________________________

Consequently, the deformity according to Cobb degrees has been categorized as follows:

- 0: very low degree (and very low clinical importance)
- L: low degree, preventive importance to avoid the 30° threshold (ref)
- M: medium degree, therapeutic importance: a reduction of deformity is searched (if possible), with the absolute aim of avoiding surgery (ref)
- H: high degree: a reduction is strongly searched to avoid surgery, if possible (ref) – surgical threshold if conservative treatment fails
- VH: only for Adolescent with Risser 0-4 (European 3), where the evolution is very high and this threshold is even more important then H

These degrees of deformity have been considered variable according to age and clinical situations, considered in term of risk of progression.

1. For the Inclusion Criteria of research studies on conservative treatment of idiopathic scoliosis: do you agree on splitting the aim for deformity according to the degree of deformity defined as follows:

| 0 | Very low degree (and very low clinical importance) |
| --- | --- |
| L | Low degree, preventive importance |
| M | Medium degree, therapeuthic importance |
| H | High degree |
| VH | Very high degree (only for Adolescent Risser 0-4) |

<> Yes <> No Suggestions: _____________________________________________ ________________________________________________________________________________________________________________________________________________________________

1. For the Inclusion Criteria of research studies on conservative treatment of idiopathic scoliosis do you agree not to set the highest limit (patients rejecting surgery) ?

<> Yes <> No Suggestions: _____________________________________________ ________________________________________________________________________________________________________________________________________________________________

These degrees of deformity have been considered variable according to age and clinical situations, considered in term of risk of progression.

1. For the Inclusion Criteria of research studies on conservative treatment of idiopathic scoliosis: do you agree on changing the definition of the degree of deformity according to the risk of progression and the aim of treatments (age classification) ? (i.e. 0 is different in different ages, as well as L, M, or H degree curves)

<> Yes <> No Suggestions: _____________________________________________ ________________________________________________________________________________________________________________________________________________________________

## Infantile idiopathic scoliosis (age 0-2)

1. The proposal for a low degree curve is between 10° and 24°. Do you agree ?

<> Yes <> No If you propose another range, please tick your choice below

| <10° but ATR >4° | 10-14° | 15-19° | 20-24° | 25-29° | 30-34° | 35-39° | 40-44° | 45-49° | 50° or more |
| --- | --- | --- | --- | --- | --- | --- | --- | --- | --- |

Suggestions: _____________________________________________________________________ ________________________________________________________________________________________________________________________________________________________________

1. The proposal for a medium degree curve is between 25° and 44°. Do you agree ?

<> Yes <> No If you propose another range, please tick your choice below

| <10° but ATR >4° | 10-14° | 15-19° | 20-24° | 25-29° | 30-34° | 35-39° | 40-44° | 45-49° | 50° or more |
| --- | --- | --- | --- | --- | --- | --- | --- | --- | --- |

Suggestions: _____________________________________________________________________ ________________________________________________________________________________________________________________________________________________________________

1. The proposal for an high degree curve is from 45° and up. Do you agree ?

<> Yes <> No If you propose another range, please tick your choice below

| <10° but ATR >4° | 10-14° | 15-19° | 20-24° | 25-29° | 30-34° | 35-39° | 40-44° | 45-49° | 50° or more |
| --- | --- | --- | --- | --- | --- | --- | --- | --- | --- |

Suggestions: _____________________________________________________________________ ________________________________________________________________________________________________________________________________________________________________

## Juvenile 1 idiopathic scoliosis (age 3-4)

1. The proposal for a low degree curve is between 10° and 24°. Do you agree ?

<> Yes <> No If you propose another range, please tick your choice below

| <10° but ATR >4° | 10-14° | 15-19° | 20-24° | 25-29° | 30-34° | 35-39° | 40-44° | 45-49° | 50° or more |
| --- | --- | --- | --- | --- | --- | --- | --- | --- | --- |

Suggestions: _____________________________________________________________________ ________________________________________________________________________________________________________________________________________________________________

1. The proposal for a medium degree curve is between 25° and 44°. Do you agree ?

<> Yes <> No If you propose another range, please tick your choice below

| <10° but ATR >4° | 10-14° | 15-19° | 20-24° | 25-29° | 30-34° | 35-39° | 40-44° | 45-49° | 50° or more |
| --- | --- | --- | --- | --- | --- | --- | --- | --- | --- |

Suggestions: _____________________________________________________________________ ________________________________________________________________________________________________________________________________________________________________

1. The proposal for an high degree curve is from 45° and up. Do you agree ?

<> Yes <> No If you propose another range, please tick your choice below

| <10° but ATR >4° | 10-14° | 15-19° | 20-24° | 25-29° | 30-34° | 35-39° | 40-44° | 45-49° | 50° or more |
| --- | --- | --- | --- | --- | --- | --- | --- | --- | --- |

Suggestions: _____________________________________________________________________ ________________________________________________________________________________________________________________________________________________________________

## Juvenile 2 idiopathic scoliosis (age 6-7)

1. The proposal for a low degree curve is between 10° and 24°. Do you agree ?

<> Yes <> No If you propose another range, please tick your choice below

| <10° but ATR >4° | 10-14° | 15-19° | 20-24° | 25-29° | 30-34° | 35-39° | 40-44° | 45-49° | 50° or more |
| --- | --- | --- | --- | --- | --- | --- | --- | --- | --- |

Suggestions: _____________________________________________________________________ ________________________________________________________________________________________________________________________________________________________________

1. The proposal for a medium degree curve is between 25° and 39°. Do you agree ?

<> Yes <> No If you propose another range, please tick your choice below

| <10° but ATR >4° | 10-14° | 15-19° | 20-24° | 25-29° | 30-34° | 35-39° | 40-44° | 45-49° | 50° or more |
| --- | --- | --- | --- | --- | --- | --- | --- | --- | --- |

Suggestions: _____________________________________________________________________ ________________________________________________________________________________________________________________________________________________________________

1. The proposal for an high degree curve is between 40° and 49°. Do you agree ?

<> Yes <> No If you propose another range, please tick your choice below

| <10° but ATR >4° | 10-14° | 15-19° | 20-24° | 25-29° | 30-34° | 35-39° | 40-44° | 45-49° | 50° or more |
| --- | --- | --- | --- | --- | --- | --- | --- | --- | --- |

Suggestions: _____________________________________________________________________ ________________________________________________________________________________________________________________________________________________________________

1. The proposal for a very high degree curve is from 50° and up. Do you agree ?

<> Yes <> No If you propose another range, please tick your choice below

| <10° but ATR >4° | 10-14° | 15-19° | 20-24° | 25-29° | 30-34° | 35-39° | 40-44° | 45-49° | 50° or more |
| --- | --- | --- | --- | --- | --- | --- | --- | --- | --- |

Suggestions: _____________________________________________________________________ ________________________________________________________________________________________________________________________________________________________________

## Juvenile 3 idiopathic scoliosis (age 8-9)

1. The proposal for a low degree curve is between 10° and 24°. Do you agree ?

<> Yes <> No If you propose another range, please tick your choice below

| <10° but ATR >4° | 10-14° | 15-19° | 20-24° | 25-29° | 30-34° | 35-39° | 40-44° | 45-49° | 50° or more |
| --- | --- | --- | --- | --- | --- | --- | --- | --- | --- |

Suggestions: _____________________________________________________________________ ________________________________________________________________________________________________________________________________________________________________

1. The proposal for a medium degree curve is between 25° and 39°. Do you agree ?

<> Yes <> No If you propose another range, please tick your choice below

| <10° but ATR >4° | 10-14° | 15-19° | 20-24° | 25-29° | 30-34° | 35-39° | 40-44° | 45-49° | 50° or more |
| --- | --- | --- | --- | --- | --- | --- | --- | --- | --- |

Suggestions: _____________________________________________________________________ ________________________________________________________________________________________________________________________________________________________________

1. The proposal for an high degree curve is between 40° and 49°. Do you agree ?

<> Yes <> No If you propose another range, please tick your choice below

| <10° but ATR >4° | 10-14° | 15-19° | 20-24° | 25-29° | 30-34° | 35-39° | 40-44° | 45-49° | 50° or more |
| --- | --- | --- | --- | --- | --- | --- | --- | --- | --- |

Suggestions: _____________________________________________________________________ ________________________________________________________________________________________________________________________________________________________________

1. The proposal for a very high degree curve is from 50° and up. Do you agree ?

<> Yes <> No If you propose another range, please tick your choice below

| <10° but ATR >4° | 10-14° | 15-19° | 20-24° | 25-29° | 30-34° | 35-39° | 40-44° | 45-49° | 50° or more |
| --- | --- | --- | --- | --- | --- | --- | --- | --- | --- |

Suggestions: _____________________________________________________________________ ________________________________________________________________________________________________________________________________________________________________

## Adolescent idiopathic scoliosis (age 10 or more, Risser 0-2)

1. The proposal for a low degree curve is between 10° and 24°. Do you agree ?

<> Yes <> No If you propose another range, please tick your choice below

| <10° but ATR >4° | 10-14° | 15-19° | 20-24° | 25-29° | 30-34° | 35-39° | 40-44° | 45-49° | 50° or more |
| --- | --- | --- | --- | --- | --- | --- | --- | --- | --- |

Suggestions: _____________________________________________________________________ ________________________________________________________________________________________________________________________________________________________________

1. The proposal for a medium degree curve is between 25° and 39° (the actual SRS-criteria). Do you agree ?

<> Yes <> No If you propose another range, please tick your choice below

| <10° but ATR >4° | 10-14° | 15-19° | 20-24° | 25-29° | 30-34° | 35-39° | 40-44° | 45-49° | 50° or more |
| --- | --- | --- | --- | --- | --- | --- | --- | --- | --- |

Suggestions: _____________________________________________________________________ ________________________________________________________________________________________________________________________________________________________________

1. The proposal for an high degree curve is between 40° and 49°. Do you agree ?

<> Yes <> No If you propose another range, please tick your choice below

| <10° but ATR >4° | 10-14° | 15-19° | 20-24° | 25-29° | 30-34° | 35-39° | 40-44° | 45-49° | 50° or more |
| --- | --- | --- | --- | --- | --- | --- | --- | --- | --- |

Suggestions: _____________________________________________________________________ ________________________________________________________________________________________________________________________________________________________________

1. The proposal for a very high degree curve is from 50° and up. Do you agree ?

<> Yes <> No If you propose another range, please tick your choice below

| <10° but ATR >4° | 10-14° | 15-19° | 20-24° | 25-29° | 30-34° | 35-39° | 40-44° | 45-49° | 50° or more |
| --- | --- | --- | --- | --- | --- | --- | --- | --- | --- |

Suggestions: _____________________________________________________________________ ________________________________________________________________________________________________________________________________________________________________

## Adolescent idiopathic scoliosis (Risser 3-4 – European 3)

1. The proposal for a low degree curve is between 15° and 24°. Do you agree ?

<> Yes <> No If you propose another range, please tick your choice below

| <10° but ATR >4° | 10-14° | 15-19° | 20-24° | 25-29° | 30-34° | 35-39° | 40-44° | 45-49° | 50° or more |
| --- | --- | --- | --- | --- | --- | --- | --- | --- | --- |

Suggestions: _____________________________________________________________________ ________________________________________________________________________________________________________________________________________________________________

1. The proposal for a medium degree curve is between 25° and 44°. Do you agree ?

<> Yes <> No If you propose another range, please tick your choice below

| <10° but ATR >4° | 10-14° | 15-19° | 20-24° | 25-29° | 30-34° | 35-39° | 40-44° | 45-49° | 50° or more |
| --- | --- | --- | --- | --- | --- | --- | --- | --- | --- |

Suggestions: _____________________________________________________________________ ________________________________________________________________________________________________________________________________________________________________

1. The proposal for an high degree curve is from 45° and up. Do you agree ?

<> Yes <> No If you propose another range, please tick your choice below

| <10° but ATR >4° | 10-14° | 15-19° | 20-24° | 25-29° | 30-34° | 35-39° | 40-44° | 45-49° | 50° or more |
| --- | --- | --- | --- | --- | --- | --- | --- | --- | --- |

Suggestions: _____________________________________________________________________ ________________________________________________________________________________________________________________________________________________________________

## Adolescent idiopathic scoliosis (Risser 4)

1. The proposal for a low degree curve is between 20° and 29°. Do you agree ?

<> Yes <> No If you propose another range, please tick your choice below

| <10° but ATR >4° | 10-14° | 15-19° | 20-24° | 25-29° | 30-34° | 35-39° | 40-44° | 45-49° | 50° or more |
| --- | --- | --- | --- | --- | --- | --- | --- | --- | --- |

Suggestions: _____________________________________________________________________ ________________________________________________________________________________________________________________________________________________________________

1. The proposal for a medium degree curve is between 30° and 44°. Do you agree ?

<> Yes <> No If you propose another range, please tick your choice below

| <10° but ATR >4° | 10-14° | 15-19° | 20-24° | 25-29° | 30-34° | 35-39° | 40-44° | 45-49° | 50° or more |
| --- | --- | --- | --- | --- | --- | --- | --- | --- | --- |

Suggestions: _____________________________________________________________________ ________________________________________________________________________________________________________________________________________________________________

1. The proposal for an high degree curve is from 45° and up. Do you agree ?

<> Yes <> No If you propose another range, please tick your choice below

| <10° but ATR >4° | 10-14° | 15-19° | 20-24° | 25-29° | 30-34° | 35-39° | 40-44° | 45-49° | 50° or more |
| --- | --- | --- | --- | --- | --- | --- | --- | --- | --- |

Suggestions: _____________________________________________________________________ ________________________________________________________________________________________________________________________________________________________________

## Adult idiopathic scoliosis (Risser 5 to menopause / age 55)

1. The proposal for a low degree curve is between 30° and 44°. Do you agree ?

<> Yes <> No If you propose another range, please tick your choice below

| <10° but ATR >4° | 10-14° | 15-19° | 20-24° | 25-29° | 30-34° | 35-39° | 40-44° | 45-49° | 50° or more |
| --- | --- | --- | --- | --- | --- | --- | --- | --- | --- |

Suggestions: _____________________________________________________________________ ________________________________________________________________________________________________________________________________________________________________

1. There is no proposal for a medium degree curve. Do you agree ? If not, thick your choice:

<> Yes <> No If you propose another range, please tick your choice below

| <10° but ATR >4° | 10-14° | 15-19° | 20-24° | 25-29° | 30-34° | 35-39° | 40-44° | 45-49° | 50° or more |
| --- | --- | --- | --- | --- | --- | --- | --- | --- | --- |

Suggestions: _____________________________________________________________________ ________________________________________________________________________________________________________________________________________________________________

1. The proposal for an high degree curve is from 45° and up. Do you agree ?

<> Yes <> No If you propose another range, please tick your choice below

| <10° but ATR >4° | 10-14° | 15-19° | 20-24° | 25-29° | 30-34° | 35-39° | 40-44° | 45-49° | 50° or more |
| --- | --- | --- | --- | --- | --- | --- | --- | --- | --- |

Suggestions: _____________________________________________________________________ ________________________________________________________________________________________________________________________________________________________________

## Post-menopause idiopathic scoliosis (age 55-70)

1. The proposal for a low degree curve is between 30° and 44°. Do you agree ?

<> Yes <> No If you propose another range, please tick your choice below

| <10° but ATR >4° | 10-14° | 15-19° | 20-24° | 25-29° | 30-34° | 35-39° | 40-44° | 45-49° | 50° or more |
| --- | --- | --- | --- | --- | --- | --- | --- | --- | --- |

Suggestions: _____________________________________________________________________ ________________________________________________________________________________________________________________________________________________________________

1. There is no proposal for a medium degree curve. Do you agree ? If not, thick your choice:

<> Yes <> No If you propose another range, please tick your choice below

| <10° but ATR >4° | 10-14° | 15-19° | 20-24° | 25-29° | 30-34° | 35-39° | 40-44° | 45-49° | 50° or more |
| --- | --- | --- | --- | --- | --- | --- | --- | --- | --- |

Suggestions: _____________________________________________________________________ ________________________________________________________________________________________________________________________________________________________________

1. The proposal for an high degree curve is from 45° and up. Do you agree ?

<> Yes <> No If you propose another range, please tick your choice below

| <10° but ATR >4° | 10-14° | 15-19° | 20-24° | 25-29° | 30-34° | 35-39° | 40-44° | 45-49° | 50° or more |
| --- | --- | --- | --- | --- | --- | --- | --- | --- | --- |

Suggestions: _____________________________________________________________________ ________________________________________________________________________________________________________________________________________________________________

## Elderly idiopathic scoliosis (age above 70)

1. The proposal for a low degree curve is between 30° and 44°. Do you agree ?

<> Yes <> No If you propose another range, please tick your choice below

| <10° but ATR >4° | 10-14° | 15-19° | 20-24° | 25-29° | 30-34° | 35-39° | 40-44° | 45-49° | 50° or more |
| --- | --- | --- | --- | --- | --- | --- | --- | --- | --- |

Suggestions: _____________________________________________________________________ ________________________________________________________________________________________________________________________________________________________________

1. There is no proposal for a medium degree curve. Do you agree ? If not, thick your choice:

<> Yes <> No If you propose another range, please tick your choice below

| <10° but ATR >4° | 10-14° | 15-19° | 20-24° | 25-29° | 30-34° | 35-39° | 40-44° | 45-49° | 50° or more |
| --- | --- | --- | --- | --- | --- | --- | --- | --- | --- |

Suggestions: _____________________________________________________________________ ________________________________________________________________________________________________________________________________________________________________

1. The proposal for an high degree curve is from 45° and up. Do you agree ?

<> Yes <> No If you propose another range, please tick your choice below

| <10° but ATR >4° | 10-14° | 15-19° | 20-24° | 25-29° | 30-34° | 35-39° | 40-44° | 45-49° | 50° or more |
| --- | --- | --- | --- | --- | --- | --- | --- | --- | --- |

Suggestions: _____________________________________________________________________ ________________________________________________________________________________________________________________________________________________________________

## Elderly flexed posture idiopathic scoliosis

1. The proposal for a low degree curve is between 20° and 29°. Do you agree ?

<> Yes <> No If you propose another range, please tick your choice below

| <10° but ATR >4° | 10-14° | 15-19° | 20-24° | 25-29° | 30-34° | 35-39° | 40-44° | 45-49° | 50° or more |
| --- | --- | --- | --- | --- | --- | --- | --- | --- | --- |

Suggestions: _____________________________________________________________________ ________________________________________________________________________________________________________________________________________________________________

1. The proposal for a medium degree curve is between 30° and 44°. Do you agree ?

<> Yes <> No If you propose another range, please tick your choice below

| <10° but ATR >4° | 10-14° | 15-19° | 20-24° | 25-29° | 30-34° | 35-39° | 40-44° | 45-49° | 50° or more |
| --- | --- | --- | --- | --- | --- | --- | --- | --- | --- |

Suggestions: _____________________________________________________________________ ________________________________________________________________________________________________________________________________________________________________

1. The proposal for an high degree curve is from 45° and up. Do you agree ?

<> Yes <> No If you propose another range, please tick your choice below

| <10° but ATR >4° | 10-14° | 15-19° | 20-24° | 25-29° | 30-34° | 35-39° | 40-44° | 45-49° | 50° or more |
| --- | --- | --- | --- | --- | --- | --- | --- | --- | --- |

Suggestions: _____________________________________________________________________ ________________________________________________________________________________________________________________________________________________________________

## Pregnancy idiopathic scoliosis (0-2 years from start of pregnancy)

1. The proposal for a low degree curve is between 20° and 29°. Do you agree ?

<> Yes <> No If you propose another range, please tick your choice below

| <10° but ATR >4° | 10-14° | 15-19° | 20-24° | 25-29° | 30-34° | 35-39° | 40-44° | 45-49° | 50° or more |
| --- | --- | --- | --- | --- | --- | --- | --- | --- | --- |

Suggestions: _____________________________________________________________________ ________________________________________________________________________________________________________________________________________________________________

1. The proposal for a medium degree curve is between 30° and 44°. Do you agree ?

<> Yes <> No If you propose another range, please tick your choice below

| <10° but ATR >4° | 10-14° | 15-19° | 20-24° | 25-29° | 30-34° | 35-39° | 40-44° | 45-49° | 50° or more |
| --- | --- | --- | --- | --- | --- | --- | --- | --- | --- |

Suggestions: _____________________________________________________________________ ________________________________________________________________________________________________________________________________________________________________

1. The proposal for an high degree curve is from 45° and up. Do you agree ?

<> Yes <> No If you propose another range, please tick your choice below

| <10° but ATR >4° | 10-14° | 15-19° | 20-24° | 25-29° | 30-34° | 35-39° | 40-44° | 45-49° | 50° or more |
| --- | --- | --- | --- | --- | --- | --- | --- | --- | --- |

Suggestions: _____________________________________________________________________ ________________________________________________________________________________________________________________________________________________________________

1. Do you have any other suggestion ?

Suggestions: _____________________________________________________________________ ________________________________________________________________________________________________________________________________________________________________________________________________________________________________________________________________________________________________________________________________
