# Supplementary material for: Recommendations for research studies on treatment of idiopathic scoliosis: Consensus 2014 between SOSORT and SRS non–operative management committee
Source: Scoliosis. 2015 Mar 7;10:8. doi: 10.1186/s13013-014-0025-4 (PMC4360938; doi:10.1186/s13013-014-0025-4)
Supplement: Additional file 6: — Discussions version 2. [file 13013_2014_25_MOESM6_ESM.doc]

# Discussions opened

## Use of the comprehensive term non-operative instead of conservative

*Stefano Negrini originally proposed the classical term “conservative”, that is now changed to “non-operative”*.

**Tim Hresko.** As mentioned elsewhere, clear definitions of terms is mandatory. “non-operative” is a very explicit term.. “Conservative” is a value judgment , subjective term. Many people, including my mentor Dr John Hall, would say “conservative to what”. Bracing is very radical , not conservative, to some of my patients. I think the term conservative is not precise.

2^nd^ point: Miniamlly invasive surgery is coming. Some would call MIS conservative relative to spinal fusion—I may be one of those persons. Very soon, MIS surgery will be compared to bracing and other non operative treatments. The same inclusion criteria we develop should be used by the MIS tethering proponents . So , I would propose that we consider terms such as “non- fusion”, “motion preservation” “ Functional treatment” though out the process. We then strictly define the terms and the rational for the terms.

**Stefano Negrini**. My preferred term, according to the name of our Society, would be “orthopedic and rehabilitation treatment”, since either “conservative” and “non-operative” define by exclusion. To me, a definition should define what is done, and not “everything apart another thing” (I mean, everything apart surgery). In the main time, the old term “conservative” include all what is today in the field of orthopedic medicine (not-surgical) but also “rehabilitation medicine”. That’s the reason for my proposal.

## Classification according to age

*Sentence in discussion. In terms of age, we propose for non-operative treatment the classification that split IS in Infantile (age 0-2), Juvenile 1 (age 3-4), 2 (age 5-7) and 3 (age 8-9), and Adolescent from age 10*

**Nigel Price.** Stefano-is this splitting of age groups a SOSORT consensus? I am familiar with the concept of early onset being less than 5 years-a concept the the Early Onset Scoliosis Group considers important-are the groups really heterogeneous?

**Stefano Negrini.** We re-introduced this classification in the SOSORT Guidelines. In reality, a classification should help for prognosis and treatment. In conservative, we face aggressive curves due to rapid growth: this is true until age 4, when growth slows down. In the past this classification was created, we could come out with a new one, but really in non-operative not splitting these groups could be a limitation. Nevertheless, this should be a point to be thoroughly discussed and we must maintain this point open to discussion. Tim proposed to join Infantile and Juvenile 1: the main difference here, to me, is that in the first year of age you can still have the self-resolving, that is not true afterwards. Really, I would like to know the opinion of the others as well before chosing.

1Tim: why not include Juvenile 1 with the infantile? To me, there is a big difference since in the first year of age you can still have the self-resolving, that is not true afterwards. Really, I would like to know the opinion of the others as well before chosing.

**Tim Hresko.** Early onset scoliosis is avery common term used by SRS members and of great interest due to concern for chest growth, pulmonary function when fusion is perform in less than age 8. Can we use the term EOS but then subclassifiy it into EOS- infantile and EOS- juvenile 1( under age 7, too early for exercise approaches) and juvenile 2( old enough for exercise)? Again, to be discussed.

## Risser sign and other signs of bone age

***Sentence in discussion****. Risser staging is listed considering either the original US version, and the modified European version (ref): in fact, in Europe Risser 2 (partial coverage of the iliac crest by its apophysis) includes US Risser 2 (50% coverage) and 3 (75%), while European Risser 3 is equal to US Risser 4 (100% coverage); moreover, in Europe Risser 4 is starting of fusion, while Risser 5 is complete fusion of the iliac crest apophysis. This difference came from Stagnara, and is widely used in clinical studies in Europe.*

**Nigel Price.** Future studies need to break out Risser 0, triradiate cartilge open ( very high risk) from the Risser 2, also Risser classification of Risser 2 is very unreliable and here I agree with European partial coverage.

**Stefano Negrini** I agree on this point: in fact we could introduce this concept in the text. Nevertheless, we run the risk to enter a little too much into the details if we considered that the aim is to offer a general scheme.

**Nigel Price**. This may be an opportunity to start using elbow ulnar apophysis and hand age criteria.

**Stefano Negrini**. The same as above for triradiate cartilage. The other problem here is that elbow ulnar apophysis, as far as I know, has been proposed by Dimeglio but it is not validated. Am I wrong ? Moreover, it is not visible in most of cases in the regular x-rays, while , in the new proposed positioning of the hands for the EOS system it is not visible by definition.

## Concept of flexed posture

***Sentence in discussion****. Flexed posture: this is the situation in which the ability of the spine to counteract the gravity force is failed, and the patients is not any more able to recover a normal standing posture (ref).*

**Tim Hresko**. Are you proposing the use of sagittal xray with positive balance? 3D classification, SRS is slowly moving to 3D classification but not there yet. The EOS radiography will change this rapidly. Should be anticipate this happening and include this concept. IS 3 D concept in the SOSORT philosophy?

**Stefano Negrini**. The term “flexed posture” or the term “camptocormia” are quite widely used to define patients who are not able to maintain the normal erect posture, either during gait or in normal standing. I am not here proposing to make such a jump as using a 3D classification that still does not exist: nevertheless these patients in “flexed posture” or “camptocormia” have specific difference from all the others, and need different studies. Perhaps a better definition could be important.

**Steve Glassman.** I am a bit confused regarding the "flexed posture" designation for the older adults. I assume that this refers to sagittal imbalance, and if so, maybe we need to examine that terminology.

**Stefano Negrini.** You're correct about flexed posture. In reality this is a terminology widely used in PRM, regarding all patients with sagittal imbalance driving the spine to bend anteriorly (lumbar kyphosis, but also thoracic hyperkyphosis), including Parkinson camptocormia, osteoporosis multiple fractures, spinal stenosis, important scoliosis, and so on. In the past also triple flexion (I don't know if this is correct in English, but it is in latin countries, ie Italy and France) was used, meaning flexion of the hip, knees and ankles, always combining with a flexed posture of the spine (ie lumbar kyphosis): after the studies by Roussouly et al we then discovered that the hip in reality was extended to the maximum and not flexed, but this is another history. Among spinal surgeons now the term sagittal imbalance prevails, but it also refers to other aspects including wrong relationships among spinal and pelvic parameters. Perhaps we could decide to avoid including the adult spine in this consensus, but in a SOSORT perspective (and generally speaking non-operative perspective) also the adult spine must be studied, since there are very few studies until now.

# Thresholds of scoliosis

***Sentence in discussion****. We are now aware of three main thresholds, that have been considered important:*

- *11° Cobb: significant for IS definition (ref)*
- *30° Cobb: as far as we know, below this threshold IS do not create problems in adulthood in terms of back pain and evolution of deformity (ref)*
- *45°-50°: surgical threshold (ref)*

**Nigel Price**. I think the 30° threshold is quite arbitrary.

**Stefano Negrini**. In reality in the literature the 30° threshold has been reported as important for low back pain
