# Supplementary material for: Recommendations for research studies on treatment of idiopathic scoliosis: Consensus 2014 between SOSORT and SRS non–operative management committee
Source: Scoliosis. 2015 Mar 7;10:8. doi: 10.1186/s13013-014-0025-4 (PMC4360938; doi:10.1186/s13013-014-0025-4)
Supplement: Additional file 7: — Recommendations version 1. [file 13013_2014_25_MOESM7_ESM.doc]

# All studies on non-operative approaches

1. We recommend that new non-operative approaches for all ages and all spinal deformities are continuously explored
2. We recommend that new indications and contraindications for non-operative approaches are continuously explored
3. We recommend that advantages and disadvantages for non-operative approaches are continuously explored
4. We recommend to systematically report radiographic and Quality of Life results of non-operative approaches
5. We recommend that radiographic results are presented in terms of number of patients improved (5° or more), unchanged (+/-4°) and progressed (5° or more)
6. We recommend that radiographic results are presented also split in tables according to Cobb degrees at start of treatment (group of 5° Cobb) and bone age (Risser staging and triradiate cartilage ossification), like the following one:

|  | Infantile | | | Early Onset | | Age 10 and Risser 0 | | Risser 1-5 | | | | |
| --- | --- | --- | --- | --- | --- | --- | --- | --- | --- | --- | --- | --- |
|  | 0 | 1 | 2 | 3-6 | 7-9 | Triradiate open | Triradiate closed | 1 | 2 | 3 | 4 | 5 |
| 20-24 |  |  |  |  |  |  |  |  |  |  |  |  |
| 25-29 |  |  |  |  |  |  |  |  |  |  |  |  |
| 30-34 |  |  |  |  |  |  |  |  |  |  |  |  |
| 35-39 |  |  |  |  |  |  |  |  |  |  |  |  |
| 40-44 |  |  |  |  |  |  |  |  |  |  |  |  |

1. We recommend that standardised and validated questionnaires are used to report Quality of Life results
2. We recommend that patients are split into two groups: previously treated and not treated.
3. We recommend not to consider as a previous treatments any approach with noproves of efficacy in the literature (until now: sport activities, manual treatments general not scoliosis-specific exercises treatments).
4. We recommend to include compliance data, possibly obtained through objective means, and split results according to compliance
5. In the introduction of a new brace / non-operative approach / technique, we recommend that the following research steps are followed during growth:

| **Type of result** | **Data analysed** |
| --- | --- |
| Very short term (only for bracing) | immediate in-brace |
| Short term | 4-6 months of bracing |
| Medium term | Risser 3(European Risser 2) |
| End of treatment | at brace discontinuation |
| Final results at the end of growth | At least 1 year after brace discontinuation AND  Risser 5 and/or ringapophysis closed |
| Follow-ups | To be calculated from final results |

1. In the introduction of a new brace / non-operative approach / technique, we recommend that the following grading of evidence is followed:

| **Level of evidence** | **Type of study** |
| --- | --- |
| A | Retrospective not controlled cohort |
| B | Prospective not controlled cohort |
| C | Retrospective controlled cohort |
| D | Prospective not controlled cohort |
| E | If possible: randomised controlled trial |

1. In the introduction of a new brace, we recommend to focus on the indications proposed by the SRS ^1^
2. In presenting results on bracing, we recommend to answer to the questionnaire in Appendix of the SOSORT Guidelines for Management of braced patients^2^ to understand how team managed patients
3. In presenting results on bracing, we recommend to split results according to the dosage of bracing in terms of impact on patients life, as follows:

| **Definition** | **Nighttime** | **Home-time** | **Half daytime** | **Full time** | **Total time** |
| --- | --- | --- | --- | --- | --- |
| Hours of bracing | 0-10 | 11-14 | 15-18 | 19-21 | 22-24 |

1. At this stage of research on other non-operative approaches during growth, we strongly recommend to present radiographic results (mandatory).

# References

**1.** Richards BS, Bernstein RM, D'Amato CR, Thompson GH. Standardization of criteria for adolescent idiopathic scoliosis brace studies: SRS Committee on Bracing and Nonoperative Management. *Spine* 2005;30:2068-75; discussion 2076-7.

**2.** Negrini S, Grivas TB, Kotwicki T, Rigo M, Zaina F. Guidelines on "Standards of management of idiopathic scoliosis with corrective braces in everyday clinics and in clinical research": SOSORT Consensus 2008. *Scoliosis* 2009;4:2.
