# Supplementary material for: Recommendations for research studies on treatment of idiopathic scoliosis: Consensus 2014 between SOSORT and SRS non–operative management committee
Source: Scoliosis. 2015 Mar 7;10:8. doi: 10.1186/s13013-014-0025-4 (PMC4360938; doi:10.1186/s13013-014-0025-4)
Supplement: Additional file 9: — Recommendations version 2. [file 13013_2014_25_MOESM9_ESM.doc]

# First SOSORT – SRS Consensus 2014

# Recommendations for research studies on non-operative treatment of Idiopathic Scoliosis

1. We recommend that **new** non-operative approaches for all ages and all spinal deformities are continuously explored
2. We recommend that **indications and contraindications** for non-operative approaches are continuously explored
3. We recommend that **strengths and adverse effects** for non-operative approaches are continuously explored
4. We recommend to systematically report radiographic and Quality of Life results of non-operative approaches
5. We recommend that radiographic results are presented in terms of number of patients improved (5° or more), unchanged (+/-4°) and progressed (5° or more)
6. We recommend to report results in terms of number of patients at start and end of treatment exceeding the critical thresholds of 10° (definition of idiopathic scoliosis), [1, 2] 30° (increased possibility of back pain and progression in adulthood) [3-6] and 50° (surgical threshold) [2, 7-9].
7. We recommend the adoption of the SRS-SOSORT “Risser+” staging. This is the result of the confluence between the original US Risser staging, and the so-called European version of Risser staging as modified by Stagnara [10-12]. It has been added also the tryradiate cartilage fusion, that has been shown to be an important and prognostic subdivision of Risser staging 0.

| **SOSORT-SRS**  **“Risser+” staging** | **Tryradiate cartilage ossification** | **US**  **Risser staging** | **European**  **Risser staging** |
| --- | --- | --- | --- |
| 0a | No | 0 | 0 |
| 0b | Yes | 0 | 0 |
| 1  0-25% coverage |  | 1  0-25% coverage | 1  initial ossification |
| 2  25-50% coverage |  | 2  25-50% coverage | 2  partial coverage |
| 3a  50-75% coverage |  | 3  50-75% coverage | 2  partial coverage |
| 3b  75-100% coverage |  | 4  75-100% coverage | 3  complete coverage |
| 4  start of fusion |  |  | 4  start of fusion |
| 5  complete fusion |  | 5  complete fusion | 5  complete fusion |

1. We recommend that radiographic results are presented also split in tables according to Cobb degrees at start of treatment (group of 5° Cobb) and bone age (Risser+ staging), like the following one:

|  | **Early Onset** | | | | | **Juvenile** | **Adolescent** | | | | | | |
| --- | --- | --- | --- | --- | --- | --- | --- | --- | --- | --- | --- | --- | --- |
| *Age at start of treatment* | *0* | *1* | *2* | *3* | *4-5* | *6-9* | *10 or more* | | | | | | |
| *Risser+ staging* |  |  |  |  |  |  | *0a* | *0b* | *1* | *2* | *3a* | *3b* | *4* |
| Below 10° |  |  |  |  |  |  |  |  |  |  |  |  |  |
| 11-15° |  |  |  |  |  |  |  |  |  |  |  |  |  |
| 15-19° |  |  |  |  |  |  |  |  |  |  |  |  |  |
| 20-24° |  |  |  |  |  |  |  |  |  |  |  |  |  |
| 25-29° |  |  |  |  |  |  |  |  |  |  |  |  |  |
| 30-34° |  |  |  |  |  |  |  |  |  |  |  |  |  |
| 35-39° |  |  |  |  |  |  |  |  |  |  |  |  |  |
| 40-44° |  |  |  |  |  |  |  |  |  |  |  |  |  |
| 45-49° |  |  |  |  |  |  |  |  |  |  |  |  |  |
| 50° or more |  |  |  |  |  |  |  |  |  |  |  |  |  |

1. We recommend that standardised and validated questionnaires are used to report Quality of Life results
2. We recommend that patients are split into two groups: previously treated and not treated.
3. We recommend not to consider as a previous treatments any approach without proof of efficacy in the literature
4. We recommend to include compliance data, possibly obtained through objective means, and split results according to compliance
5. In the introduction of a new brace / non-operative approach / technique, we recommend that the following research steps are followed during growth:

| **Type of result** | **Data analysed** |
| --- | --- |
| Very short term (only for bracing) | immediate in-brace |
| Short term | 4-6 months of bracing |
| Medium term | Risser 3(European Risser 2) |
| End of treatment | at brace discontinuation |
| Final results at the end of growth | At least 1 year after brace discontinuation AND  Risser 5 and/or ringapophysis closed |
| Follow-ups | To be calculated from final results |

1. In the introduction of a new brace / non-operative approach / technique, we recommend that the following level of evidence is followed

| **Level of evidence** | **Type of study** |
| --- | --- |
| I | High quality randomized trial  Prospective study |
| II | Lesser quality RCT  prospective comparative study  retrospective study  untreated controls from an RCT  lesser quality prospective study |
| III | Case control study  retrospective comparative study |
| IV | Case series |
| V | Expert opinion |

1. In the introduction of a new brace, we recommend to focus on the indications proposed by the SRS [13]
2. In presenting results on bracing, we recommend to answer to the questionnaire in Appendix of the SOSORT Guidelines for Management of braced patients[14] to understand how team managed patients
3. In presenting results on bracing, we recommend to split results according to the dosage of bracing in terms of impact on patients life, as follows:

| **Definition** | **Nighttime** | **Home-time** | **Half daytime** | **Full time** | **Total time** |
| --- | --- | --- | --- | --- | --- |
| Hours of bracing | 0-10 | 11-14 | 15-18 | 19-21 | 22-24 |

1. At this stage of research on non-operative approaches during growth other than bracing, we strongly recommend to present radiographic results (mandatory).

# References

1. Negrini S, Aulisa AG, Aulisa L, Circo AB, de Mauroy JC, Durmala J, Grivas TB, Knott P, Kotwicki T, Maruyama T *et al*: **2011 SOSORT guidelines: Orthopaedic and Rehabilitation treatment of idiopathic scoliosis during growth**. *Scoliosis* 2012, **7**(1):3.

2. Hresko MT: **Clinical practice. Idiopathic scoliosis in adolescents**. *N Engl J Med* 2013, **368**(9):834-841.

3. Negrini S, Grivas TB, Kotwicki T, Maruyama T, Rigo M, Weiss HR: **Why do we treat adolescent idiopathic scoliosis? What we want to obtain and to avoid for our patients. SOSORT 2005 Consensus paper**. *Scoliosis* 2006, **1**:4.

4. Mayo NE, Goldberg MS, Poitras B, Scott S, Hanley J: **The Ste-Justine Adolescent Idiopathic Scoliosis Cohort Study. Part III: Back pain**. *Spine* 1994, **19**(14):1573-1581.

5. Weinstein SL, Dolan LA, Spratt KF, Peterson KK, Spoonamore MJ, Ponseti IV: **Health and function of patients with untreated idiopathic scoliosis: a 50-year natural history study**. *Jama* 2003, **289**(5):559-567.

6. Weinstein SL, Ponseti IV: **Curve progression in idiopathic scoliosis**. *J Bone Joint Surg Am* 1983, **65**(4):447-455.

7. Weinstein SL, Dolan LA, Wright JG, Dobbs MB: **Effects of bracing in adolescents with idiopathic scoliosis**. *N Engl J Med* 2013, **369**(16):1512-1521.

8. Weinstein SL, Dolan LA, Wright JG, Dobbs MB: **Design of the Bracing in Adolescent Idiopathic Scoliosis Trial (BrAIST)**. *Spine (Phila Pa 1976)* 2013, **38**(21):1832-1841.

9. Weinstein SL, Dolan LA, Cheng JC, Danielsson A, Morcuende JA: **Adolescent idiopathic scoliosis**. *Lancet* 2008, **371**(9623):1527-1537.

10. Stagnara P: **Les deformations du rachis**. Paris: Masson; 1985.

11. Kotwicki T: **Improved accuracy in Risser sign grading with lateral spinal radiography**. *Eur Spine J* 2008, **17**(12):1676-1685.

12. Nault ML, Parent S, Phan P, Roy-Beaudry M, Labelle H, Rivard M: **A modified Risser grading system predicts the curve acceleration phase of female adolescent idiopathic scoliosis**. *J Bone Joint Surg Am* 2010, **92**(5):1073-1081.

13. Richards BS, Bernstein RM, D'Amato CR, Thompson GH: **Standardization of criteria for adolescent idiopathic scoliosis brace studies: SRS Committee on Bracing and Nonoperative Management**. *Spine* 2005, **30**(18):2068-2075; discussion 2076-2067.

14. Negrini S, Grivas TB, Kotwicki T, Rigo M, Zaina F: **Guidelines on "Standards of management of idiopathic scoliosis with corrective braces in everyday clinics and in clinical research": SOSORT Consensus 2008**. *Scoliosis* 2009, **4**(1):2.
