# Supplementary material for: Recommendations for research studies on treatment of idiopathic scoliosis: Consensus 2014 between SOSORT and SRS non–operative management committee
Source: Scoliosis. 2015 Mar 7;10:8. doi: 10.1186/s13013-014-0025-4 (PMC4360938; doi:10.1186/s13013-014-0025-4)
Supplement: Additional file 10: — Discussion version 2. [file 13013_2014_25_MOESM10_ESM.doc]

# Discussions opened

Name and Family name ____________________________________________________________

Position: <> SRS Non Operative Committee <> SOSORT Board

## Definition of the object studied: “non-operative” or “conservative” or “non-surgical” or “medical” (title and throughout the recommendations and paper)

**Tim Hresko.** As mentioned elsewhere, clear definitions of terms is mandatory. “non-operative” is a very explicit term.. “Conservative” is a value judgment , subjective term. Many people, including my mentor Dr John Hall, would say “conservative to what”. Bracing is very radical , not conservative, to some of my patients. I think the term conservative is not precise.

2^nd^ point: Miniamlly invasive surgery is coming. Some would call MIS conservative relative to spinal fusion—I may be one of those persons. Very soon, MIS surgery will be compared to bracing and other non operative treatments. The same inclusion criteria we develop should be used by the MIS tethering proponents . So , I would propose that we consider terms such as “non- fusion”, “motion preservation” “ Functional treatment” though out the process. We then strictly define the terms and the rational for the terms.

**Stefano Negrini**. My preferred term, according to the name of our Society, would be “orthopedic and rehabilitation treatment”, since either “conservative” and “non-operative” define by exclusion. To me, a definition should define what is done, and not “everything apart another thing” (I mean, everything apart surgery). In the main time, the old term “conservative” include all what is today in the field of orthopedic medicine (not-surgical) but also “rehabilitation medicine”. That’s the reason for my proposal.

**Joe O’Brien**. I do envision a day when a minimally invasive apical surgical procedure may be deemed to be the optimal treatment for spinal deformity and even “conservative” depending on the defined judgement criteria. In this regard, “Non Operative” is a clearer and generally a more widely accepted term to describe the forms of treatment we are addressing in this current Consensus. Contrary to your point however I believe this term is too inclusionary rather than exclusionary and further definition and classifications may be helpful. To SOSORT members “Non Operative” primarily refers to various Bracing and Scoliosis Specific Exercises (schools), while outside reference may be towards a far broader range with different meaning – ie,

Spine (Phila Pa 1976). 2010 Mar 1;35(5):578-82. doi: 10.1097/BRS.0b013e3181b0f2f8. The costs and benefits of nonoperative management for adult scoliosis.

Glassman SD1, Carreon LY, Shaffrey CI, Polly DW, Ondra SL, Berven SH, Bridwell KH. Duration of use and frequency of visits were collected for 8 specific treatment methods: medication, physical therapy, exercise, injections/blocks, chiropractic care, pain management, bracing, and bed rest.

Other studies refer to Acupuncture, Yoga, Tai Chi, and a host of other medical, alternative, or complentary active or passive treatments. I think this is confusing and we may be better served in the future by developing sub class categories to differentiate these forms of treatment rather than rely (and be judged) on one broad term.

**Stefano Negrini**. After reflection, starting from the main idea that defining by exclusion is not correct, and that also in our basic medical studies we use the categories “surgical” and “medical”, I propose “medical approach” instead of “non-operative” or “conservative”

**Patrick Knott**. I would INCLUDE rehabilitation exercises and bracing for now, and then ADD more treatments to this list as we wish to study them. This way we exclude other treatments being lumped in with them (eg. chiropractic, acupuncture, etc.)

**Tomasz Kotwicki**. non-surgical treatment

**Luke Stikeleather**. SRS SOSORT initiative, we are attempting to establish a well defined inclusion criteria that will be universally accepted.” Non –operative” leaves little room for confusion.

**Fabio Zaina**. Rehabilitation treatment for scoliosis

Your considerations: _______________________________________________________________

________________________________________________________________________________

________________________________________________________________________________

# Thresholds of scoliosis (recommendation 6)

**Nigel Price**. I think the 30° threshold is quite arbitrary.

**Stefano Negrini**. In reality in the literature the 30° threshold has been reported as important for low back pain

**Channing Tassone**. Agree with concern of 30 degree classification. Want to avoid message that the 30 – 50 degree curves will have issues as adults

**Toru Maruyama**. 10 degrees instead of 11 degrees

**Joe O'Brien**. "J Bone Joint Surg Am. 1983 Apr;65(4):447-55. Curve progression in idiopathic scoliosis. Weinstein SL, Ponseti IV. “In general, curves that were less than 30 degrees at skeletal maturity tended not to progress regardless of curve pattern.”

**Luke Stikeleather**. Are you suggesting identifying these 3 thresholds as significant to treatment decision making or prognosis or????

Your considerations: _______________________________________________________________

________________________________________________________________________________

________________________________________________________________________________

## Risser sign and other signs of bone age (recommendation 7)

**Nigel Price.** Future studies need to break out Risser 0, triradiate cartilge open ( very high risk) from the Risser 2, also Risser classification of Risser 2 is very unreliable and here I agree with European partial coverage.

**Stefano Negrini** I agree on this point: in fact we could introduce this concept in the text. Nevertheless, we run the risk to enter a little too much into the details if we considered that the aim is to offer a general scheme.

**Nigel Price**. This may be an opportunity to start using elbow ulnar apophysis and hand age criteria.

**Stefano Negrini**. The same as above for triradiate cartilage. The other problem here is that elbow ulnar apophysis, as far as I know, has been proposed by Dimeglio but it is not validated. Am I wrong ? Moreover, it is not visible in most of cases in the regular x-rays, while , in the new proposed positioning of the hands for the EOS system it is not visible by definition.

**Tim Hresko**. “European Risser 4” is probably a radiological artefact due to superposition of the secondary ossification centre of the iliac crest with the shadow of the posterior part of the iliac bone (Eur Spine J 2008; 17: 1676-85). For me, no need to promote this parameter. We should better stay simple: Risser zero, Risser negative (0,1,2), Risser positive (3,4,5) "

**Channing Tassone.** It is better to clearly name the stages of Risser: SRS and SOSORT must make an agreement on the RISSER stage (Instead of the European or American Risser sign we will have a universally agreed Risser sign (The SOSORT- SRS Risser sign)

**Theo Grivas.** It is better to clearly name the stages of Risser: SRS and SOSORT must make an agreement on the RISSER stage (Instead of the European or American Risser sign we will have a universally agreed Risser sign (The SOSORT- SRS Risser sign)

**Luke Stikeleather.** Is it too big a task to create a universally accepted standard? Now that SRS /SOSORT are international organizations can we propose discussion on this topic?

Your considerations: _______________________________________________________________

________________________________________________________________________________

________________________________________________________________________________

## Classification according to age (recommendation 8)

**Nigel Price.** Stefano-is this splitting of age groups a SOSORT consensus? I am familiar with the concept of early onset being less than 5 years-a concept the the Early Onset Scoliosis Group considers important-are the groups really heterogeneous?

**Stefano Negrini.** We re-introduced this classification in the SOSORT Guidelines. In reality, a classification should help for prognosis and treatment. In conservative, we face aggressive curves due to rapid growth: this is true until age 4, when growth slows down. In the past this classification was created, we could come out with a new one, but really in non-operative not splitting these groups could be a limitation. Nevertheless, this should be a point to be thoroughly discussed and we must maintain this point open to discussion. Tim proposed to join Infantile and Juvenile 1: the main difference here, to me, is that in the first year of age you can still have the self-resolving, that is not true afterwards. Really, I would like to know the opinion of the others as well before chosing.

3Tim: why not include Juvenile 1 with the infantile? To me, there is a big difference since in the first year of age you can still have the self-resolving, that is not true afterwards. Really, I would like to know the opinion of the others as well before chosing.

**Tim Hresko.** Early onset scoliosis is avery common term used by SRS members and of great interest due to concern for chest growth, pulmonary function when fusion is perform in less than age 8. Can we use the term EOS but then subclassifiy it into EOS- infantile and EOS- juvenile 1( under age 7, too early for exercise approaches) and juvenile 2( old enough for exercise)? Again, to be discussed.

**Channing Tassone**. I do not find this very useful and I find it confusing, therefore the rest of the questionnaire must be constructed according to a more simple approach on the issue.

**Theo Grivas**. I do not find this very useful and I find it confusing, therefore the rest of the questionnaire must be constructed according to a more simple approach on the issue.

**Tim Hresko**. It would be best to be in agreement with the established EOS study groups that define EOS < age 5. I think there are too many sub groups. I would suggest EOS 0-5; Juvenile 1- age 5-7; juvenile 2- age 8-9; Adolescent > 10yr triradiate cartilage open and Risser 0; Risser 1-2; Risser 3-4-5; adult to menopause; all the rest the same but change term “flexed posture” to unbalanced sagittal posture or something else

**Tomasz Kotwicki**. The proposal for Juvenile 1, 2, and 3 is right, however too complex for researchers to be followed. And you never knows exactly when the curve appeared, you only know when it was noticed – thus, such division of Juveniles refers more to quality of health care in early detection of scoliosis than to natural history of deforming spine.

**Luke Stikeleather**. The term” early onset” seem to be well established ...but too broad. I like the idea of categorizing by age, as treatment may be very different and specific to these age groups. The SRS website still uses the infantile, juvenile, adolescent description under early onset. Can we use EOS and patient age with it? For example EOS 1 or EOS 7 or a range EOS 5-7.

Your considerations: _______________________________________________________________

________________________________________________________________________________

________________________________________________________________________________

# Including in research purposes also curve below 11° (recommendation 8)

**Channing Tassone**. Curve >11 degrees to avoid unnecessary treatment

**Tim Hresko**. There should be radiographic curve greater than 10 degree

**Patrick Knott**. I am concerned that we may continue to define smaller and smaller deformities for research groups. These lead everyone to believe that these curves need treatment, when they may not.

**Tomasz Kotwicki**. Minimal curves analyzed in separate category

**Luke Stikeleather**. What is the incidence of this? And do we need more discussion on it?

Your considerations: _______________________________________________________________

________________________________________________________________________________

________________________________________________________________________________
