# Supplementary material for: Recommendations for research studies on treatment of idiopathic scoliosis: Consensus 2014 between SOSORT and SRS non–operative management committee
Source: Scoliosis. 2015 Mar 7;10:8. doi: 10.1186/s13013-014-0025-4 (PMC4360938; doi:10.1186/s13013-014-0025-4)
Supplement: Additional file 11: — Questionnaire 2. [file 13013_2014_25_MOESM11_ESM.doc]

Dear colleague: please proceed in this way:

1. Read the document on the Recommendations
2. Read the discussions opened and give your statements on them, if you want: these will be continued during the next SOSORT Meeting, but you need to know these discussions to answer properly to the Questionnaire
3. Answer to the questionnaire: cross the appropriate square and/or number and eventually add your suggestions
4. Send your answers by the DEADLINE of April 13^th^ so to give the time for collecting them and organizing the Consensus Session in due time

# Consensus questionnaire

Name and Family name ____________________________________________________________

Position: <> SRS Non Operative Committee <> SOSORT Board

# First SOSORT – SRS Consensus 2014

# Recommendations for research studies on non-operative treatment of Idiopathic Scoliosis

Do you agree with the title ? Yes No

Suggestions: _____________________________________________________________________

________________________________________________________________________________

________________________________________________________________________________

1. We recommend that **new** non-operative approaches for all ages and all spinal deformities are continuously explored

Do you agree with this recommendation ? <> Yes <> Yes with suggestions <> No

Degree of importance: 0 No 1 Low 2 Medium 3 High 4 Maximum

Suggestions: _____________________________________________________________________

________________________________________________________________________________

________________________________________________________________________________

1. We recommend that **indications and contraindications** for non-operative approaches are continuously explored

Do you agree with this recommendation ? <> Yes <> Yes with suggestions <> No

Degree of importance: 0 No 1 Low 2 Medium 3 High 4 Maximum

Suggestions: _____________________________________________________________________

________________________________________________________________________________

________________________________________________________________________________

1. We recommend that **strengths and adverse effects** for non-operative approaches are continuously explored

Do you agree with this recommendation ? <> Yes <> Yes with suggestions <> No

Degree of importance: 0 No 1 Low 2 Medium 3 High 4 Maximum

Suggestions: _____________________________________________________________________

________________________________________________________________________________

________________________________________________________________________________

1. We recommend to systematically report radiographic and Quality of Life results of non-operative approaches

Do you agree with this recommendation ? <> Yes <> Yes with suggestions <> No

Degree of importance: 0 No 1 Low 2 Medium 3 High 4 Maximum

Suggestions: _____________________________________________________________________

________________________________________________________________________________

________________________________________________________________________________

1. We recommend that radiographic results are presented in terms of number of patients improved (5° or more), unchanged (+/-4°) and progressed (5° or more)

Do you agree with this recommendation ? <> Yes <> Yes with suggestions <> No

Degree of importance: 0 No 1 Low 2 Medium 3 High 4 Maximum

Suggestions: _____________________________________________________________________

________________________________________________________________________________

________________________________________________________________________________

1. We recommend to report results in terms of number of patients at start and end of treatment exceeding the critical thresholds of 10° (definition of idiopathic scoliosis) [1, 2], 30° (increased possibility of back pain and progression in adulthood) [3-6] and 50° (surgical threshold) [2, 7-9].

Do you agree with this recommendation ? <> Yes <> Yes with suggestions <> No

Degree of importance: 0 No 1 Low 2 Medium 3 High 4 Maximum

Suggestions: _____________________________________________________________________

________________________________________________________________________________

________________________________________________________________________________

1. We recommend the adoption of the SRS-SOSORT “Risser+” staging. This is the result of the confluence between the original US Risser staging, and the so-called European version of Risser staging as modified by Stagnara [10-12]. It has been added also the tryradiate cartilage fusion, that has been shown to be an important and prognostic subdivision of Risser staging 0.

| **SOSORT-SRS**  **“Risser+” staging** | **Tryradiate cartilage ossification** | **US**  **Risser staging** | **European**  **Risser staging** |
| --- | --- | --- | --- |
| 0a | No | 0 | 0 |
| 0b | Yes | 0 | 0 |
| 1  0-25% coverage |  | 1  0-25% coverage | 1  initial ossification |
| 2  25-50% coverage |  | 2  25-50% coverage | 2  partial coverage |
| 3a  50-75% coverage |  | 3  50-75% coverage | 2  partial coverage |
| 3b  75-100% coverage |  | 4  75-100% coverage | 3  complete coverage |
| 4  start of fusion |  |  | 4  start of fusion |
| 5  complete fusion |  | 5  complete fusion | 5  complete fusion |

Do you agree with this recommendation ? <> Yes <> Yes with suggestions <> No

Degree of importance: 0 No 1 Low 2 Medium 3 High 4 Maximum

Suggestions: _____________________________________________________________________

________________________________________________________________________________

________________________________________________________________________________

1. We recommend that radiographic results are presented also split in tables according to Cobb degrees at start of treatment (group of 5° Cobb) and bone age (Risser+ staging), like the following one:

|  | **Early Onset** | | | | | **Juvenile** | **Adolescent** | | | | | | |
| --- | --- | --- | --- | --- | --- | --- | --- | --- | --- | --- | --- | --- | --- |
| *Age at start of treatment* | *0* | *1* | *2* | *3* | *4-5* | *6-9* | *10 or more* | | | | | | |
| *Risser+ staging* |  |  |  |  |  |  | *0a* | *0b* | *1* | *2* | *3a* | *3b* | *4* |
| Below 10° |  |  |  |  |  |  |  |  |  |  |  |  |  |
| 11-15° |  |  |  |  |  |  |  |  |  |  |  |  |  |
| 15-19° |  |  |  |  |  |  |  |  |  |  |  |  |  |
| 20-24° |  |  |  |  |  |  |  |  |  |  |  |  |  |
| 25-29° |  |  |  |  |  |  |  |  |  |  |  |  |  |
| 30-34° |  |  |  |  |  |  |  |  |  |  |  |  |  |
| 35-39° |  |  |  |  |  |  |  |  |  |  |  |  |  |
| 40-44° |  |  |  |  |  |  |  |  |  |  |  |  |  |
| 45-49° |  |  |  |  |  |  |  |  |  |  |  |  |  |
| 50° or more |  |  |  |  |  |  |  |  |  |  |  |  |  |

Do you agree with this recommendation ? <> Yes <> Yes with suggestions <> No

Degree of importance: 0 No 1 Low 2 Medium 3 High 4 Maximum

Suggestions: _____________________________________________________________________

________________________________________________________________________________

________________________________________________________________________________

1. We recommend that standardised and validated questionnaires are used to report Quality of Life results

Do you agree with this recommendation ? <> Yes <> Yes with suggestions <> No

Degree of importance: 0 No 1 Low 2 Medium 3 High 4 Maximum

Suggestions: _____________________________________________________________________

________________________________________________________________________________

________________________________________________________________________________

1. We recommend that patients are split into two groups: previously treated and not treated.

Do you agree with this recommendation ? <> Yes <> Yes with suggestions <> No

Degree of importance: 0 No 1 Low 2 Medium 3 High 4 Maximum

Suggestions: _____________________________________________________________________

________________________________________________________________________________

________________________________________________________________________________

1. We recommend not to consider as a previous treatments any approach without proof of efficacy in the literature

Do you agree with this recommendation ? <> Yes <> Yes with suggestions <> No

Degree of importance: 0 No 1 Low 2 Medium 3 High 4 Maximum

Suggestions: _____________________________________________________________________

________________________________________________________________________________

________________________________________________________________________________

1. We recommend to include compliance data, possibly obtained through objective means, and split results according to compliance

Do you agree with this recommendation ? <> Yes <> Yes with suggestions <> No

Degree of importance: 0 No 1 Low 2 Medium 3 High 4 Maximum

Suggestions: _____________________________________________________________________

________________________________________________________________________________

________________________________________________________________________________

1. In the introduction of a new brace / non-operative approach / technique, we recommend that the following research steps are followed during growth:

| **Type of result** | **Data analysed** |
| --- | --- |
| Very short term (only for bracing) | immediate in-brace |
| Short term | 4-6 months of bracing |
| Medium term | Risser 3(European Risser 2) |
| End of treatment | at brace discontinuation |
| Final results at the end of growth | At least 1 year after brace discontinuation AND  Risser 5 and/or ringapophysis closed |
| Follow-ups | To be calculated from final results |

Do you agree with this recommendation ? <> Yes <> Yes with suggestions <> No

Degree of importance: 0 No 1 Low 2 Medium 3 High 4 Maximum

Suggestions: _____________________________________________________________________

________________________________________________________________________________

________________________________________________________________________________

1. In the introduction of a new brace / non-operative approach / technique, we recommend that the following level of evidence is followed

| **Level of evidence** | **Type of study** |
| --- | --- |
| I | High quality randomized trial  Prospective study |
| II | Lesser quality RCT  prospective comparative study  retrospective study  untreated controls from an RCT  lesser quality prospective study |
| III | Case control study  retrospective comparative study |
| IV | Case series |
| V | Expert opinion |

Do you agree with this recommendation ? <> Yes <> Yes with suggestions <> No

Degree of importance: 0 No 1 Low 2 Medium 3 High 4 Maximum

Suggestions: _____________________________________________________________________

________________________________________________________________________________

________________________________________________________________________________

1. In the introduction of a new brace, we recommend to focus on the indications proposed by the SRS [13]

Do you agree with this recommendation ? <> Yes <> Yes with suggestions <> No

Degree of importance: 0 No 1 Low 2 Medium 3 High 4 Maximum

Suggestions: _____________________________________________________________________

________________________________________________________________________________

________________________________________________________________________________

1. In presenting results on bracing, we recommend to answer to the questionnaire in Appendix of the SOSORT Guidelines for Management of braced patients[14] to understand how team managed patients

Do you agree with this recommendation ? <> Yes <> Yes with suggestions <> No

Degree of importance: 0 No 1 Low 2 Medium 3 High 4 Maximum

Suggestions: _____________________________________________________________________

________________________________________________________________________________

________________________________________________________________________________

1. In presenting results on bracing, we recommend to split results according to the dosage of bracing in terms of impact on patients life, as follows:

| **Definition** | **Nighttime** | **Home-time** | **Half daytime** | **Full time** | **Total time** |
| --- | --- | --- | --- | --- | --- |
| Hours of bracing | 0-10 | 11-14 | 15-18 | 19-21 | 22-24 |

Do you agree with this recommendation ? <> Yes <> Yes with suggestions <> No

Degree of importance: 0 No 1 Low 2 Medium 3 High 4 Maximum

Suggestions: _____________________________________________________________________

________________________________________________________________________________

________________________________________________________________________________

1. At this stage of research on non-operative approaches during growth other than bracing, we strongly recommend to present radiographic results (mandatory).

Do you agree with this recommendation ? <> Yes <> Yes with suggestions <> No

Degree of importance: 0 No 1 Low 2 Medium 3 High 4 Maximum

Suggestions: _____________________________________________________________________

________________________________________________________________________________

________________________________________________________________________________

# References

1. Negrini S, Aulisa AG, Aulisa L, Circo AB, de Mauroy JC, Durmala J, Grivas TB, Knott P, Kotwicki T, Maruyama T *et al*: **2011 SOSORT guidelines: Orthopaedic and Rehabilitation treatment of idiopathic scoliosis during growth**. *Scoliosis* 2012, **7**(1):3.

2. Hresko MT: **Clinical practice. Idiopathic scoliosis in adolescents**. *N Engl J Med* 2013, **368**(9):834-841.

3. Negrini S, Grivas TB, Kotwicki T, Maruyama T, Rigo M, Weiss HR: **Why do we treat adolescent idiopathic scoliosis? What we want to obtain and to avoid for our patients. SOSORT 2005 Consensus paper**. *Scoliosis* 2006, **1**:4.

4. Mayo NE, Goldberg MS, Poitras B, Scott S, Hanley J: **The Ste-Justine Adolescent Idiopathic Scoliosis Cohort Study. Part III: Back pain**. *Spine* 1994, **19**(14):1573-1581.

5. Weinstein SL, Dolan LA, Spratt KF, Peterson KK, Spoonamore MJ, Ponseti IV: **Health and function of patients with untreated idiopathic scoliosis: a 50-year natural history study**. *Jama* 2003, **289**(5):559-567.

6. Weinstein SL, Ponseti IV: **Curve progression in idiopathic scoliosis**. *J Bone Joint Surg Am* 1983, **65**(4):447-455.

7. Weinstein SL, Dolan LA, Wright JG, Dobbs MB: **Effects of bracing in adolescents with idiopathic scoliosis**. *N Engl J Med* 2013, **369**(16):1512-1521.

8. Weinstein SL, Dolan LA, Wright JG, Dobbs MB: **Design of the Bracing in Adolescent Idiopathic Scoliosis Trial (BrAIST)**. *Spine (Phila Pa 1976)* 2013, **38**(21):1832-1841.

9. Weinstein SL, Dolan LA, Cheng JC, Danielsson A, Morcuende JA: **Adolescent idiopathic scoliosis**. *Lancet* 2008, **371**(9623):1527-1537.

10. Stagnara P: **Les deformations du rachis**. Paris: Masson; 1985.

11. Kotwicki T: **Improved accuracy in Risser sign grading with lateral spinal radiography**. *Eur Spine J* 2008, **17**(12):1676-1685.

12. Nault ML, Parent S, Phan P, Roy-Beaudry M, Labelle H, Rivard M: **A modified Risser grading system predicts the curve acceleration phase of female adolescent idiopathic scoliosis**. *J Bone Joint Surg Am* 2010, **92**(5):1073-1081.

13. Richards BS, Bernstein RM, D'Amato CR, Thompson GH: **Standardization of criteria for adolescent idiopathic scoliosis brace studies: SRS Committee on Bracing and Nonoperative Management**. *Spine* 2005, **30**(18):2068-2075; discussion 2076-2067.

14. Negrini S, Grivas TB, Kotwicki T, Rigo M, Zaina F: **Guidelines on "Standards of management of idiopathic scoliosis with corrective braces in everyday clinics and in clinical research": SOSORT Consensus 2008**. *Scoliosis* 2009, **4**(1):2.
