# Supplementary material for: Recommendations for research studies on treatment of idiopathic scoliosis: Consensus 2014 between SOSORT and SRS non–operative management committee
Source: Scoliosis. 2015 Mar 7;10:8. doi: 10.1186/s13013-014-0025-4 (PMC4360938; doi:10.1186/s13013-014-0025-4)
Supplement: Additional file 12: — Questionnaire 3. [file 13013_2014_25_MOESM12_ESM.pdf]

First SOSORT SRS Joint Consensus 2014

Instructions

Dear colleague,

These recommendations come out after 4 steps of a Delphi procedure to gather the best Consensus on the topic:
1. First set developed and tuned by the joint SOSORT-SRS Commission (Negrini, Hresko, O'Brien, Price)
2. First set voted by the SOSORT Executive Committee and Advisory Board and the SRS Non-Operative Committee (23 people)
3. Second set developed and tuned by joint SOSORT-SRS Commission
4. Second set voted by the SOSORT Boards and the SRS Non-Operative Committee

Now your vote is needed BEFORE the Meeting.

The Consensus procedure will be closed in a specific Consensus Session during the Meeting, and the final paper will be formally approved by the SOSORT and SRS Boards.

This survey will take almost 15 minutes of your time, but there is no need to underline the importance of this FIRST JOINT EFFORT of the two leading clinical and research Societies in the field of scoliosis.

You will have to vote 18 recommendations, and the questions are standardised for each recommendation.

For each Recommendation you will be required to vote
a. agreement (or disagreement) in giving this recommendation (even if the wording could change in the final version that will be voted during the Meeting)
b. grade the importance of the actual recommendation

You can also propose new versions of the Recommendations to be voted during the Meeting.

You will receive by email a document with all the discussion on each recommendation. The final version of this document will include all suggestions you will give during this survey: it will be attached to the final paper. You can read it for completeness, or skip it if you do not have enough time.

PLEASE ANSWER at your earliest convenience.

DEADLINE of April 30th, so to have time to prepare the Session to be held during the Meeting.

Generalities of respondent

*1. What is your first name?

*2. What is your last name?

*3. What is your gender?

- Female
- Male

*4. What is your age?

- 18 to 24
- 25 to 34
- 35 to 44
- 45 to 54
- 55 to 64
- 65 to 74
- 75 or older

*5. Profession

- MD, Orthopedic Surgeon
- MD, Physical and Rehabilitation Medicine
- MD, other
- Physical Therapist
- Orthotist
- PhD
- Other (please specify)

*6. Society of the responder

- ☐ SOSORT Meeting participant
- ☐ SOSORT Member
- ☐ SOSORT Executive Committee
- ☐ SOSORT Advisory Board
- ☐ SRS Non Operative Committee
- ☐ SRS Presidential Line

Title: Recommendations for research studies on non-operative treatment of I...

The discussion focused on the definition "non-operative treatment".
There is until now quite big disagreement.
Your vote now will serve to guide the final decision during the Meeting.
The term that will be chosen in the end will be changed not only in the title but also throughout the document.

(if you want, you can see discussion in the attached document)

***7. Rank the following terms according to your preference (to be used in the title and througout the document)**
Only the first 5 ranked answers will be retained for further analysis
1. Best
5. Worst
From 6 to 12. Eliminated

☐	Bracing and Exercises
☐	Bracing and Physiotherapeutic Scoliosis Specific Exercises (PSSE)
☐	Conservative
☐	Functional
☐	Medical
☐	Non-operative
☐	Non-surgical
☐	Orthopedic and Rehabilitation
☐	Orthopedic and Rehabilitation Medicine
☐	Physical and Rehabilitation Medicine
☐	Rehabilitation
☐	Rehabilitation Medicine

Recommendation 1

We recommend that NEW non-operative approaches for all ages and all spinal deformities are continuously explored

***8. Do you agree in giving this recommendation (eventually in a new version) ?**

- Yes
- Yes with suggestions (to be added in next question)
- No

9. What suggestions do you have for improving this recommendation?

***10. Please, rate the importance of this recommendation**

- 1 - Very Low
- 2 - Low
- 3 - Medium
- 4 - High
- 5 - Very high

11. Do you suggest any other version / wording for this recommendation ?

Recommendation 2

We recommend that INDICATIONS AND CONTRAINDICATIONS for non-operative approaches are continuously explored

*12. Do you agree in giving this recommendation (eventually in a new version) ?

- Yes
- Yes with suggestions (to be added in next question)
- No

13. What suggestions do you have for improving this recommendation?

*14. Please, rate the importance of this recommendation

- 1 - Very Low
- 2 - Low
- 3 - Medium
- 4 - High
- 5 - Very high

15. Do you suggest any other version / wording for this recommendation ?

Recommendation 3

We recommend that STRENGTHS and ADVERSE EFFECTS for non-operative approaches are continuously explored

***16. Do you agree in giving this recommendation (eventually in a new version) ?**

- Yes
- Yes with suggestions (to be added in next question)
- No

17. What suggestions do you have for improving this recommendation?

***18. Please, rate the importance of this recommendation**

- 1 - Very Low
- 2 - Low
- 3 - Medium
- 4 - High
- 5 - Very high

19. Do you suggest any other version / wording for this recommendation ?

Recommendation 4

We recommend to systematically report radiographic and Quality of Life outcomes of non-operative approaches

(if you want, you can see discussion in the attached document)

*20. Do you agree in giving this recommendation (eventually in a new version) ?

- Yes
- Yes with suggestions (to be added in next question)
- No

21. What suggestions do you have for improving this recommendation?

*22. Please, rate the importance of this recommendation

- 1 - Very Low
- 2 - Low
- 3 - Medium
- 4 - High
- 5 - Very high

*23. Please, rank the following different versions of this recommendation

(Comment: According to the Cochrane Institute primary outcomes are those focused on the patient, while secondary outcomes are those focused on biological and instrumental data that predict possible results on the patient but are not immediately a symptom. Accordingly the sentence could be changed We have now two versions to be voted)

	Version 1. We recommend to systematically report radiographic and Quality of Life outcomes of non-operative approaches
	Version 2. We recommend to systematically report the outcomes of non-operative approaches in terms of Quality of Life (primary patient-centered outcomes), and radiographic data (secondary outcomes, predictive of consequences on the patient).
	Version 3. We recommend to systematically report radiographic and Quality of Life outcomes of non-operative approaches. Other possible evaluations (not to be considered as main outcomes) include: Angle of Trunk Rotation (ATR), aesthetic parameters and surface topography.
	Version 4. We recommend to systematically report the outcomes of non-operative approaches in terms of Quality of Life (primary patient-centered outcomes), and radiographic data (secondary outcomes, predictive of consequences on the patient). Other possible evaluations (not to be considered as main outcomes) include: Angle of Trunk Rotation (ATR), aesthetic parameters and surface topography.

24. Do you suggest any other version / wording for this recommendation ?

Recommendation 5

We recommend to report results in terms of number of patients at start and end of treatment exceeding the critical thresholds of 10° (definition of idiopathic scoliosis), 30° (increased possibility of back pain and progression in adulthood) and 50° (surgical threshold)

(references have been inserted in the attached document)

(if you want, you can see discussion in the attached document)

*25. Do you agree in giving this recommendation (eventually in a new version) ?

- Yes
- Yes with suggestions (to be added in next question)
- No

26. What suggestions do you have for improving this recommendation?

*27. Please, rate the importance of this recommendation

- 1 - Very Low
- 2 - Low
- 3 - Medium
- 4 - High
- 5 - Very high

*28. Please, rank the following different versions of this recommendation

	Version 1. We recommend to report results in terms of number of patients at start and end of treatment exceeding the critical thresholds of 10° (definition of idiopathic scoliosis), 30° (increased possibility of back pain and progression in adulthood) and 50° (surgical threshold)
	Version 2. We recommend to report results in the clinically significant terms of number of patients at start and end of treatment exceeding 10°, 30° and 50° Cobb
	Version 3. We recommend to report results in the clinically significant terms of number of patients at start and end of treatment exceeding 10°, 30° and 50° Cobb: epidemiology and actual clinical practice recognises these as risk thresholds for possible future health consequences (back pain and progression in adulthood), even if their importance should be defined case by case in front of single patients
	Version 4. We recommend to report results in terms of number of patients at start and end of treatment exceeding 10°, 30° and 50° Cobb: epidemiology and actual clinical practice has recognised these as meaningful thresholds, even if their importance should be defined case by case in front of single patients
	Version 5. We recommend to report results in terms of number of patients at start and end of treatment exceeding 10°, 30° and 50° Cobb

29. Do you suggest any other version / wording for this recommendation ?

Recommendation 6

We recommend that radiographic results are presented in terms of number of patients improved (6° or more), unchanged (+/-5°) and progressed (6° or more)

(if you want, you can see discussion in the attached document)

*30. Do you agree in giving this recommendation (eventually in a new version) ?

- Yes
- Yes with suggestions (to be added in next question)
- No

31. What suggestions do you have for improving this recommendation?

*32. Please, rate the importance of this recommendation

- 1 - Very Low
- 2 - Low
- 3 - Medium
- 4 - High
- 5 - Very high

*33. Please, rank the following different versions of this recommendation

	Version 1. We recommend that radiographic results are presented in terms of number of patients improved (6° or more), unchanged (+/-5°) and progressed (6° or more)
	Version 2. We recommend to give outcomes mainly in clinically significant terms according to the recommendation 5. Radiographic outcomes, if presented, must be given in terms of number of patients improved (6° or more), unchanged (+/-5°) and progressed (6° or more)
	Version 3. We recommend to give outcomes mainly in clinically significant terms according to the recommendation 5. Radiographic outcomes, if presented, must be given in terms of number of patients improved (6° or more), unchanged (+/-5°) and progressed (6° or more) and not of average and standard deviations

34. Do you suggest any other version / wording for this recommendation ?

Recommendation 7

We recommend the adoption of the SRS-SOSORT “Risser+” staging. This is the result of the confluence between the original US Risser staging, and the so-called European version of Risser staging as modified by Stagnara. It has been added also the tyradiate cartilage fusion, that has been shown to be an important and prognostic subdivision of Risser staging 0

SOSORT-SRS “Risser+” Staging System

Risser+ 0a: US and EU Risser staging 0, without ossification of tyradiate cartilage
Risser+ 0b: US and EU Risser staging 0, with ossification of tyradiate cartilage
Risser+ 1 (0-25% coverage): US and EU Risser staging 1
Risser+ 2 (25-50% coverage): US and EU Risser staging 2
Risser+ 3a (50-75% coverage): US Risser staging 3 and EU Risser staging 2
Risser+ 3b (75-100% coverage): US Risser staging 4 and EU Risser staging 3
Risser+ 4 (start of fusion): US and EU Risser staging 4
Risser+ 5 (complete fusion): US and EU Risser staging 5

(references have been inserted in the attached document)

(if you want, you can see discussion in the attached document)

*35. Do you agree in giving this recommendation (eventually in a new version) ?

- Yes
- Yes with suggestions (to be added in next question)
- No

36. What suggestions do you have for improving this recommendation?

*37. Please, rate the importance of this recommendation

- 1 - Very Low
- 2 - Low
- 3 - Medium
- 4 - High
- 5 - Very high

***38. Please, rank the following different versions of this recommendation**

- Version 1: Risser+ stages: 0a, 0b, 1, 2, 3a, 3b, 4, 5
- Version 2: Risser+ stages: 0a, 0b, 1, 2, 3, 3+, 4, 5
- Version 3: Risser+ stages: 0a, 0b, 1, 2, 3, 4-, 4, 5
- Version 4: Risser+ stages: 0-, 0, 1, 2, 3a, 3b, 4, 5
- Version 5: Risser+ stages: 0-, 0, 1, 2, 3, 3+, 4, 5
- Version 6: Risser+ stages: 0-, 0, 1, 2, 3, 4-, 4, 5
- Version 7: Risser+ stages: 0o, 0c, 1, 2, 3a, 3b, 4, 5 - 0o and 0c refer to tryradiate cartilage open or closed
- Version 8: Risser+ stages: 0o, 0c, 1, 2, 3, 3+, 4, 5
- Version 9: Risser+ stages: 0o, 0c, 1, 2, 3, 4-, 4, 5

39. Do you suggest any other version / wording for this recommendation ?

Recommendation 8

We recommend that radiographic results are presented also split in tables according to Cobb degrees at start of treatment (group of 5° Cobb) and bone age (Risser+ staging), like the following one

Columns:
- Early onset (divided according to age: years 0, 1, 2, 3, 4-5)
- Juvenile (only one age group 6-9)
- Adolescents (divided according to Risser+ staging)

Rows:
- Below 10°
- 10-14°
- 15-19°
- 20-24°
- 25-29°
- 30-34°
- 35-39°
- 40-44°
- 45-49°
- 50° or more

(if you want, you can see discussion in the attached document)

*40. Do you agree in giving this recommendation (eventually in a new version) ?

- Yes
- Yes with suggestions (to be added in next question)
- No

41. What suggestions do you have for improving this recommendation?

*42. Please, rate the importance of this recommendation

- 1 - Very Low
- 2 - Low
- 3 - Medium
- 4 - High
- 5 - Very high

*43. Please, rank the following different versions of this recommendation

- Version 1. Same as above
- Version 2. Same as above, with first row change: instead of Below 10°: Below 10° with a rib hump / lumbar prominence
- Version 3. Columns: as above - Rows: 10° steps (Below 10°, 10-19°, 20-29°, 30-39°, 40-49°, 50° or more)
- Version 4. Columns: as above - Rows: 10° steps. First row change: instead of Below 10°: Below 10° with a rib hump / lumbar prominence

44. Do you suggest any other version / wording for this recommendation ?

Recommendation 9

We recommend that standardised and validated questionnaires are used to report Quality of Life results

(if you want, you can see discussion in the attached document)

*45. Do you agree in giving this recommendation (eventually in a new version) ?

- Yes
- Yes with suggestions (to be added in next question)
- No

46. What suggestions do you have for improving this recommendation?

*47. Please, rate the importance of this recommendation

- 1 - Very Low
- 2 - Low
- 3 - Medium
- 4 - High
- 5 - Very high

48. Do you suggest any other version / wording for this recommendation ?

Recommendation 10

We recommend that patients are split into two groups: previously treated and not treated. We recommend not to consider as a previous treatments any approach without proof of efficacy in the literature

(if you want, you can see discussion in the attached document)

*49. Do you agree in giving this recommendation (eventually in a new version) ?

- Yes
- Yes with suggestions (to be added in next question)
- No

50. What suggestions do you have for improving this recommendation?

*51. Please, rate the importance of this recommendation

- 1 - Very Low
- 2 - Low
- 3 - Medium
- 4 - High
- 5 - Very high

*52. Please, rank the following different versions of this recommendation

	Version 1. We recommend that patients are split into two groups: previously treated and not treated. We recommend not to consider as a previous treatments any approach without proof of efficacy in the literature.
	Version 2. We recommend that patients are split into two groups: previously treated and not treated. We recommend not to consider as a previous treatments any approach without proof of efficacy in the literature according to Cochrane reviews, or level 1 recommendations of current Guidelines (at least one RCT).
	Version 3. We recommend that patients are split into two groups: previously treated and not treated. We recommend not to consider as a previous treatments any approach without proof of efficacy in the literature according to Cochrane reviews, or level 1 recommendations of current Guidelines (at least one RCT). Today, this include bracing but not exercises.

53. Do you suggest any other version / wording for this recommendation ?

Recommendation 11

We recommend to include compliance data, possibly obtained through objective means, and split results according to compliance

(if you want, you can see discussion in the attached document)

*54. Do you agree in giving this recommendation (eventually in a new version) ?

- Yes
- Yes with suggestions (to be added in next question)
- No

55. What suggestions do you have for improving this recommendation?

*56. Please, rate the importance of this recommendation

- 1 - Very Low
- 2 - Low
- 3 - Medium
- 4 - High
- 5 - Very high

*57. Please, rank the following different versions of this recommendation

- Version 1. We recommend to include compliance data, possibly obtained through objective means, and split results according to compliance
- Version 2. We recommend to include compliance data and split results according to these data. Prospective bracing studies must use objective means to monitor compliance.

58. Do you suggest any other version / wording for this recommendation ?

Recommendation 12

In the introduction of a new non-operative treatment for patients during growth, we recommend that the following research steps are followed:

Very short term (only for bracing): Immediate in-brace correction
Short term: 4-6 months of treatment
Medium term: Risser+ 3a staging
End of treatment: At treatment discontinuation
Final results at the end of growth: At least 1 year after treatment discontinuation AND Risser 5 and/or ringapophysis closed
Follow-ups: To be calculated from final results

(if you want, you can see discussion in the attached document)

*59. Do you agree in giving this recommendation (eventually in a new version) ?

- Yes
- Yes with suggestions (to be added in next question)
- No

60. What suggestions do you have for improving this recommendation?

*61. Please, rate the importance of this recommendation

- 1 - Very Low
- 2 - Low
- 3 - Medium
- 4 - High
- 5 - Very high

*62. Please, rank the following different versions of this recommendation

- Version 1. as above
- Version 2. as above but Short term: 12 months of treatment instead of 4-6 months of treatment
- Version 3. as above but abolition of Short term
- Version 4. as above but Risser+ staging 3b (corresponding to actual US Risser 4 and EU Risser 3) instead of Risser+ staging 3a (corresponding to actual US Risser 3 and EU Risser 2)
- Version 5. combination of version 2 (change of Short term) and 4 (change of Risser+)
- Version 6. combination of version 3 (abolition of Short term) and 4 (change of Risser+)

63. Do you suggest any other version / wording for this recommendation ?

Recommendation 13

In the introduction of a non-operative treatment, we recommend that the following level of evidence is followed (and presented in the material and methods section)

Level I: High quality randomized controlled trial (RCT)
Level II Prospective comparative study
Level III: Case control study - Retrospective comparative study
Level IV: Case series

(if you want, you can see discussion in the attached document)

*64. Do you agree in giving this recommendation (eventually in a new version) ?

- Yes
- Yes with suggestions (to be added in next question)
- No

65. What suggestions do you have for improving this recommendation?

*66. Please, rate the importance of this recommendation

- 1 - Very Low
- 2 - Low
- 3 - Medium
- 4 - High
- 5 - Very high

67. Do you suggest any other version / wording for this recommendation ?

Recommendation 14

In the introduction of a new brace, we recommend to focus on the indications proposed by the SRS

(you can find references in the attached document)

(if you want, you can see discussion in the attached document)

*68. Do you agree in giving this recommendation (eventually in a new version) ?

- Yes
- Yes with suggestions (to be added in next question)
- No

69. What suggestions do you have for improving this recommendation?

*70. Please, rate the importance of this recommendation

- 1 - Very Low
- 2 - Low
- 3 - Medium
- 4 - High
- 5 - Very high

*71. Please, rank the following different versions of this recommendation

	Version 1. In the introduction of a new brace, we recommend to focus on the indications proposed by the SRS.
	Version 2. In the introduction of a new brace, we recommend to focus research on the SRS criteria that follows. Inclusion: above 10 years of age, Risser 0-2; curves 25-40° Cobb; no previous treatment; for females: maximum 1 year post-menarche. End of treatment: Risser 3. Outcome: percentage of patients: progressed 6° or more, above 45° at the end of treatment, surgery performed. Follow-up: 2 years.
	Version 3. As version 2, but changing Risser stagings to corresponding Risser+ stagings. In this case it will be added to the recommendation "as modified by this SRS-SOSORT Consensus"
	Version 4. As version 2, but abolishing "females: maximum 1 year post-menarche". In this case it will be added to the recommendation "as modified by this SRS-SOSORT Consensus"
	Version 5. Combination of version 3 and 4

72. Do you suggest any other version / wording for this recommendation ?

Recommendation 15

In presenting results on bracing, we recommend to answer to the questionnaire in Appendix of the SOSORT Guidelines for Management of braced patients to understand how team managed patients

(you can find references in the attached document)

(if you want, you can see discussion in the attached document)

*73. Do you agree in giving this recommendation (eventually in a new version) ?

- Yes
- Yes with suggestions (to be added in next question)
- No

74. What suggestions do you have for improving this recommendation?

*75. Please, rate the importance of this recommendation

- 1 - Very Low
- 2 - Low
- 3 - Medium
- 4 - High
- 5 - Very high

76. Do you suggest any other version / wording for this recommendation ?

Recommendation 16

In presenting results on bracing, we recommend to split results according to the dosage of bracing in terms of impact on patients life, as follows:

Nighttime: Up to 10 hours per day
Home-time: 11-14 hours per day
Half daytime: 15-18 hours per day
Full time: 19-21 hours per day
Total time: 22-24 hours per day

(if you want, you can see discussion in the attached document)

*77. Do you agree in giving this recommendation (eventually in a new version) ?

- Yes
- Yes with suggestions (to be added in next question)
- No

78. What suggestions do you have for improving this recommendation?

*79. Please, rate the importance of this recommendation

- 1 - Very Low
- 2 - Low
- 3 - Medium
- 4 - High
- 5 - Very high

***80. Please, rank the following different versions of this recommendation**

(Comment. A classification of the number of hours of bracing is somehow desirable, but the discussion clearly shows how far we are from any agreement. Studies are very few and sparse. If we will be able to reach at least a low degree of agreement, we will introduce the recommendation otherwise we will avoid it)

	Version 1. Nighttime: Up to 10 hours per day - Home-time: 11-14 hours per day - Half daytime: 15-18 hours per day - Full time: 19-21 hours per day - Total time: 22-24 hours per day
	Version 2. Nighttime: only in bed - Home-time: up to 16 hours per day - Part time: 17-19 hours per day - Full time: 20-22 hours per day - Total time: 23-24 hours per day
	Version 3. Nighttime: only in bed - Home-time: up to 16 hours per day - Part time: 17-20 hours per day - Full time: 20-23 hours per day - Total time: 24 hours per day
	Version 4. Nighttime: only in bed - Home-time: up to 16 hours per day - Part time: 17-20 hours per day - Full time: 20-24 hours per day
	Version 5. Night time: only in bed - Day time not-compliant: 0-6 hours per day +/- night - Partial daytime: 6-10 hours per day +/- night - Full daytime: 11 or more hours per day +/- night - Full time: 20-24 hours per day

81. Do you suggest any other version / wording for this recommendation ?

Recommendation 17

At this stage of research on non-operative approaches during growth other than bracing, we strongly recommend to present radiographic results (mandatory)

(if you want, you can see discussion in the attached document)

*82. Do you agree in giving this recommendation (eventually in a new version) ?

- Yes
- Yes with suggestions (to be added in next question)
- No

83. What suggestions do you have for improving this recommendation?

*84. Please, rate the importance of this recommendation

- 1 - Very Low
- 2 - Low
- 3 - Medium
- 4 - High
- 5 - Very high

*85. Please, rank the following different versions of this recommendation

	Version 1. At this stage of research on non-operative approaches during growth other than bracing, we strongly recommend to present radiographic results (mandatory).
	Version 2. At this stage of research on non-operative approaches during growth other than bracing, we strongly recommend to present radiographic results (mandatory). Results must be presented with a follow-up of adequate length (recommendation 12) and in consistent populations (recommendation 8).

86. Do you suggest any other version / wording for this recommendation ?

Thank you very much !

Thank you for your important help

We wait to see you in Wiesbaden !

87. Do you have any other suggestion ?
