# Supplementary material for: Recommendations for research studies on treatment of idiopathic scoliosis: Consensus 2014 between SOSORT and SRS non–operative management committee
Source: Scoliosis. 2015 Mar 7;10:8. doi: 10.1186/s13013-014-0025-4 (PMC4360938; doi:10.1186/s13013-014-0025-4)
Supplement: Additional file 13: — Questionnaire 4. [file 13013_2014_25_MOESM13_ESM.doc]

# First SRS-SOSORT Consensus (2014)

Title: Recommendations for research studies on non-operative treatment of Idiopathic Scoliosis

## Answers to the question at stage 4

**Rank the following terms according to your preference (to be used in the title and throughout the document). Only the first 5 ranked answers will be retained for further analysis 1. Best 5. Worst From 6 to 12. Eliminated**

| **Rank** | **Proposed definition** | **Average** | **SD** | **Median** | **Preference 1-5** |
| --- | --- | --- | --- | --- | --- |
| **1** | *Bracing and Physiotherapeutic Scoliosis Specific Exercises (PSSE)* | 2.42 | 1.39 | 2 | 121 |
| **2** | *Bracing and Exercises* | 2.62 | 1.35 | 2 | 114 |
| **3** | *Orthopaedic and Rehabilitation Medicine* | 2.75 | 1.27 | 3 | 36 |
| **4** | *Rehabilitation Medicine* | 2.92 | 1.54 | 2 | 14 |
| **5** | *Non­operative* | 2.93 | 1.58 | 3 | 77 |
| **6** | *Conservative* | 3.08 | 1.22 | 3 | 105 |
| **7** | *Non­surgical* | 3.14 | 1.52 | 3 | 66 |
| **8** | *Physical and Rehabilitation Medicine* | 3.21 | 1.28 | 3 | 38 |
| **9** | *Rehabilitation* | 3.23 | 1.52 | 4 | 17 |
| **10** | *Orthopaedic and Rehabilitation* | 3.49 | 1.29 | 4 | 45 |
| **11** | *Functional* | 3.71 | 1.02 | 4 | 63 |
| **12** | *Medical* | 4.33 | 1.13 | 5 | 24 |

The proposal is to make people vote after presenting a table like that in the next page.

Please add to the table your proposed columns and choices to facilitate reaching the best possible decision

I would also suggest to vote each item in this way:

1. Totally acceptable 3 points
2. Acceptable 1 point
3. Usable 0 points
4. Unacceptable -1 point
5. Totally unacceptable -3 points

(+ Advantage; - Limit; ±Intermediate; NA Not applicable)

|  | **Appropriateness** | **Future generalizability** | **Specialty inclusive** | **SRS use** | **SOSORT use** | **Definition by inclusion** | **Tradition** | **Other** |
| --- | --- | --- | --- | --- | --- | --- | --- | --- |
|  | what we do today in this field | it will include future developments | Independency from a specific specialty |  |  | What we do, not what we don’t do |  |  |
| **Bracing and Physiotherapeutic Scoliosis Specific Exercises (PSSE)** | + | - | - | - | + | + | - |  |
| **Bracing and Exercises** | + | - | - | - | + | + | ± |  |
| **Orthopaedic and Rehabilitation Medicine** | + | + | - | ± | + | + | + |  |
| **Rehabilitation Medicine** | ± | ± | - | - | ± | + | - |  |
| **Non­operative** | + | ± | - | + | - | - | - |  |
| **Conservative** | + | ± | + | ± | ± | - | + | Today there is also “conservative surgery” |
| **Non­surgical** | + | ± | - | + | - | - | + |  |
| **Physical and Rehabilitation Medicine** | ± | ± | - | - | - | + | - |  |
| **Rehabilitation** | ± | ± | - | - | - | + | - |  |
| **Orthopaedic and Rehabilitation** | + | ± | - | - | + | + | + |  |
| **Functional** | ± | ± | + | - | - | ± | - | Related to ICF classification Not well known |
| **Medical** | + | + | + | - | - | + | - | The opposite of surgical |

Recommendation 4 (new one)

After the 4th Delphy Survey stage discussion of previous recommendations #5 and #6 (now in this document #7 and #8), it come out the need of two new recommendations to strengthen some of the most underlined concerns of participants. These recommendations will be voted during the Meeting in the same way as for the previous stage 4.

## New version to be voted

We recommend that prognostic factors for consequences of the deformity in adulthood on primary patient-centred outcomes (such as aesthetics, deformity progression, disability, pain and quality of life) are continuously researched and better defined by high quality studies

## Rates to be given

Do you agree with this recommendation ? <> Yes <> Yes with suggestions <> No

Degree of importance: 0 No 1 Low 2 Medium 3 High 4 Maximum

Suggestions: _____________________________________________________________________

________________________________________________________________________________

________________________________________________________________________________

Recommendation 6 (new one)

After the 4th Delphy Survey stage discussion of previous recommendations #5 and #6 (now in this document #7 and #8), it come out the need of two new recommendations to strengthen some of the most underlined concerns of participants. These recommendations will be voted during the Meeting in the same way as for the previous stage 4.

## New version to be voted

We recommend in everyday non-operative clinics to focus not on the secondary predictive outcomes (such as clinical, radiological and topographic data), but on the primary outcomes relevant to the patients (such as aesthetics, disability, pain and quality of life). Clinical, radiological and topographic parameters must be all taken into account for clinical decisions.

## Rates to be given

Do you agree with this recommendation ? <> Yes <> Yes with suggestions <> No

Degree of importance: 0 No 1 Low 2 Medium 3 High 4 Maximum

Suggestions: _____________________________________________________________________

________________________________________________________________________________

________________________________________________________________________________
