# Supplementary material for: Recommendations for research studies on treatment of idiopathic scoliosis: Consensus 2014 between SOSORT and SRS non–operative management committee
Source: Scoliosis. 2015 Mar 7;10:8. doi: 10.1186/s13013-014-0025-4 (PMC4360938; doi:10.1186/s13013-014-0025-4)
Supplement: Additional file 15: — Questionnaire 5. [file 13013_2014_25_MOESM15_ESM.pdf]

First SOSORT SRS Joint Consensus 2014 - final round

Introduction

https://www.surveymonkey.com/s/Z7JG3NJ

ATTENTION

We will vote only during the Meeting

Please, look at your email and connect through wi-fi: you can vote NOW

Agreement and Importance ratings have been given at stages 3 and 4: they are rated according to the following table

Agreement
Answers: Rating
100%: Complete
95-99.9%: High
90-94.9%: Good
80-89.9%: Weak
Below 80%: Absent

Importance
Answers: Rating
4.5-5: Very High
3.5-4.4: High
2.5-3.4: Medium
1.5-2.4: Low
1-1.4: Very Low

Generalities of respondent

*1. What is your first name?

*2. What is your last name?

*3. What is your gender?

- Female
- Male

*4. What is your age?

- 18 to 24
- 25 to 34
- 35 to 44
- 45 to 54
- 55 to 64
- 65 to 74
- 75 or older

*5. Profession

- MD, Orthopedic Surgeon
- MD, Physical and Rehabilitation Medicine
- MD, other
- Physical Therapist
- Orthotist
- PhD
- Other (please specify)

*6. Society of the responder (tick all relevant answers - multiple answers possible)

- SOSORT Member
- SOSORT Executive Committee
- SOSORT Advisory Board
- SRS Non Operative Committee
- SRS Presidential Line

Recommendation 1

High agreement
High importance

7. Which of the following versions do you most prefer for Recommendation 1?

- ○ We recommend ongoing high quality research and development focused on innovative non operative treatments for scoliosis and related spinal deformities
- ○ We recommend that innovative non-operative approaches for all ages and all spinal deformities are continuously researched by high quality studies

Recommendation 2

High agreement
High importance

8. Which of the following versions do you most prefer for Recommendation 2 ?

- ◯ We recommend that indications and contraindications for non-operative approaches are continuously researched by high quality studies
- ◯ We recommend that indications and contraindications for non-operative approaches are regularly updated as new evidence based information is obtained
- ◯ We recommend that standard parameters for non-operative treatment indications and contraindications be continuously developed, maintained and adhered to

Recommendation 3

High agreement
High importance

9. Please, indicate your preferred option to be put in the space in brackets to complete the recommendation:
We recommend that [...] of non-operative treatments be continuously researched by high quality studies

- risks and benefits
- strengths and adverse effects
- strengths and possible adverse effects
- strengths and weaknesses

Recommendation 4 - New

We recommend that prognostic factors for consequences of the deformity in adulthood on primary patient-centred outcomes (such as aesthetics, deformity progression, disability, pain and quality of life) are continuously researched and better defined by high quality studies

*10. Do you agree in giving this recommendation ?

- Yes
- Yes with suggestions (to be added in next question)
- No

*11. Please, rate the importance of this recommendation

- 1 - Very Low
- 2 - Low
- 3 - Medium
- 4 - High
- 5 - Very high

Recommendation 5

High agreement
High importance

*12. Which of the following versions do you most prefer for Recommendation 5 ?

- ○ We recommend to systematically report in clinical studies either the primary patient-centred (such as aesthetics, disability, pain and quality of life), and the secondary predictive (such as clinical, radiological and topographic data) outcomes of non-operative approaches.
- ○ We recommend to systematically report in clinical studies either the primary patient-centred (such a Quality of Life) and the secondary predictive (radiological) outcomes of non-operative approaches.

Recommendation 6 - New

*13. Which of the following versions do you most prefer for Recommendation 6 ?

- We recommend that non-operative clinics should focus primarily on clinical outcomes relevant to patients (such as aesthetics, disability, pain and quality of life), and secondarily on predictive outcomes (such as radiographic and topographic data). Clinical, radiological and topographic parameters must be all taken into account for clinical decisions.
- We recommend that non-operative treatment focus on the primary outcomes relevant to the patient (such as aesthetics, disability, pain, and quality of life) and not on the secondary outcomes such as radiological measurements.

*14. Do you agree in giving this recommendation ?

- Yes
- No

*15. Please, rate the importance of this recommendation

- 1 - Very Low
- 2 - Low
- 3 - Medium
- 4 - High
- 5 - Very high

Recommendation 7

Good agreement
High importance

*16. Which of the following versions do you most prefer for Recommendation 7 ?

- We recommend to report research results in the clinically significant terms of number of patients at start and end of treatment exceeding 10°, 30° and 50° Cobb: epidemiology recognises these as risk thresholds for possible health consequences in adulthood like back pain and curve progression [1-6] [2, 7-9]. In everyday clinics, the importance of these thresholds should be defined case by case in front of single patients according to many parameters other than Cobb degrees.
- We recommend to report research results in the clinically significant terms of number of patients at start and end of treatment exceeding 10°, 30° and 50° Cobb [1-6] [2, 7-9].
- We recommend to report research results in the clinically significant terms of number of patients at start and end of treatment exceeding 10° (scoliosis definition), 30° (increased risk of back pain and progression in adulthood) and 50° Cobb (possible surgical indication) [1-6] [2, 7-9].

Recommendation 8

Good agreement
High importance

*17. Which of the following versions do you most prefer for Recommendation 8 ?

- ○ We recommend giving radiographic research outcomes mainly in clinically significant terms according to Recommendation 7. Radiographic results, if presented, must be given in terms of number of patients improved (6° or more), unchanged (+/-5°) and progressed (6° or more)
- ○ We recommend that radiographic research outcomes are presented in terms of number of patients improved (6° or more), unchanged (+/-5°) and progressed (6° or more)

Recommendation 9

Weak Agreement
High Importance

We recommend the adoption of the SRS-SOSORT “Risser+” staging. This is the result of the confluence between the original US Risser staging, and the so-called European version of Risser staging as modified by Stagnara [10-12]. It has been added also the tryradiate cartilage fusion, that has been shown to be an important and prognostic subdivision of Risser staging 0.

SOSORT-SRS “Risser+” staging 0-: Open Tryradiate cartilage – US and EU RIsser 0
SOSORT-SRS “Risser+” staging 0: Closed Tryradiate cartilage – US and EU RIsser 0
SOSORT-SRS “Risser+” staging 1: 0-25% coverage – US and EU RIsser 1
SOSORT-SRS “Risser+” staging 2: 25-50% coverage – US and EU RIsser 2
SOSORT-SRS “Risser+” staging 3: 50-75% coverage – US Risser 3 –EU RIsser 2
SOSORT-SRS “Risser+” staging 3/4: 75-100% coverage – US Risser 4 –EU RIsser 3
SOSORT-SRS “Risser+” staging 4: start of fusion – US and EU RIsser 4
SOSORT-SRS “Risser+” staging 5: complete fusion – US and EU RIsser 5

*18. Which staging do you prefer ?

- 0-, 0, 1, 2, 3, 3/4,4,5
- 0-, 0+, 1, 2, 3-, 3+,4,5
- 0a, 0b, 1, 2, 3a, 3b, 4, 5
- 0 open, 0 closed, 1, 2, 3, 4, 4+, 5

*19. Do you like maintaining the name Risser or you prefer to avoid it ? Chose the preferred name:

- SOSORT-SRS Risser+ staging
- SOSORT-SRS staging

*20. Would you like to add this sentence to the recommendation “New additional bone maturation parameters coming from the same x-rays should be researched with high quality studies to improve in the future the SOSORT-SRS Risser+ staging” ?

- Yes
- No

Recommendation 10

Weak Agreement
Very High Importance

We recommend that radiographic research outcomes are presented also split in tables according to Cobb degrees at start of treatment (group of 5 degrees Cobb) and bone age (Risser+ staging), like the following one:

Groups: Early Onset (0-5.11 years), Juveniles (6-9.11), Adolescent (10 or more)
Groups in Early Onset must be divided according to age groups:0, 1, 2, 3, 4, 5 years
Groups in Adolescents must be divided according to Risser+ staging
Each group must be divided in sub-groups: Below 10 degrees with a rib hump / lumbar prominence, 11-19 degrees, 20-29 degrees, 30-39 degrees, 40-49 degrees, 50 degrees or more

*21. Which subdivision of the rows do you prefer ?

- Every 10 degrees like it is now
- Every 5 degrees

*22. For the first row “Below 10°”, what do you prefer ?

- "Below 10 degrees with a rib hump / lumbar prominence”, like it is now
- Below 10 degrees

Recommendation 11

High Agreement
High Importance

We recommend that standardised and validated questionnaires are used to report Quality of Life results

No need for voting

Recommendation 12

High Agreement
High Importance

*23. Which of the following versions do you most prefer for Recommendation 12 ?

- ○ We recommend in clinical research to include data on adherence to treatment: statistical analysis should include these data. Prospective bracing studies must use objective means to monitor adherence. Exercises studies must report data on adherence to number and length of assisted sessions, and home-exercise.
- ○ We recommend in clinical research to include data on adherence to treatment, possibly obtained through objective means: statistical analysis should include these data.

Recommendation 13

High Agreement
High Importance

In the introduction of a new non-operative treatment for patients during growth, we recommend that the following research steps are followed:

Type of result: Data analysed

Very short term: In-brace correction
Short term: At least 12 months of treatment
End of bone growth: Risser+ 3/4
End of treatment: At treatment discontinuation
Final results at full bone maturity: Risser 5 and/or ringapophysis closed - Minimum 1 year after end of treatment
Follow-ups: To be calculated from final results

*24. Which Risser 3 should be considered as end of growth ?

- Risser+ 3/4 (corresponding to US Risser 3 and EU Risser 4): 100% coverage
- Risser+ 3 (corresponding to US Risser 3 and EU Risser 2): 75% coverage

Recommendation 14

High Agreement
High Importance

We recommend in research on non-operative treatment this table, from the Oxford Centre for Evidence-Based Medicine 2011 Levels of Evidence (www.cebm.net/index.aspx?o=5653)

No need for voting

Recommendation 15

Weak Agreement
High Importance

*25. Which of the following versions do you most prefer for Recommendation 15 ?

- ○ In the introduction of a new brace, we recommend to focus research on the SRS inclusion criteria [13]: above 10 years of age, Risser 0-2, curves 25-40° Cobb.
- ○ In the introduction of a new brace, we recommend to focus research on the SRS inclusion criteria [13]: above 10 years of age, Risser 0-2, curves 25-40° Cobb, no previous treatment.
- ○ In the introduction of a new brace, we recommend to focus research on the SRS inclusion criteria [13]: above 10 years of age, Risser 0-2, curves 25-40° Cobb, no previous treatment; for females: maximum 1 year post-menarche.

Recommendation 16

High Agreement
Very High Importance

*26. Which of the following versions do you most prefer for Recommendation 16 ?

- We recommend to state in clinical research studies if patients were managed by single professionals or by a team working together. The team work and professional composition should be explained. With this aim, in bracing studies we recommend to answer to the questionnaire in Appendix of the SOSORT Guidelines for Management of braced patients [14] to understand how team managed patients
- In presenting research results on bracing, we recommend to answer to the questionnaire in Appendix of the SOSORT Guidelines for Management of braced patients [14] to understand how team managed patients

27. What suggestions do you have for improving this recommendation?

*28. Please, rate the importance of this recommendation

- 1 - Very Low
- 2 - Low
- 3 - Medium
- 4 - High
- 5 - Very high

*29. Please, rank the following different versions of this recommendation

(Comment. A classification of the number of hours of bracing is somehow desirable, but the discussion clearly shows how far we are from any agreement. Studies are very few and sparse. If we will be able to reach at least a low degree of agreement, we will introduce the recommendation otherwise we will avoid it)

- Version 1. Nighttime: Up to 10 hours per day - Home-time: 11-14 hours per day - Half daytime: 15-18 hours per day - Full time: 19-21 hours per day - Total time: 22-24 hours per day
- Version 2. Nighttime: only in bed - Home-time: up to 16 hours per day - Part time: 17-19 hours per day - Full time: 20-22 hours per day - Total time: 23-24 hours per day
- Version 3. Nighttime: only in bed - Home-time: up to 16 hours per day - Part time: 17-20 hours per day - Full time: 20-23 hours per day - Total time: 24 hours per day
- Version 4. Nighttime: only in bed - Home-time: up to 16 hours per day - Part time: 17-20 hours per day - Full time: 20-24 hours per day
- Version 5. Night time: only in bed - Day time not-compliant: 0-6 hours per day +/- night - Partial daytime: 6-10 hours per day +/- night - Full daytime: 11 or more hours per day +/- night - Full time: 20-24 hours per day

30. Do you suggest any other version / wording for this recommendation ?

Recommendation 17

High Agreement
High Importance

In presenting results on bracing, we recommend to specify results according to the dosage of bracing in terms of impact on patients' social life. Nightime: in bed only. Home-time: at home only (up to 14h). Part-time: at least half a day without the brace (15-18h). Full-time: less than half a day without the brace (19-22h). Total time: almost no pauses (23-24h).

*** 31. Which of the following versions do you most prefer for Recommendation 17 ?**

- ○ Like it is now: Night-time: in bed - Home-time: up to 14h – Part-time: 15-18h – Full-time: 19-22h - Total time: 23-24h
- ○ Night-time: in bed - Home-time: up to 16h – Part-time: 17-20h – Full-time: 21-24h
- ○ Night-time: up to 10h - Part-time 11-16h – Full-time is 17-24h

Recommendation 18

Good Agreement
Very High Importance

***32. Which of the following versions do you most prefer for Recommendation 18 ?**

- ○ At this stage of research on non-operative approaches during growth other than bracing, we strongly recommend to present also radiographic results
- ○ At this stage of research on non-operative approaches during growth other than bracing, we strongly recommend to present radiographic results
- ○ At this stage of research on non-operative approaches during growth other than bracing, we strongly recommend to present radiographic results (mandatory)

Title: Recommendations for research studies on non-operative treatment of I...

According to the answers received, presumably we are not yet mature to define our best choice for a broad definition of what we everyday do. Nevertheless, we think it useful to continue our discussion before ending this Consensus Session

*33. Rank the following terms according to your preference

	Perfect	Acceptable	Indifferent	Not acceptable	Totally not acceptable
Bracing and Exercises	○	○	○	○	○
Bracing and Physiotherapeutic Scoliosis Specific Exercises (PSSE)	○	○	○	○	○
Orthopedic and Rehabilitation Medicine	○	○	○	○	○
Rehabilitation Medicine	○	○	○	○	○
Nonoperative	○	○	○	○	○
Conservative	○	○	○	○	○
Nonsurgical	○	○	○	○	○
Physical and Rehabilitation Medicine	○	○	○	○	○
Rehabilitation	○	○	○	○	○
Orthopedic and Rehabilitation	○	○	○	○	○
Functional	○	○	○	○	○
Medical	○	○	○	○	○

*34. Which of the following versions do you most prefer for the title ?

○ Recommendations for research studies on Treatment of Idiopathic Scoliosis

○ Recommendations for research studies on Idiopathic Scoliosis: Bracing, Specific Physiotherapeutic Scoliosis Exercises, or other Functional fusion-less treatments.

○ Recommendations for research studies on Idiopathic Scoliosis: Bracing, Specific Physiotherapeutic Scoliosis Exercises, or other fusion-less treatments.
