# Supplementary material for: Recommendations for research studies on treatment of idiopathic scoliosis: Consensus 2014 between SOSORT and SRS non–operative management committee
Source: Scoliosis. 2015 Mar 7;10:8. doi: 10.1186/s13013-014-0025-4 (PMC4360938; doi:10.1186/s13013-014-0025-4)
Supplement: Additional file 16: — Final results. [file 13013_2014_25_MOESM16_ESM.doc]

# Recommendations for Research Studies on Treatment of Idiopathic Scoliosis

1. We recommend ongoing high quality research and development focused on innovative non operative treatments for scoliosis and related spinal deformities (B2)
2. We recommend that indications and contraindications for non-operative approaches are continuously researched by high quality studies (B2)
3. We recommend that risks and benefits of non-operative treatments be continuously researched by high quality studies (B2)
4. We recommend that prognostic factors for consequences of the deformity in adulthood on primary patient-centred outcomes (such as aesthetics, deformity progression, disability, pain and quality of life) are continuously researched and better defined by high quality studies (A2)
5. We recommend to systematically report in clinical studies the primary patient-centred (such as aesthetics, disability, pain and quality of life), and the secondary predictive (such as clinical, radiological and topographic data) outcomes of non-operative approaches (B2)
6. We recommend that non-operative clinics should focus primarily on clinical outcomes relevant to patients (such as aesthetics, disability, pain and quality of life), and secondarily on predictive outcomes (such as radiographic and topographic data). Clinical, radiological and topographic parameters must be all taken into account for clinical decisions (D2)
7. We recommend to report research results in the clinically significant terms of number of patients at start and end of treatment exceeding 10°, 30° and 50° Cobb: epidemiology recognises these as risk thresholds for possible health consequences in adulthood like back pain and curve progression [1-6] [2, 7-9]. In everyday clinics, the importance of these thresholds should be defined case by case in front of single patients according to many parameters other than Cobb degrees (C2)
8. We recommend that radiographic research outcomes are presented in terms of number of patients improved (6° or more), unchanged (+/-5°) and progressed (6° or more) (C2)
9. We recommend the adoption of the “risser+” staging. This is the result of the confluence between the original us risser staging, and the so-called european version of risser staging as modified by stagnara [10-12]. It has been added also the tryradiate cartilage fusion, that has been shown to be an important and prognostic subdivision of risser staging 0. (D2)

| **“Risser+” staging** | **Tryradiate cartilage ossification** | **US Risser staging** | **European Risser staging** |
| --- | --- | --- | --- |
| **0-** | No | 0 | 0 |
| **0** | Yes | 0 | 0 |
| **1**  **0-25% coverage** |  | 1  0-25% coverage | 1  initial ossification |
| **2**  **25-50% coverage** |  | 2  25-50% coverage | 2  partial coverage |
| **3**  **50-75% coverage** |  | 3  50-75% coverage |  |
| **3/4**  **75-100% coverage** |  | 4  75-100% coverage | 3  complete coverage |
| **4**  **start of fusion** |  |  | 4  start of fusion |
| **5**  **complete fusion** |  | 5  complete fusion | 5  complete fusion |

1. We recommend that radiographic research outcomes are presented also split in tables according to Cobb degrees at start of treatment (group of 5° Cobb) and bone age (Risser+ staging), like the following one (D2):

|  | **Early Onset** | | | | | **Juvenile** | **Adolescent** | | | | | | |
| --- | --- | --- | --- | --- | --- | --- | --- | --- | --- | --- | --- | --- | --- |
| *Age at start of treatment* | *0* | *1* | *2* | *3* | *4-5* | *6-9* | *10 or more* | | | | | | |
| *Risser+ staging* |  |  |  |  |  |  | *0-* | *0* | *1* | *2* | *3* | *3/4* | *4* |
| **Below 10°** (**with a rib hump / lumbar prominence)** |  |  |  |  |  |  |  |  |  |  |  |  |  |
| **11-19°** |  |  |  |  |  |  |  |  |  |  |  |  |  |
| **20-29°** |  |  |  |  |  |  |  |  |  |  |  |  |  |
| **30-39°** |  |  |  |  |  |  |  |  |  |  |  |  |  |
| **40-49°** |  |  |  |  |  |  |  |  |  |  |  |  |  |
| **50° or more** |  |  |  |  |  |  |  |  |  |  |  |  |  |

1. We recommend that standardised and validated questionnaires are used to report Quality of Life results (B2)
2. We recommend in clinical research to include data on adherence to treatment: statistical analysis should include these data. Prospective bracing studies must use objective means to monitor adherence. Exercises studies must report data on adherence to number and length of assisted sessions, and home-exercise (B2)
3. In the introduction of a new non-operative treatment for patients during growth, for the radiographic outcome we recommend that the following research steps are followed: (B2)

| **Type of result** | **Data analysed** |
| --- | --- |
| **Very short term** | In-brace correction |
| **Short term** | At least 12 months of treatment |
| **End of bone growth** | Risser+ 3/4 |
| **End of treatment** | At treatment discontinuation |
| **Final results at full bone maturity** | Risser 5 and/or ringapophysis closed  Minimum 1 year after end of treatment |
| **Follow-ups** | To be calculated from final results |

1. We recommend in research on non-operative treatment this table, from the Oxford Centre for Evidence-Based Medicine 2011 Levels of Evidence ([www.cebm.net/index.aspx?o=5653](http://www.cebm.net/index.aspx?o=5653)) (B2)

| **Type of research** | **Treatment**  **benefits / harms** | **Diagnosis** | **Prognosis** | **Screening** |
| --- | --- | --- | --- | --- |
| ***The question*** | *Does this intervention*  *help / harm?* | *Is this diagnostic-monitoring test accurate?* | *What will happen if we do not add a therapy?* | *Is this early detection test worthwhile?* |
| **Level I** | Systematic review of RCTs | Systematic review of cross-sectional studies with consistently applied gold standard and blinding | Systematic review of inception cohort studies | Systematic review of RCTs |
| **Level II** | RCT  Prospective controlled cohort study | Cross-sectional study with consistently applied gold standard and blinding | Inception cohort study (patients enrolled at same stage of their disease) | RCT |
| **Level III** | Retrospective controlled cohort study  Follow-up study | Study of non-consecutive patients  Study without consistently applied gold standard | Cohort study  Control arm of RCT | Controlled cohort study  Follow-up study |
| **Level IV** | Case-series  Case-control study  Historically controlled study | Case-control studies  Poor or non-independent gold standard | Case-series  Case-control study  Poor quality prognostic cohort study | Case-series  Case-control study  Historically controlled study |
| **Level V** | Mechanism-based reasoning | Mechanism-based reasoning | --- | Mechanism-based reasoning |

1. In the introduction of a new brace, we recommend to focus research on the following SRS inclusion criteria [13]: above 10 years of age, Risser 0-2, curves 25-40° Cobb. (D2)
2. In presenting research results on bracing, we recommend to answer to the questionnaire in Appendix of the SOSORT Guidelines for Management of braced patients [14] to understand how team managed patients. (B3)
3. In presenting results on bracing, we recommend to specify results according to the dosage of bracing in terms of impact on patients’ social life. (B2)

| **Nightime** | In bed only |
| --- | --- |
| **Home-time** | At home only (up to 14h) |
| **Part-time** | At least half a day without the brace (15-18h) |
| **Full-time** | Less than half a day without the brace (19-22h) |
| **Total time** | Almost no pauses (23-24h). (B2) |

1. At this stage of research on non-operative approaches during growth other than bracing, we strongly recommend to present radiographic results (C2)

# First SRS-SOSORT Consensus (2014)

- Results relate to the recommendation listed, in the version proposed at each stage (see specific questionnaire)
- Discussion 1-2 refers to the first two stages, when this format of recommendations was not adopted, but the topics were already under discussion.
- Discussion 3 and 4 relate to the various stages of development of the recommendations
- Discussions 3 and 4 imply by respondents reading previous stages of discussion and votes.
- Agreement and Importance are rated according to the following table

| **Classifications** | | | |
| --- | --- | --- | --- |
| **Agreement** | | **Importance** | |
| **Yes-No** | | **1-Very Low ; 2-Low ; 3-Medium ; 4-High ; 5-Very High** | |
| *Answers* | *Rating* | *Answers* | *Rating* |
| 100% | A - Complete | 4.5-5 | 1- Very High |
| 95-99.9% | B - High | 3.5-4.4 | 2- High |
| 90-94.9% | C - Good | 2.5-3.4 | 3- Medium |
| 80-89.9% | D - Weak | 1.5-2.4 | 4- Low |
| Below 80% | Absent | 1-1.4 | 5- Very Low |

Recommendation 1. We recommend ongoing high quality research and development focused on innovative non operative treatments for scoliosis and related spinal deformities (B2)

## Results

| **Stage of Delphi Survey** | **Agreement** | | **Importance** | | |
| --- | --- | --- | --- | --- | --- |
|  | *Rating* | *%* | *Rating* | *Average* | *Median* |
| **3** | Complete | 100% | High | 3.93 | 4 |
| **4** | High | 98.56% | High | 4.07 | 4 |

## Results of the Consensus

| **Versions voted** | **Percentage of preferences** |
| --- | --- |
| We recommend ongoing high quality research and development focused on innovative non operative treatments for scoliosis and related spinal deformities | 67.4% |
| We recommend that innovative non-operative approaches for all ages and all spinal deformities are continuously researched by high quality studies | 32.6% |

## Discussion 4

Focused on the different therapies, age groups and strength of evidence according to sensibilities of the respondents. An interesting comment that strengthen the importance of this recommendation is: “We recently had a prominent orthopaedic journal reject a very well done randomized clinical trial because ‘insurance companies will not likely cover the treatment’. This stifles development of new innovative methods to improve deformities of the spine. I believe that education of journal reviewers is very important. Perhaps publishing position statements from consortium groups can help this particularly if editors promote these consensus opinions to their reviewers.” (Joseph Betz).

Bernard Jean-Claude speak with a 3D evaluation

Bettany-Saltikov Josette are continuously researched and evidenced based results produced and published

Betz Joseph We recently had a prominent orthopaedic journal reject a very well done randomized clinical trial because “insurance companies will not likely cover the treatment. This stifles development of new innovative methods to improve deformities of the spine. I believe that education of journal reviewers is very important. Perhaps publishing position statements from consortium groups can help this particularly if editors promote these consensus opinions to their reviewers.

Białek Marianna start treatment as soon as possible with the least angle of curvature

Brox Jens Ivar RCT

Chou Chungwai SOSORT&SRS need to be more open-minded to any and all new approaches from all over the world.

Dairiany Tetty Murniaty More research for non operative approach such as soft bracing

de Ru Esther And integrated into treatment list of options

Drake Shawn More emphasis on evidence based practice related to PSE.

Espinoza Pamela Many types of non operative approaches for treating scoliosis are mentioned on literature. Almost of them have a lack of strong evidence that supports the therapy. I suggest that is necessary define what types of exercises are the best for treating scoliosis or unify the scoliosis schools for create one school that has the best of all of them. It’s my opinion.

Hennes Axel should specified for all types of scoliosis

Henning Susan New approaches should recognize tri-planar asymmetry as a fundamental concept & new techniques should incorporate this understanding.

Kerstholt Janine early start with PSSE and suggestions for specific braces for specific age/spinal deformity

Laura Djuriantina More study for non operative approaches

Lebel Andrea I would differentiate the urgency on JIS, AIS and other, according to prevalence, and the speed of progression.

Marcotte Louise Include specific spinal manipulation in the new version of the recommendation

Marti Cindy I recommend resources be prioritized first at studying with high-level control groups, existing SOSORT methods prior to methods that are unknown or completely unpublished. Must be SCOLIOSIS SPECIFIC ex methods, not general

Matthews Martin Acceptance of new orthotic developments using audit and retrospective studies to offer new opportunities for further developmental research.

Monroe Marcia Specific physical therapy, and exercises as well as Iyengar Yoga therapy(it is not a traditional yoga regimen but specified and based on bio mechanics)

Muccio Marissa clear logical underlying theory of mechanism of action

O’Brien Joseph Need Innovations, whether improvements of existing treatment, or new methods and/or devices.

Parent Eric I am not sure they have to be NEW. As long as there evidence based is insufficient. Should we limit this consensus to AIS. Chances are many of the recommendations to follow are specific to scoliosis.

Pizzetti Paolo create national and international study groups

Rivett LouAnn More studies need to be done, improve quality of research.

Simony Ane better guidelines and research in the field of different braces, and effect of exercises

Speers David Need to continuously explore since more research is being done and more methods are being used for non operative approaches to spinal deformities

Tassone Channing specific phrases ‘all’ ages and ‘all’ spinal deformities are too broad

Tomasz Karski C a u s a l prophylaxis in context of “biomechanical aetiology of the so called idiopathic scoliosis” (T. Karski 1995 – 2007)

Torres Beatriz Education to parents and general community for earlier observations

Van Loon Piet To think asymmetrical in scoliosis, but act symmetrical in lifestyle advise, exercise and bracing. , as in all kyphotic spines.

Wong M. S. Enhancement of brace fitting and long-term monitoring

## Suggestions for new versions

Bernard Jean-Claude with a compete approach: aesthetic, functional, mobility... 3D and in the future without rx frontal and sagittal alone

Bettany-Saltikov Josette as written above but with the words above added at the end of the sentence.

Brox Jens Ivar are continuously explored by high quality studies

Dairiany Tetty Murniaty Funtional improvement

Laura Djuriantina Soft brace

Lebel Andrea We recommend that non-operative New ( evidence based and research based) approaches For all ages and all spinal deformities are continuously explored

Marcotte Louise Chiropractic spinal adjusting and osteopathic manipulation

Marti Cindy Explore new but study methods that have been already published with low-level of evidence. Focus on CONTROLLED studies as this is the criticism we usually get about lack of research for exercise. Be sure again it is SCOLIOSIS SPECIFIC ex, not general PT

Matthews Martin We recommend that new and innovative non- operative approaches for all ages and all sources of spinal deformities are continuously explored.

Monroe Marcia Manual manipulation and alternative methods such as myofascia therapy, acupuncture.

Muccio Marissa Question the use of “new”, is that chronological or new to existing definitions

O’Brien Joseph We recommend ongoing research and development focused on innovative non operative treatments for scoliosis and related spinal deformities.

Parent Eric We recommend that non-operative approaches that are novel or with 5ecause5usly supporting evidence be continuously explored for all ages and all spinal deformities.

Pizzetti Paolo cohoperate

Van Loon Piet Restore optimal extension and lordosis at the Thoracolumbar joint. It houses the counus medullaris.

Wong M. S. Validation of dosage-response treatment

Recommendation 2. We recommend that indications and contraindications for non-operative approaches are continuously researched by high quality studies (B2)

## Results

| **Stage of Delphi Survey** | **Agreement** | | **Importance** | | |
| --- | --- | --- | --- | --- | --- |
|  | *Rating* | *%* | *Rating* | *Average* | *Median* |
| **3** | Complete | 100% | High | 3.87 | 4 |
| **4** | High | 97.06% | High | 4.02 | 4 |

## Results of the Consensus

| **Versions voted** | **Percentage of preferences** |
| --- | --- |
| We recommend that indications and contraindications for non-operative approaches are continuously researched by high quality studies | 55.3% |
| We recommend that indications and contraindications for non-operative approaches are regularly updated as new evidence based information is obtained | 38.3% |
| We recommend that standard parameters for non-operative treatment indications and contraindications be continuously developed, maintained and adhered to | 6.4% |

## Discussion 3

Price. Consider ground rules for inclusion into this category ie certain threshold level of evidence

## Discussion 4

Discussion focuses on the different therapies but also on the importance of evidence according to sensibilities of the respondents.

Bernard Jean-Claude study the quality of life patients with scoliosis

Bettany-Saltikov Josette and researched as well as evidence based results published.

Bissolotti Luciano Define in a better way the criteria of cut off levels to establish a specific non operative interventions: grade of deformity, functional impairment, psychological impairment, functional limitations in daily life activities (even for adult deformities and even more fore neurogenic deformities of adult age)

Dairiany Tetty Murniaty Exercise and posture correction

Dr Fodor Janosné Not priority,we already know a lot

Durmala Jacek with aethiology analysis and different recommendations for different aims (goals)

Fabris Monterumici Daniele evidence based studies

Henning Susan I think it is better to err on the side of trial of non-operative treatment unless there are clear dangers in pursuing conservative treatment. Even if surgery is required at a later time, the person will have benefitted from a better understanding of their alignment and heightened self awareness, as well as techniques to assist self re- positioning.

Laura Djuriantina Some exercise

Lebel Andrea We do have this knowledge already important to reach an agreement with SRS members re: indications

Monroe Marcia Assessing the outcome based on patient feedback.

O’Brien Joseph Standards be developed, maintained and adhered to.

Rivett LouAnn More research

Sieteski Wojciech focus on people with obesity

Stoliński Łukasz There is a lot of conservative methods on the worlds science now, and we should look for all this ways in treatment, for INDICATIONS AND CONTRAINDICATIONS with zooming on their own experience

Tomasz Karski Check / confirm the knowledge presented 19 years [www.ortopedia.karski.lublin.pl](http://www.ortopedia.karski.lublin.pl/)

Torres Beatriz Improving technology

Wong M. S. Going for evidence-based recommendations

## Suggestions for new versions

Bettany-Saltikov Josette as above with the addition on the phrase above

Bissolotti Luciano INDICATIONS AND CONTRAINDICATIONS for non-operative approaches FOR ORTHOPEDIC AND NEUROGENIC DEFORMITIES

Dairiany Tetty Murniaty Corrective movement

Laura Djuriantina Corectiive posture

Lebel Andrea We recommend that INDICATIONS AND CONTRAINDICATIONS for non-operative approaches are regularly updated by the non-surgical service providers and surgeons and researchers as new evidence based information is obtained

O’Brien Joseph We recommend that standard parameters for non operative treatment indications and contraindications be continuously developed , maintained and adhered to.

Van Loon Piet Reset the complete system to the wanted and optinal function an morphology by extension of the entire spine. Avoid flexion.

Recommendation 3. We recommend that risks and benefits of non-operative treatments be continuously researched by high quality studies (B2)

## Results

| **Stage of Delphi Survey** | **Agreement** | | **Importance** | | |
| --- | --- | --- | --- | --- | --- |
|  | *Rating* | *%* | *Rating* | *Average* | *Median* |
| **3** | Complete | 100% | High | 3.80 | 4 |
| **4** | High | 97.79% | High | 3.96 | 4 |

## Results of the Consensus

We recommend that … of non-operative treatments be continuously researched by high quality studies

| **Versions voted** | **Percentage of preferences** |
| --- | --- |
| risks and benefits | 44.4% |
| strengths and weaknesses | 26.7% |
| strengths and adverse effects | 20.0% |
| strengths and possible adverse effects | 8.9% |

## Discussion 4

Discussion focuses on adverse effects, with respondents who prefer to avoid or reduce the importance of this term, or think that they do not exist. Evidence importance continuously stressed

Bettany-Saltikov Josette are 7ecause7usly explored and researched and evidence based data produced and published.

Betz Joseph Caution should be a top priority when making such positions. There is considerable bias from “developers”

Dairiany Tetty Murniaty Core exercise

de Ru Esther and possible adverse effects

Henning Susan There has been some exploration of the negative psychological effects of forms of conservative treatment. Better understanding of age appropriate, conservative interventions could be helpful in designing rehabilitation programs. Also, identifying positive , empowering aspects of conservative treatment could also help guide rehab.

Kerstholt Janine good education programs for all therapists ((para)medical and bracist)

Laura Djuriantina Hydrotherapy

Lebel Andrea Documentation of objective measurements could be obtained from treating practitioners and that can inform us about the results of STRENGTHS and ADVERSE EFFECTS for non-operative approaches.

Marti Cindy Include objective measures and subjective measures; evaluate BEYOND THE COBB angle radiologically (rotation, coronal imbalance and sagittal); include RESPIRATORY measures in and out of brace; include QOL measures besides SR22 because it is often not really reflective of QOL for teens who 7ecau have pain. Create a balanced evaluation of (+) and (-) effects and find a way to then reflect an overall “outcome” that accounts for both, for instance, not just saying “this was bad” with out “this was good”

O’Brien Joseph Sort of a repeat of previous question about ongoing research and development. If used, then Define strengths.......Positive outcomes and adverse affects? Also is explore enough?

Parent Eric I believe both of these should split into different recommendations. Strengths should be defined?

Rivett LouAnn research papers

Stoliński Łukasz For good effects of our exercises we should to know both STRENGTHS and ADVERSE EFFECTS based on EBM and EBP

Tomasz Karski check / control all what is printed (1995 – 2014)according the “biomechanical aetiology of the so called idiopathic scoliosis’

Van Loon Piet get an uniform set of non-operative approaches, that can be understood by patients and all involved

Wajchenberg Marcelo I prefer the following phrase: We recommend that STRENGTHS and EFFECTS for non-operative approaches are continuously explored. (without adverse)

Wynne James Provided we have clear definitions of Strengths and Adverse effects – and a ranking system of each

## Suggestions for new versions

Lebel Andrea We recommend that STRENGTHS and ADVERSE EFFECTS for non-operative approaches are explored periodically from objective data collected from treating practitioners

Marti Cindy I alos recommend evaluating short term and long term effects of non-operative approaches. Please evaluate beyond “preventing surgery” .If Cobb is to be measured then factor Cobb outcome affect related to initial risk of progression (Lonstein Carlson). Reducing curve is not the only sign of positive outcome.

Monroe Marcia The suggestions are to be adapted according to the age group and life style.

O’Brien Joseph We recommend that the risks and benefits of non operative treatments for scoliosis be continuously studied and published.

Rivett LouAnn Instead of ‘adverse effects’ use the word weaknesses

Recommendation 4. We recommend that prognostic factors for consequences of the deformity in adulthood on primary patient-centred outcomes (such as aesthetics, deformity progression, disability, pain and quality of life) are continuously researched and better defined by high quality studies (A2)

## Results

After the 4^th^ Delphy Survey stage discussion of previous recommendations #5 and #6 (now in this document #7 and #8), it come out the need of two new recommendations to strengthen some of the most underlined concerns of participants.

| **Stage of Delphi Survey** | **Agreement** | | **Importance** | | |
| --- | --- | --- | --- | --- | --- |
|  | *Rating* | *%* | *Rating* | *Average* | *Median* |
| **5** | Complete | 100 | High | 4.4 | 4 |
| **6 (Consensus)** | Complete | 100 | High | 4 | 3.79 |

Recommendation 5. We recommend to systematically report in clinical studies the primary patient-centred (such as aesthetics, disability, pain and quality of life), and the secondary predictive (such as clinical, radiological and topographic data) outcomes of non-operative approaches (B2)

## Results

| **Stage of Delphi Survey** | **Agreement** | | **Importance** | | |
| --- | --- | --- | --- | --- | --- |
|  | *Rating* | *%* | *Rating* | *Average* | *Median* |
| **3** | Complete | 100% | High | 4.00 | 4 |
| **4** | High | 98.47% | High | 4.11 | 4 |

## Results of the Consensus

| **Versions voted** | **Percentage of preferences** |
| --- | --- |
| We recommend to systematically report in clinical studies the primary patient-centred (such as aesthetics, disability, pain and quality of life), and the secondary predictive (such as clinical, radiological and topographic data) outcomes of non-operative approaches | 66.7% |
| We recommend to systematically report in clinical studies the primary patient-centred (such a Quality of Life) and the secondary predictive (radiological) outcomes of non-operative approaches | 33.3% |

## Discussion 3

According to the Cochrane Institute primary outcomes are those focused on the patient, while secondary outcomes are those focused on biological and instrumental data that predict possible results on the patient but are not immediately a symptom.

Bettany-Saltikov. For many physical therapists working in private practice as well as in public hospitals it is not possible to obtain x-rays- besides I thought one of our aims was to try and decrease the amount of radiation we are giving to patients. I WOULD really like to see Surface topography as an outcome that is systematically reported as well as other patient oriented outcomes as many PTs have not got access to x-rays. The Cochrane back group do not consider x-rays to be primary patient oriented outcomes. We should also be looking at including some of the following below

Zaina. I would add the ATR measure

Knott. Radiographic reports as indicated, without exposing subjects to excess radiation.

De Mauroy. Regarding radiographic evaluation, must be discussed the surface topography, low dose radiology and 3D EOS and high dose radiology (30x90 and digital segmental). Questionnaires are increasingly criticized in particular by Jean Dubousset in France and their validity discussed. What is the difference between 4 & 9?

Grivas. We have for the operative treatment the SRS 22 questionnaire and for the brace treatment the BrQ to use

## Discussion 4

Discussion focuses on other type of reports including imaging and clinical evaluation. Moreover it is suggested to move Quality of Life in the first place. The importance of defining uniform reports is stressed.

Bettany-Saltikov Josette Could we add topographical data too as many PT may not be able to produce x-ray data?

Bissolotti Luciano Next level of discussion should be focused in the definition of a common minimal dataset of measures on frontal, sagittal and coronal plane.

Brox Jens Ivar Quality of life first

Dairiany Tetty Murniaty Uniformed report

Diers Helmut Radiographs and /or other equal measurement modalities

Drake Shawn The consistent use of QOL outcomes is needed between clinicians.

Espinoza Pamela I agree with Bettany-Saltikov. Why to use X-Rays if there are new technologies as Surface Topography that are less harmful to the patient. I have a patient who had been exposed at 45 X-RAYS in his entire life in a period of nine years. It’s too much.

Fabris Monterumici Daniele clinical evaluation of rib hump

Glinkowski Wojciech Patient Reported Outcomes should be unified. Radiographic and non radiographic methods should rather be employed.

Grivas Theodoros Use of The BrQ for the brace treatment

Hennes Axel radiographic and other methods for scoliosis measurement and documentation

Henning Susan It would be beneficial to minimize x-ray exposure of adolescents, or any patient. Development of other forms of visualizing spinal & rib cage configuration would be a worthwhile goal.

Kerstholt Janine better and uniform questions in the SRS

Kluszczyński Marc Yes, but the assessment by questionnaire Quality of Life should be done only twice e.g. during the first clinical examination and after one year, similarly x-ray examination.

Laura Djuriantina Uniformed report

Luchsinger-Lang Cornelia Quality of Life seems for me more important

Marti Cindy Include 3 D radiograph measures, look for an alternative QOL measures, not just SR22 as i don’t find it to be very reflective when a pt has no pain or is just starting PT

Maude Erika Having a set measure for Quality of Life, so that it is easier to compare methods.

Parent Eric I agree with Josette that requiring radiographs may not be appropriate in some reports. Objective postural measurements may be relevant as well as mechanistic measurements (EG exs targeting endurance of muscle could report changes in this variable.)

Rivett LouAnn Use of questionnaires for all patients

Rosellini Guerrino no exposition to excess rx

Sanchez Judith I,m agree with Dr. Grivas. We use TAPS too, before and after treatment.

Satyawati Rwahita I prefer surface topography rather than x-ray

Simony Ane both in a short time and after 5-10-20 years, so we can continue to guide the patients and their parents about their childrens future.

Stoliński Łukasz We should to look more for contact of patients and their parents, 10ecause in some age, especially in earlier years of life, we must to listen more parents then looking for Quality of life I think

Tomasz Karski radiographic examination every 6 month or every 1 year

Torres Beatriz In many under develop countries, in small towns Radiology services are not available

Van Loon Piet clinical assessment and simple questions will do

Wong M. S. Trying to use other assessment method to reduce/eliminate the radiation exposure such as US.

## Suggestions for new versions

Czaprowski Dariusz I suggest to include in “Other possible evaluations: physical capacity and functionality of the musculoskeletal system”

Dolan Lori We recommend systematic reporting of the outcomes of non-operative approaches, including quality of life and physical outcomes.

Dr Fodor Janosné Photo or video

Glinkowski Wojciech Patient Reported Outcomes may replace Quality of Life

Kluszczyński Marc I think , on the basis of twenty years experience in treatment of children and adolescents with IS, I suggest the following version: Version (X): We recommend to assess systematically Angle of Trunk Rotation( ATR), report systematically radiographic outcomes of non-operative approaches. Other possible evaluations e.g. Quality of Life outcomes and surface topography should be done during a very long period of treatment.

Marti Cindy Specify radiographic data to be 3D. Include aesthetic parameters in secondary outcome and/or patient centred outcomes...because i believe pts care about their aesthetics, so it could be a patient centred outcome, and as a PT working with biomechanics and function, torso symmetry could be relevant to the predictive consequence to the patient

Parent Eric I would want us to recommend mechanistic measurements be taken . for example we could track is muscle endurance exercises indeed lead to change in the targeted impairment. I have issues with the existing QOL tools. I strongly favour postural measurements and does not understand the reluctance to consider these as primary?

Tomasz Karski in discussion

Van Loon Piet report systematically the functional assessment, other data than only x-rays (like formometric scans) and add items in questionnaires on understanding of diagnosis, understanding of the suggested treatment, satisfaction with doctor, with brace, with team approach etc.

Wong M. S. Trying to use other assessment method to reduce/eliminate the radiation exposure such as US.

Wynne James Other possible evaluations may include but not limited to: .....

Recommendation 6. We recommend that non-operative clinics should focus primarily on clinical outcomes relevant to patients (such as aesthetics, disability, pain and quality of life), and secondarily on predictive outcomes (such as radiographic and topographic data). Clinical, radiological and topographic parameters must be all taken into account for clinical decisions (D2)

## Results

After the 4^th^ Delphy Survey stage discussion of previous recommendations #5 and #6 (now in this document #7 and #8), it come out the need of two new recommendations to strengthen some of the most underlined concerns of participants.

| **Stage of Delphi Survey** | **Agreement** | | **Importance** | | |
| --- | --- | --- | --- | --- | --- |
|  | *Rating* | *%* | *Rating* | *Average* | *Median* |
| **5** | Complete | 100% | High | 4.4 | 4 |
| **6 (Consensus)** | Weak | 84.6% | High | 3.61 | 4 |

## Results of the Consensus

| **Versions voted** | **Percentage of preferences** |
| --- | --- |
| We recommend that non-operative clinics should focus primarily on clinical outcomes relevant to patients (such as aesthetics, disability, pain and quality of life), and secondarily on predictive outcomes (such as radiographic and topographic data). Clinical, radiological and topographic parameters must be all taken into account for clinical decisions | 87.2% |
| We recommend that non-operative treatment focus on the primary outcomes relevant to the patient (such as aesthetics, disability, pain, and quality of life) and not on the secondary outcomes such as radiological measurements | 12.8% |

## Discussion 5

**Fabio Zaina.** The point is relevant, but we must balance very well this proposal, otherwise we run the risk that relevant parameters for prognosis wouldn’t be used anymore by people not working properly on the conservative treatment.

**Patrick Knott.** We recommend that non-operative treatment focus on the primary outcomes relevant to the patient (such as aesthetics, disability, pain, and quality of life) and not on the secondary outcomes such as radiological measurements.

Recommendation 7. We recommend to report research results in the clinically significant terms of number of patients at start and end of treatment exceeding 10°, 30° and 50° Cobb: epidemiology recognises these as risk thresholds for possible health consequences in adulthood like back pain and curve progression [1-6] [2, 7-9]. In everyday clinics, the importance of these thresholds should be defined case by case in front of single patients according to many parameters other than Cobb degrees (C2)

## Results

| **Stage of Delphi Survey** | **Agreement** | | **Importance** | | |
| --- | --- | --- | --- | --- | --- |
|  | *Rating* | *%* | *Rating* | *Average* | *Median* |
| **3** | Complete | 100% | High | 4.00 | 4 |
| **4** | Good | 94.47% | High | 3.87 | 4 |

## Results of the Consensus

| **Versions voted** | **Percentage of preferences** |
| --- | --- |
| We recommend to report research results in the clinically significant terms of number of patients at start and end of treatment exceeding 10°, 30° and 50° Cobb: epidemiology recognises these as risk thresholds for possible health consequences in adulthood like back pain and curve progression [1-6] [2, 7-9]. In everyday clinics, the importance of these thresholds should be defined case by case in front of single patients according to many parameters other than Cobb degrees | 56.4% |
| We recommend to report research results in the clinically significant terms of number of patients at start and end of treatment exceeding 10° (scoliosis definition), 30° (increased risk of back pain and progression in adulthood) and 50° Cobb (possible surgical indication) [1-6] [2, 7-9] | 30.8% |
| We recommend to report research results in the clinically significant terms of number of patients at start and end of treatment exceeding 10°, 30° and 50° Cobb [1-6] [2, 7-9] | 12.8% |

## Discussion 1-2

**Price**. I think the 30° threshold is quite arbitrary.

**Negrini**. In reality in the literature the 30° threshold has been reported as important for low back pain

**Channing Tassone**. Agree with concern of 30 degree classification. Want to avoid message that the 30 – 50 degree curves will have issues as adults

**Maruyama**. 10 degrees instead of 11 degrees

**O'Brien**. "J Bone Joint Surg Am. 1983 Apr;65(4):447-55. Curve progression in idiopathic scoliosis. Weinstein SL, Ponseti IV. “In general, curves that were less than 30 degrees at skeletal maturity tended not to progress regardless of curve pattern.”

**Stikeleather**. Are you suggesting identifying these 3 thresholds as significant to treatment decision making or prognosis or????

## Discussion 3

The problem of giving these thresholds as reference terms and not as fixed points, so to be risk factors of possible (but obviously not guaranteed) future consequences is relevant for the patients as well as for the practitioners

**Bettany-Saltikov**. Remove “increased possibility of back pain and progression in adulthood”. Also to add a number of other outcomes besides x-rays measurements. I agree with De Mauroy here

**De Mauroy**. Remove “increased possibility of back pain and progression in adulthood”. Threshold 1: 10° definition of scoliosis – OK. Threshold 2: 30° increased possibility of back pain and progression in adulthood - This is probably true for thoracic curves, but not for lumbar curves. Back pain concerns more lumbar scoliosis. Furthermore, Scoliosis concerns 2% of teenagers, but 10% of the population at 65 years. This means that 8 out of 10 had scoliosis less than 10 ° at skeletal maturity. Threshold 3: 50° surgery - The SRS website doesn't recommend surgery until 50°. The previous threshold was 40°, and it would be interesting to know the reasons for this change. I think between 40 ° and 50 ° there is a small place for bracing.

**Durmala.** delete „increased possibility of back pain and progression in adulthood „

**Grivas**. We need to discuss what is a failure after surgical and what is a failure after non-surgical treatment. These two are not the same

**Hresko**. I like this partition

**Maruyama**. 45 deg for surgical threshold also according to the Richards’ paper

**O'Brien**. SRS bracing criteria etc establishes 45 degrees as accepted surgical criteria…….are you suggesting changing this to 50 degrees match the Braist criteria?

**Price**.I have concerns with 30 degree Cobb angle

**Rigo**. Total agreement about the first point. I agree about a threshold value to define mild and moderate scoliosis and 30º is the most commonly used, but may be it should be applied according to the curve pattern (may be for lumbar would be better 25º). Surgical threshold is an unacceptable concept to my opinion. The decision about surgery should be based on individual aspects, the Cobb angle being just one of the aspects. I agree about using this as a ‘term’ but not with the implicit value telling ‘you must undergo surgery if you have 51º and not if you have 49º’... I think the word ‘threshold’ means exactly this...so perhaps another word her would be desirable ? To me the Threshold of 30º is not so much arbitrary as it is based on some external evidence but by itself is not really defining the quality of life further in life in an individual way. Other factors would define this better, probably. Pedagogically, a correct message would be that the angle by itself does not define the quality of life individually. If pedagogy goes in the sense of having more than 30º ‘you are in a higher risk for pain’ as far as ‘fairs about pain’ could be considered a factor predicting pain (no evidence to my knowledge but at least this is my experience), then we are not right. I think that ‘preventing about pain’ should not be more a ‘reason for treatment’. Pain is something to be treated but ‘preventing about pain’?? is a ‘boarding pass’ to ‘suffer pain’ (of course just an opinion). I added also some comments about 50º as a surgical threshold.

**Zaina**. We will have different groups since research will include low degree curves. So we could add more cathegories, 10-20°, 20-30° and so on

## Discussion 4

There is strong discussion about the importance of other parameters beyond Cobb degrees. Moreover it is clearly stated that Cobb angles are not a good reference for scoliosis treatment. Some questions are raised about the meaning of this recommendation. It is underlined that these thresholds (particularly below 30°) should not be used for treatment decisions. The 10° thresholds for definition of scoliosis is strongly questioned. The 30° threshold for possible problems in adulthood is questioned. A threshold for surgery is questioned and different limits are proposed. Overall, radiographic exams are regarded with a clinical eye, and consequently as very reductive.

Aulisa Lorenzo 10° is still a too low threshold for the beginning of the treatment. (x-rays standard error +/- 5°). This threshold can lead to overtreatment (start too early).

Aulisa Angelo Gabriele Report the results based only on Cobb degrees is a limit, we should considered also the rotation because 30 degrees with low rotation have a risk of progression lesser than one with higher rotation

Bernard Jean-Claude the cobb angle is not sufficient to explain the scoliosis and its treatment

Białek Marianna it depends on the individual approach to the patient

Brox Jens Ivar patients with scoliosis < 20 degrees should only be treated after > 5 degrees curve progression is documented and in studies, the association between back pain and 30 degrees is weak and could not be used for this purpose, 45-50 degrees for surgery

Chou Chungwai patients with scoliosis, regardless age or curvature severity, should take an X ray at least once a year.

De Seze Mathieu keep up the increased possibility of back pain and progression in adulthood

Diers Helmut X-ray based Cobb angles do not explain correctly thresholds. 3D parameter and rotation value will be neccessary.

Espinoza Pamela Yes, but I agree with Rigo's opinion.

Kluszczyński Marc Developments of new rehabilitation method allow to consider increasing surgical threshold to 60°, especially among patient till 15 years. We observe that maintenance of bracing after puberty growth results in improvement of scoliosis. This fact allows us to discuss this recommendation.

Neuhaus Sulam Lior I do not understand the intention or the meaning of this recommendation please send me an explanation - I feel it is very important

Neuhous Tamar I do not understand the intention or the meaning of this recommendation.

Parent Eric The 5 degree criteria is only relevant for follow-up to skeletal maturity. We are debating the clinical significance of certain radiographic changes over periods of less than the skeletal maturity. We should be providing guidance for trials of different durations. Using Semi-automated measurement techniques smaller measurement errors can be obtained for the Cobb measurements. I would favour adoption of the SRS bracing paper by Richards for studies till skeletal maturity but recommend we also adopt recommendations for shorter follow ups. For example Sanders report a risk of progression varying between 0.2 degrees per months to 1.6 degrees or so if I recall correctly . This could be used to establish standard to judge success at different time point. Depending on the risk of progression of the group of patients included.

Price Nigel 10 degrees is too low in my opinion to consider for treatment

Stikeleather Luke Remove the wording " surgical threshold" for curves of 50 degrees. It is a mistake to categorize all curves of this magnitude in this way. see M Rigo comments.

Talwalkar Vishwas I don't think the comment after the 30 degree threshold is always correct

Tassone Channing the inclusion of and commentary on the 30 degree threshold is still concerning to me as it will be interpreted in the lay community and literature

Tomasz Karski important new clinical observations / new symptoms connected with pelvis, with hips, with hips movements, with early rotation deformity of spine

Voets Helma 35 degrees instead of 30 degrees depending on growth 45 degrees in growth 50 degrees after growth

Wajchenberg Marcelo Its very important to consider the rotation of the trunk. I completly disagree to consider 10 degrees the definition of scoliosis. We know that the rotation is more important. So if the patient have 9 degrees with rotation, we do not consider scoliosis?

Wong M. S. 25 degree for bracing

## Suggestions for new versions

Aulisa Lorenzo I don't answer because the measure of 10° to define a scoliosis is not reliable

Brox Jens Ivar I do not agree in any of the terms because they are not supported by high quality evidence, therefore it is nonsense to answer question 28

Diers Helmut 3D parameter (lateral deviations and segmental rotations) are neccessary. Cobb is only 2-dimensional.

Marti Cindy Can you include the term "clinically significant terms" in version 1?

Neuhaus Sulam Lior the rank is not what I thing because I do not understand the recommendation

Tomasz Karski in discussion

Voets Helma the differences between the answers is not clear

Wajchenberg Marcelo I don't agree with any recomendation based in angulation! You have to include the rotation concept!

Recommendation 8. We recommend that radiographic research outcomes are presented in terms of number of patients improved (6° or more), unchanged (+/-5°) and progressed (6° or more) (C2)

## Results

| **Stage of Delphi Survey** | **Agreement** | | **Importance** | | |
| --- | --- | --- | --- | --- | --- |
|  | *Rating* | *%* | *Rating* | *Average* | *Median* |
| **3** | Complete | 100% | High | 4.00 | 4 |
| **4** | Good | 94.40% | High | 3.80 | 4 |

## Results of the Consensus

| **Versions voted** | **Percentage of preferences** | **Preferences in the Boards** |
| --- | --- | --- |
| We recommend that radiographic research outcomes are presented in terms of number of patients improved (6° or more), unchanged (+/-5°) and progressed (6° or more) | 50.0% | 68.8% |
| We recommend giving radiographic research outcomes mainly in clinically significant terms according to Recommendation 7. Radiographic results, if presented, must be given in terms of number of patients improved (6° or more), unchanged (+/-5°) and progressed (6° or more) | 50.0% | 31.3% |

## Discussion 3

The SRS criteria states 6° or more variations to be considered significant (Richards 2005). Moreover, the importance of this radiographic variation, that is based only on the measurement error but it is not clinically significant, could be reduced

Knott. Given that the standard error of measuring Cobb Angles is +/- 7 degrees, an improvement or worsening of only 5 degrees may not be a real change. I can go along with the 5 degrees reporting limit, though.

Maruyama. Improved (6 deg or more), unchanged (+/-5 deg), progressed (6 deg or more), according to the Richards’ paper in 2005

Rigo. I would increase the range of stabilization (10º) based on the clinical relevance. 5º can be a significant radiological change but it is not so clear that it represents a clinical relevant change (no matter in the direction of progression or regression)

Stikeleather. Additionally,_Defining brace/treatment failure by progression of more than 5 degrees should be reevaluated. A 25 degree curve on a 7 year old that finishes treatment at age 14 with a 35 degree curve should not be considered a failure

## Discussion 4

These criteria are widely questioned for their clinical low value. New technologies should allow a reduced measurement error for the same x-ray, even if it is known that postural changes are even more important than these 5° changes. The concept of minimally clinical important difference is raised beyond that of measurement error. Overall, radiographic exams are regarded with a clinical eye, and consequently as very reductive.

Aulisa Lorenzo What's the difference between 5 and 6 degrees?

Espinoza Pamela I agree with Dr. Rigo's opinion.

Hennes Axel in modern radiographic devices the measurement error is 2.3. The recommendation should take this in consideration

Kluszczyński Marc This recommendation is artificial, and it refers only to clinical research, and it is not relevant to common recommendations.

Knott Patrick Increase threshold for improvement to 10 degrees or more. Increase threshold for progression to 10 degrees or more.

Marti Cindy what is radiological margin of error, and significance, in Raimondi and coronal imbalance? Can these be included so we are not isolated only to Cobb?

Parent Eric The 5 degree criteria is only relevant for follow-up to skeletal maturity. We are debating the clinical significance of certain radiographic changes over periods of less than the skeletal maturity. We should be providing guidance for trials of different durations. Using Semi-automated measurement techniques smaller measurement errors can be obtained for the Cobb measurements. I would favour adoption of the SRS bracing paper by Richards for studies till skeletal maturity but recommend we also adopt recommendations for shorter follow ups. For example Sanders report a risk of progression varying between 0.2 degrees per months to 1.6 degrees or so if I recall correctly . This could be used to establish standard to judge success at different time point. Depending on the risk of progression of the group of patients included.

Sanchez Judith I.m agree with stikeleather

Tassone Channing method of obtaining radiographs in a standardized way will be critical to legitimize the data

Tomasz Karski In proper (new) causal prophylaxis never progression

Van Loon Piet A revisitation of value of this numbers is necessary, because all functional , and even so important items are not mentioned anywhere.

Wajchenberg Marcelo to correlate with clinical evaluation. The patient is not a angle or a number

## Suggestions for new versions

Bernard Jean-Claude I think the balance of the spine is more important that cobb

Bettany-Saltikov Josette version 3 is not very clear. should read i would suggest a full stop after version 3 after more and then to write " We do not recommend that results are presented as averages. personally i think both should be presented.

Brox Jens Ivar Of course average and SD should be reported in addition

Deceuninck Julie This recommendation have to include 3D parameters and not only frontal Cobb angle

Tomasz Karski in discussion

Voets Helma marginal differences between the answers

Recommendation 9. We recommend the adoption of the “Risser+” staging. This is the result of the confluence between the original US Risser staging, and the so-called European version of Risser staging as modified by Stagnara [10-12]. It has been added also the tryradiate cartilage fusion, that has been shown to be an important and prognostic subdivision of Risser staging 0. (D2)

| **“Risser+” staging** | **Tryradiate cartilage ossification** | **US Risser staging** | **European Risser staging** |
| --- | --- | --- | --- |
| **0-** | No | 0 | 0 |
| **0** | Yes | 0 | 0 |
| **1**  **0-25% coverage** |  | 1  0-25% coverage | 1  initial ossification |
| **2**  **25-50% coverage** |  | 2  25-50% coverage | 2  partial coverage |
| **3**  **50-75% coverage** |  | 3  50-75% coverage |  |
| **3/4**  **75-100% coverage** |  | 4  75-100% coverage | 3  complete coverage |
| **4**  **start of fusion** |  |  | 4  start of fusion |
| **5**  **complete fusion** |  | 5  complete fusion | 5  complete fusion |

## Results

| **Stage of Delphi Survey** | **Agreement** | | **Importance** | | |
| --- | --- | --- | --- | --- | --- |
|  | *Rating* | *%* | *Rating* | *Average* | *Median* |
| **3** | Strong | 93.0% | High | 3.50 | 4 |
| **4** | Weak | 86.18% | High | 3.69 | 4 |

## Results of the Consensus

Which staging do you prefer ?

| **Versions voted** | **Percentage of preferences** |
| --- | --- |
| 0-, 0, 1, 2, 3, 3/4,4,5 | 41.7% |
| 0 open, 0 closed, 1, 2, 3, 4, 4+, 5 | 30.6% |
| 0-, 0+, 1, 2, 3-, 3+,4,5 | 27.8% |
| 0a, 0b, 1, 2, 3a, 3b, 4, 5 | 0% |

Do you like maintaining the name Risser or you prefer to avoid it ? Chose the preferred name:

| **Versions voted** | **Percentage of preferences** |
| --- | --- |
| Risser+ staging | 61.1% |
| SOSORT-SRS staging | 38.9% |

Would you like to add this sentence to the recommendation “New additional bone maturation parameters coming from the same x-rays should be searched with high quality studies to improve in the future the SOSORT-SRS Risser+ staging” ?

| **Versions voted** | **Percentage of preferences** |
| --- | --- |
| Yes | 69.4% |
| No | 30.6% |

## Discussion 1-2

Price. Future studies need to break out Risser 0, triradiate cartilge open ( very high risk) from the Risser 2, also Risser classification of Risser 2 is very unreliable and here I agree with European partial coverage.

Negrini I agree on this point: in fact we could introduce this concept in the text. Nevertheless, we run the risk to enter a little too much into the details if we considered that the aim is to offer a general scheme.

Price. This may be an opportunity to start using elbow ulnar apophysis and hand age criteria.

Negrini. The same as above for triradiate cartilage. The other problem here is that elbow ulnar apophysis, as far as I know, has been proposed by Dimeglio but it is not validated. Am I wrong ? Moreover, it is not visible in most of cases in the regular x-rays, while , in the new proposed positioning of the hands for the EOS system it is not visible by definition.

Hresko. “European Risser 4” is probably a radiological artefact due to superposition of the secondary ossification centre of the iliac crest with the shadow of the posterior part of the iliac bone (Eur Spine J 2008; 17: 1676-85). For me, no need to promote this parameter. We should better stay simple: Risser zero, Risser negative (0,1,2), Risser positive (3,4,5) "

Channing Tassone. It is better to clearly name the stages of Risser: SRS and SOSORT must make an agreement on the RISSER stage (Instead of the European or American Risser sign we will have a universally agreed Risser sign (The SOSORT- SRS Risser sign)

Grivas. It is better to clearly name the stages of Risser: SRS and SOSORT must make an agreement on the RISSER stage (Instead of the European or American Risser sign we will have a universally agreed Risser sign (The SOSORT- SRS Risser sign)

Stikeleather. Is it too big a task to create a universally accepted standard? Now that SRS /SOSORT are international organizations can we propose discussion on this topic?

Akin Ugras. I am agreed with Dr Hresko to simplify terms, Risser negative for open triradiate cartilage, and simple American staging

Bettany Saltikov. I agree with Luke here

De Mauroy. I don’t use Risser staging, because ossification of the iliac crest seems modified by the pressure of the Lyon brace on the iliac crest. The P point of Duval-Beaupère corresponds to the beginning of the pubertal growth. The least poor indicator of bone mass seems to be the standing or sitting height. For us, the end of the growth in height serves to define the protocol of brace weaning.

## Discussion 3

Akin Ugras. US Risser staging, to clarify open triradiate cartilage only use Risser negative

Hresko. I like the title ”Risser + staging” but I would change the name of 0 to “Risser 0 open” as triradiate cartilage open and Risser 0 closed . I would also eliminate Risser 3a and 3b to just have riser 3 50-100% coverage

Grivas. we have also to descuse it taking in account Tomasz’s publication on this topic

Kotwicki. OK for triradiate cartilage as 0- or 0+. I opt for US Risser completed with 4+ or 4.5 value for “start of fusion”

Maruyama. In the SOSORT-SRS staging, subdivision of Risser3 to 3a and 3b is innecessary.

Price. Let’s come up with universal Risser score

Negrini. If we want to convert to one single Risser+ staging the actual US and EU Risser stagings we need to be inclusive, and not simply expand the US Risser sign without taking into any account the EU Risser. We can change the names used for the various Risser stages to make them more easy to be remembered.

## Discussion 4

People out of Europe prefer to stick with the original Risser classification, while Europeans and SOSORT members recognise the need for a fusion of the two classifications. It is suggested to avoid a third Risser staging, since there are already 2. Other radiographic criteria are suggested, even if should be recognised that most of the times they are not in the same x-ray (particularly with new EOS system) or have not been studied thoroughly. Limits of this staging are well known and raised again.

De Seze Mathieu the stage risser +0a could be subdivise with the dimeglio's criteria on elbow which semms indicated the begining of the quick spine growth before the ossification of the tryradiate cartilage moreover the risser 3, 4 and 5 could be completed by the tranchanter ossification identifying the end of the quick spine growth

Espinoza Pamela I agree with the inclusion idea of Negrini and no simply expand the US Risser sign without taking account the EU Risser.

Fabris Monterumici Daniele fusion happen late after end of spine growth. Risser 4 and 5 are late after growth completed

Hennes Axel highly sophisticated, difficult to see on most of the x-ray films

Kerstholt Janine For medical scientists maybe important, for physical therapists not. spine stability is more important that Risser stage.

Parent Eric I am reluctant to introduce a third grading with the name RISSER as we may significantly create more confusion to an area where there is a fair bit of confusion. I would suggest if the need for a modification is essential that we do not use the term RISSER at all so that It be clear that we have a different scoring endorsed by the Societies I would agree with the proposal and would try to simplify by lumping together both Risser 3. PS was there discussion about recommending threshold for inclusion in trials? Reviewers have pushed hards for us to use maturity as a selection criteria.

Price Nigel should try to consolodate this if possibel

Tomasz Karski Risser test in NOT IMPORTANT - in new causal prophylaxis exist no "progresion"of scoliosis / curves / stiffness / rib hump. But start of causal exercises shuold be in age 2 - 3 years - in time of beginnig of every type of scoliosis. Thera are 3 types and 4 form of scoliosis. Namely: "S" scoliosis in I epg group - stiff spine two curves, progression. Causal influences "gait" and standing on the right leg. 2/ "C" scoliosis II A epg - felxible spine, one curve L - left convex, causal influences "only standing on the right leg" permanetly. "S" II / B epg scoliosis Causal influences ""standing on the right leg and wrong exercises / laxity of jonts. "I" scoliosis III epg - scoliosis without scoliosis (or very small), with stiff spine. Causal influence - only walking / gait.

Ugras Akin Only states minus or plus for triradiate cartilage and not change for Risser

Wajchenberg Marcelo I prefer to mantain the original classification

## Suggestions for new versions

Brox Jens Ivar 0,1,2,3,4,5 is sufficient. The kappa values for classification should be determined in high quality pragmatic studies.

de Ru Esther do not feel I know enough to really judge properly

De Seze Mathieu i propose to complete the version by identification of the 3 points of ossifications: ossification of the second point of ossification in the elbow (dimeglio 0= open, C= clossed); triradié open and closed, and for the end of the growth trochanter ossification could be associated to risser to increaseitsreproducibility so version 9: risser (0,1,2,3,4,5); + dimeglio open or close; + triradiated open or close + trochanter open or close

Kotwicki Tomasz 0 open, 0 closed, 1, 2, 3, 4, 4+, 5

minnella salvatore 0-, 0+, 1, 2, 3-, 3+,4,5

Parent Eric see comment above. Avoid term RISSER. Stages: 0o, 0c, 1, 2, 3, 4, 5 A rationale for dividing grades 3 and 4 in terms of effect on prognostic when exposed to non op treatment is needed.

Satyawati Rwahita I choose to US Risser Staging because it's the only staging used in Indonesia; with addition of 4-5 value for start time of fusion. I agree with Kotwicki for addition of 0- and 0+ to determine the prepubertal periode

Tomasz Karski in discussion

Voets Helma Risser 0, 1, 2, 3, 4, 5

Recommendation 10. We recommend that radiographic research outcomes are presented also split in tables according to Cobb degrees at start of treatment (group of 5° Cobb) and bone age (Risser+ staging), like the following one (D1):

|  | **Early Onset** | | | | | **Juvenile** | **Adolescent** | | | | | | |
| --- | --- | --- | --- | --- | --- | --- | --- | --- | --- | --- | --- | --- | --- |
| *Age at start of treatment* | *0* | *1* | *2* | *3* | *4-5* | *6-9* | *10 or more* | | | | | | |
| *Risser+ staging* |  |  |  |  |  |  | *0-* | *0* | *1* | *2* | *3* | *3/4* | *4* |
| **Below 10°** (**with a rib hump / lumbar prominence)** |  |  |  |  |  |  |  |  |  |  |  |  |  |
| **11-19°** |  |  |  |  |  |  |  |  |  |  |  |  |  |
| **20-29°** |  |  |  |  |  |  |  |  |  |  |  |  |  |
| **30-39°** |  |  |  |  |  |  |  |  |  |  |  |  |  |
| **40-49°** |  |  |  |  |  |  |  |  |  |  |  |  |  |
| **50° or more** |  |  |  |  |  |  |  |  |  |  |  |  |  |

## Results

| **Stage of Delphi Survey** | **Agreement** | | **Importance** | | |
| --- | --- | --- | --- | --- | --- |
|  | *Rating* | *%* | *Rating* | *Average* | *Median* |
| **3** | Complete | 100% | High | 3.54 | 4 |
| **4** | Weak | 86.89% | High | 3.52 | 4 |

## Results of the Consensus

Which subdivision of the rows do you prefer ?

| **Versions voted** | **Percentage of preferences** |
| --- | --- |
| Every 10° like it is now | 86.1% |
| Every 5° | 13.9% |

For the first row “Below 10°”, what do you prefer ?

| **Versions voted** | **Percentage of preferences** |
| --- | --- |
| “Below 10° with a rib hump / lumbar prominence”, like it is now | 63.9% |
| “Below 10°” | 36.1% |

## Discussion 1-2: about classification according to aging

Price. Stefano-is this splitting of age groups a SOSORT consensus? I am familiar with the concept of early onset being less than 5 years-a concept the the Early Onset Scoliosis Group considers important-are the groups really heterogeneous?

Negrini. We re-introduced this classification in the SOSORT Guidelines. In reality, a classification should help for prognosis and treatment. In conservative, we face aggressive curves due to rapid growth: this is true until age 4, when growth slows down. In the past this classification was created, we could come out with a new one, but really in non-operative not splitting these groups could be a limitation. Nevertheless, this should be a point to be thoroughly discussed and we must maintain this point open to discussion. Tim proposed to join Infantile and Juvenile 1: the main difference here, to me, is that in the first year of age you can still have the self-resolving, that is not true afterwards. Really, I would like to know the opinion of the others as well before chosing.

Hresko: why not include Juvenile 1 with the infantile? To me, there is a big difference since in the first year of age you can still have the self-resolving, that is not true afterwards. Really, I would like to know the opinion of the others as well before chosing.

Hresko. Early onset scoliosis is a very common term used by SRS members and of great interest due to concern for chest growth, pulmonary function when fusion is perform in less than age 8. Can we use the term EOS but then subclassifiy it into EOS- infantile and EOS- juvenile 1( under age 7, too early for exercise approaches) and juvenile 2( old enough for exercise)? Again, to be discussed.

Channing Tassone. I do not find this very useful and I find it confusing, therefore the rest of the questionnaire must be constructed according to a more simple approach on the issue.

Grivas. I do not find this very useful and I find it confusing, therefore the rest of the questionnaire must be constructed according to a more simple approach on the issue.

Hresko. It would be best to be in agreement with the established EOS study groups that define EOS < age 5. I think there are too many sub groups. I would suggest EOS 0-5; Juvenile 1- age 5-7; juvenile 2- age 8-9; Adolescent > 10yr triradiate cartilage open and Risser 0; Risser 1-2; Risser 3-4-5; adult to menopause; all the rest the same but change term “flexed posture” to unbalanced sagittal posture or something else

Kotwicki. The proposal for Juvenile 1, 2, and 3 is right, however too complex for researchers to be followed. And you never knows exactly when the curve appeared, you only know when it was noticed – thus, such division of Juveniles refers more to quality of health care in early detection of scoliosis than to natural history of deforming spine.

Stikeleather. The term” early onset” seem to be well established ...but too broad. I like the idea of categorizing by age, as treatment may be very different and specific to these age groups. The SRS website still uses the infantile, juvenile, adolescent description under early onset. Can we use EOS and patient age with it? For example EOS 1 or EOS 7 or a range EOS 5-7.

Bettany-Saltikov- I like and agree with Lukes suggestion here.

De Mauroy. OK for early onset. Juvenile scoliosis can be connected to a growth rate of 5 cm per year. It becomes adolescent scoliosis when growth exceeds 5 cm per year till end of growth.

Rigo. I do not find the recommendation so complex or confusing. Personally I find it OK. Or perhaps I did not understand the graphic?.

Akin Ugras. We can use infantile form for self resolving type, and EOS term for under age 8, because pulmonary function is delinate the treatment plan.

## Discussion 1-2: about including patients below 11°

Channing Tassone. Curve >11 degrees to avoid unnecessary treatment

Hresko. There should be radiographic curve greater than 10 degree

Knott. I am concerned that we may continue to define smaller and smaller deformities for research groups. These lead everyone to believe that these curves need treatment, when they may not.

Kotwicki. Minimal curves analyzed in separate category

Stikeleather. What is the incidence of this? And do we need more discussion on it?

Bettany-Saltikov. I have come across a paper showing that having a slight thoracic kyphosis in adulthood is quite normal (Toshio Doi 2011- have attached paper to the email). Should we be considering this? Also all my back shape studies have indicated this ie a normal spine is not necessarily straight in fact quite the opposite seems to be true within certain limits of course.

De Mauroy. Below 10° is a control group

Rigo. Yes, absolutely. To my opinion the concept scoliosis should be re-defined and not only based on a particular Cobb angle. Other thing is about when to recommend follow-up using radiographs, and how to avoid over-treatment. Thus, I agree with Kotwicki about a separate category.

## Discussion 3

Price. experience tells me that splitting into these groups is probably not clinically important

De Mauroy. For juvenile: Growth near 5cm per year. For adolescent: after Juvenile and till end of growth

Kotwicki. For juvenile: Growth near 5cm per year. For adolescent: after Juvenile and till end of growth

Zaina. I suggest groups of 10°

Negrini. Perhaps this subdivision in 5° groups is too narrow for clinical usage. Moreover, adding “with a rib hump / lumbar prominence” to “Below 10°” could be a good idea

## Discussion 4

The issue of complication is the most raised. Moreover, the issue of curves above 50 is raised as well.

Bettany-Saltikov Josette it seems a little complicated

Brox Jens Ivar We found that juvenile behave similar to adolescent and can actually be grouped together

Dr Fodor Janosné go up to 60 degrees, because of the 7 degree measuring error.

Grivas Theodoros to complicated

Kerstholt Janine Is'nt the specification lumbar or thoracic and with or without hump is clinical more important than this narrow specification?

Lebel Andrea We need a row for 50-55 and 55-60 and 65+ some patients refuse surgery and while waiting to make a decision would be treated to halt progression. This data could be useful as well.

Parent Eric There are too many categories. With the current evidence for prognostic under different treatments we should divide into bigger bins. Match Sosort guidelines where recommendations differ?

Price Nigel too many categories, should consolidate

Rivett LouAnn one

Satyawati Rwahita observing our client's condition, we suggest to observe patients with Cobb's angle below 10 degree as there will be correlation with growth/height problems and treat them when there's potential of progression ( both from x-ray evaluation and body height)

Simony Ane we rarely use the risser classification in the nordic countries, so if demanded we would not be able to present our findings.

Tomasz Karski Infantile scoliosis is NOT idiopathic scoliosis. Cobb angle is not important. Clinical symptoms are important.

Voets Helma below 10 degrees between 10 and 25 degrees between 25 en 45 degrees during growth 45 degrees or more after growth 50 degrees or more without pain severe pain can be a reason for surgical treatment

Wajchenberg Marcelo 4 degrees is a small interval

## Suggestions for new versions

Aulisa Lorenzo 10° is no t to be considered as a threshold for starting the treatment

Brox Jens Ivar Does not make sense to me, < 10 degrees should not be classified as scoliosis

Dr Fodor Janosné 50- 55, 55-60

Marti Cindy IF we are to add clinical element to table, such as rib hump or lumbar prominence, i would strongly support adding Rigo's "modified Adams' Test" that looks for a regional area of hypokyphosis in sagittal plane forward bend test

Tomasz Karski In discussion

Recommendation 11. We recommend that standardised and validated questionnaires are used to report Quality of Life results (B2)

## Results

| **Stage of Delphi Survey** | **Agreement** | | **Importance** | | |
| --- | --- | --- | --- | --- | --- |
|  | *Rating* | *%* | *Rating* | *Average* | *Median* |
| **3** | Complete | 100% | High | 3.92 | 4 |
| **4** | High | 97.54% | High | 3.92 | 4 |

## Discussion 3

Akin Ugras. Prefer to use SRS questionnaire

Bettany-Saltikov. Questionnaires have been shown to have a number of limitations such as cognitive dissonance for instance. There are numerous papers basing conclusions on quality of life questionnaires, mainly the SRS-22, claiming a high patient satisfaction after scoliosis surgery (Kepler 2012) and poss; however, the results or conclusions derived from these studies are questionable when the ‘dissonance’ effect is considered, as referenced in a discussion of post-surgical interviews (Moramarco 2013). Cognitive dissonance occurs most often in situations where an individual must choose between two incompatible beliefs or actions and there is a tendency for individuals to seek consistency among their cognitions. Unable to face an inconsistency, such as being dissatisfied with a surgical procedure or following the wearing of a brace for many years, a person will often change his/her attitude. Surgery and /or having worn a brace for many years is impossible to reverse, but subjective beliefs and attitudes can be altered more easily. As a result, a patient not satisfied with a surgical procedure or having worn A brace for many years may not necessarily admit this

Hresko. Yes- but we need a improved HRQoL scale as present SRS score is not appropriate as ceiling effect

Negrini. In such a recommendation we cannot suggest specific questionnaires, since today there are no proofs of superiority of any of these above the others. Specifically, the SRS-22 has shown a lot of limits for clinical usage, particularly in conservative settings.

## Discussion 4

Bettany-Saltikov Josette are there quality of life questionnaires specifically for PSSE?

Espinoza Pamela I like SRS 22

Grivas Theodoros The proper questionnaire for non-op treatment is the BrQ

Kerstholt Janine make standard questionnaires for orthopedists and physical therapist for scientific use in SOSORT

Kluszczyński Marc Questionnaires Quality of Life should be used to assess the condition of patients after surgical interventions and adult patients.

Marti Cindy Even if tests are standardized and validated, i recommend there is a test that is used that is valid in the absence of pain since so many AIS do not have pain. Can there be 2 QOL tests...one for pts wiht pain, one for without? Is there some way to account for short term reduced QOL that sometimes happens in early phase of Rehab due to stress of appointments and bracing intervention? IS there QOl taht can ask not only how pt feels about themselves short term / "Corrent" but reflection on how they feel about what they are learning / doing for long term?

Monroe Marcia Interesting questionnaires questions and accessible to understand to the reader.

Parent Eric Maybe we need to recommend adoption of a global rating of change as well. The QOL tools do not perform well in small curves.

Sanchez Judith I,m agree with Negrini, but the SRS is the more extended questionnaire

Tomasz Karski In discussion

Van Loon Piet more functional questions. Amount of bending, hand-floor. PAin and localisation. fatigue, leg symptoms like weakness or postural weakness

## Suggestions for new versions

Bettany-Saltikov Josette i think it is important to clarify when using these validated questionnaires whether they were developed for surgical patients braced patients or for patients having PSSE

Tomasz Karski In discussion

Marti Cindy We will use non-pain QOL questionnaires for patients that are admitted without pain.

Previous recommendation 10 is rejected

## Text rejected

We recommend that patients are split into two groups: previously treated and not treated. We recommend not to consider as a previous treatments any approach without proof of efficacy in the literature

## Results

This recommendation comes from the fusion of two previous recommendations, whose results are reported below

| Stage of Delphi Survey | **Agreement** | | **Importance** | | |
| --- | --- | --- | --- | --- | --- |
|  | *Rating* | *%* | *Rating* | *Average* | *Median* |
| **4** | No | 76.86% | Medium | 3.37 | 4 |

## Discussion 4

The main issue with this recommendation is the identification of really effective treatments. Also the dangerous treatments issues (bad exercises and/or bracing, but also highly mobilizing activities) is raised. All these things cannot be controlled. In the meantime the issue of patients already treated with effective treatments remains.

Bettany-Saltikov Josette if treated with a previous treatment with no proof of efficacy to write down what this was

Cohen Larry there is too much dispute what is considered previous effective treatment

Czaprowski Dariusz I`m agree that a previous treatment without proof of efficacy should not be taken into account. However, we should be aware that it may modify the treatment

Durmala Jacek with information which treatment method were conducted

Kerstholt Janine It depends of with treatment. don’t mix PSSE with other kinds of treatments.

Marcotte Louise I do not think we should not consider any other treatment without proof. All other types of treatment should be taken into consideration.

Marti Cindy "past treatment' can include so many things...including things that can harm pts like yoga for scoliosis, or poor exercise advice, etc. Also there is a general lack of proof of efficacy in the literature, Cochrane or not...so it seems a moot point to say it. Could you say groups 1) untreated 2) previous treatment with brace, no ex 3) previous treatment with scoliosis specific ex 4) previous treatment with non-scoliosis specific ex?

Neuhaus Sulam Lior I recommend to include a group of previous not proof treatment.

Parent Eric I believe instead we should emphasize that co-interventions be reported and possibly accounted for in statistical analysis. we could set a threshold for when results be reported separately. EG if more than 20% are treated with co-intervention or previous interventions.

Pizzetti Paolo For some treatment proof of efficacy could not be available yet.

Price Nigel do not think this matters

Roig Oliver Maria Magdalena I think it is interesting the existence of this two groups, although it may be difficult to establish which are the approaches with proof efficacy/evidence in the literature due to lack of information.

Sanchez Judith I,m agree with Dr Rigo

Stoliński Łukasz I think we should consider a previous treatment even if there was approach without proof, but we must write this information. So we can divide this previous treatment for: with proof and without proof in the literature. Child could do some specific exercises, which can be important in the therapy, but it can be some basic method. So we can prepare 3 groups - 1.without treatment, 2.with approach with proof, 3.with approach without proof

Tomasz Karski In dscussion

Voets Helma growth potention is more important

## Suggestions for new versions

Bettany-Saltikov Josette i just wanted to let you know that the Cochrane is now accepting non randomised studies so long as they are prospective and with a control group. there is actually an on going rCT of exercises by Parent in Canada and another in the Uk.

Betz Joseph literature requirements are too restrictive

Brox Jens Ivar Does not make sense to me

Hresko Timothy You have left out group that may have had selective surgical fusion of thorax and brace/exercise for lumbar curve

Kerstholt Janine It depends of witch brace. there are good working and (hardly) not working braces.

Marcotte Louise We recommend that patients are split into 2 groups: previously treated and not treated. All treatment aimed specifically at helping the patient with his scoliosis should be noted.

Neuhous Tamar I suggest to include one group treated previously and one group not treated previously

Parent Eric We need to clarify if authors will then report only split results or it this reporting to be done in addition of combined results. We should suggest that all non operative research data be deposited in a repository to allow future metanalyses

Satyawati Rwahita split the previously treatments into 3 groups: 1. orthopaedic treatment; 2. rehabilitative treatment (exercise and or bracing); 3. other treatment

Tomasz Karski In discussion

## *Previous recommendation 10*: We recommend that patients are split into two groups: previously treated and not treated

## Results

| **Stage of Delphi Survey** | **Agreement** | | **Importance** | | |
| --- | --- | --- | --- | --- | --- |
|  | *Rating* | *%* | *Rating* | *Average* | *Median* |
| **3** | Weak | 83% | Medium | 3.20 | 3 |

## Discussion3

Bettany-Saltikov. 3 groups: 1 No brace, no exercise 2 Other brace –specify 3 PSSE Exercise. We need to distinguish between PSSE exercises and other types of spinal exercises

De Mauroy. 3 groups: 1 No brace, no exercise; 2 Other brace; 3 Exercise

Durmala. with information_which treatmend method were conducted

O'Brien. specify what previous treatment was used

Price. still not sure of clinical relevance

Rigo. Some previous treatments could be considered irrelevant but, how to decide this? If the researcher decides: relevant or irrelevant, is this a bias? In any case the concept treatment should be well defined to allow researchers to split.

Negrini. The concept of what could be considered of any efficacy should be faced and stated,

## *Previous recommendation 11*: We recommend not to consider as a previous treatments any approach without proof of efficacy in the literature

## Results

| **Stage of Delphi Survey** | **Agreement** | | **Importance** | | |
| --- | --- | --- | --- | --- | --- |
|  | *Rating* | *%* | *Rating* | *Average* | *Median* |
| **3** | Weak | 87% | Very high | 3.77 | 4 |

## Discussion 3

Hresko. At the present time , that would exclude all treatments except bracing

O'Brien. what standard criterion is used to establish proof of efficacy in the literature?

Price. proof of efficacy and effectiveness are in health literature two different concepts-which to we mean?

Rigo. OK, this is more or less answering about my previous concern. But still some concerns about this point. In fact bracing works but many patients attending consultation with a previous brace are doing like non-treated due to the very poor quality of the brace. In any case, I agree about splitting and about offering a definition of treated and not treated, which could be revised periodically.

Zaina. what about exercise?? We should give a clear indication about this

Negrini. The concept of what could be considered of any efficacy should be faced, Perhaps also changing efficacy to evidence is better

## *Actual recommendation 10*: We recommend that patients are split into two groups: previously treated and not treated. We recommend not to consider as a previous treatments any approach without proof of efficacy in the literature

Recommendation 12. We recommend in clinical research to include data on adherence to treatment: statistical analysis should include these data. Prospective bracing studies must use objective means to monitor adherence. Exercises studies must report data on adherence to number and length of assisted sessions, and home-exercise. (B2)

## Results

| **Stage of Delphi Survey** | **Agreement** | | **Importance** | | |
| --- | --- | --- | --- | --- | --- |
|  | *Rating* | *%* | *Rating* | *Average* | *Median* |
| **3** | Complete | 100% | High | 3.93 | 4 |
| **4** | High | 97.48% | High | 3.93 | 4 |

## Results of the Consensus

| **Versions voted** | **Percentage of preferences** |
| --- | --- |
| We recommend in clinical research to include data on adherence to treatment: statistical analysis should include these data. Prospective bracing studies must use objective means to monitor adherence. Exercises studies must report data on adherence to number and length of assisted sessions, and home-exercise. | 69.4% |
| We recommend in clinical research to include data on adherence to treatment, possibly obtained through objective means: statistical analysis should include these data. | 30.6% |

## Discussion 2

Hresko. I would suggest we be more specific to request that brace treatment to include a compliance monitor. But what is compliance for exercises ??

O'Brien. think recommendation should be stronger for objective means especially within braces

Price. Very important to start incorporating compliance data into this and i think this should eventually be standard of measurement of brace and possibly other non op treatments

Rigo. Self reported should be enough. It is better ‘objective’ but not always possible.

Negrini. Unfortunately with exercises there are today no means to evaluate compliance. Moreover in retrospective studies it is not possible in most of the cases to have data about compliance other than subjective (and in many cases not even those). New versions proposed.

## Discussion 3

The problems of monitoring exercises, and of use of other monitoring means for bracing are raised, as well as statistical issues.

Berto Sofia Use the word adherence instead of compliance. It´s more an agreement between the patient and the health carer. Compliance means to take an order.

Bettany-Saltikov Josette i suggest to ask authors to write down the actual details of how they obtained the compliance data

Parent Eric We could recommend different compliance thresholds for requesting per protocol analyses. The compliance data can also be used as an adjustment variable in the statistical analysis. Tracking compliance is essential. Splitting results i am not so sure. We should emphasize the need to report intent-to treat analyses. Discourage reporting of per protocol analysis alone.

Tomasz Karski In discussion

Wynne James monitor must be validated

## Suggestions for new versions

Aulisa Lorenzo Objective means must include questionnaires also

Parent Eric For Exercises I would like to see us recommend that number of prescribed session attended be reported and that the number of self-reported days where prescribed exercises dose was completed be reported (with possibly recommending routine oversight but parents? In a self-report note book.

Tomasz Karski In dscussion

Recommendation 13. In the introduction of a new non-operative treatment for patients during growth, for the radiographic outcome we recommend that the following research steps are followed: (B2)

| **Type of result** | **Data analysed** |
| --- | --- |
| **Very short term** | In-brace correction |
| **Short term** | At least 12 months of treatment |
| **End of bone growth** | Risser+ 3/4 |
| **End of treatment** | At treatment discontinuation |
| **Final results at full bone maturity** | Risser 5 and/or ringapophysis closed  Minimum 1 year after end of treatment |
| **Follow-ups** | To be calculated from final results |

## Results

| **Stage of Delphi Survey** | **Agreement** | | **Importance** | | |
| --- | --- | --- | --- | --- | --- |
|  | *Rating* | *%* | *Rating* | *Average* | *Median* |
| **3** | Complete | 100% | High | 3.85 | 4 |
| **4** | High | 95.73% | High | 3.85 | 4 |

## Results of the Consensus

Which Risser 3 should be considered as end of growth ?

| **Versions voted** | **Percentage of preferences** |
| --- | --- |
| Risser+ 3/4 (corresponding to US Risser 3 and EU Risser 4): 100% coverage | 94.4% |
| Risser+ 3 (corresponding to US Risser 3 and EU Risser 2): 75% coverage | 5.6% |

## Discussion 3

Bettany-Saltikov. The above table only refers to the brace and not to PSSE. Another table needs to be developed for PSSE

Grivas. I am in line with Stikeleather

Rigo. I do not see the value of the short term.

Stikeleather. The wording of this question may need clarifying “When research is conducted on a new brace/ non-operative approach/ technique ,while the patient is growing, we recommend that the following steps are followed:”

Zaina. For short term at least 1 year, 6 months it’s not enough

Negrini. The table has already been corrected to expand to all treatments and not only bracing. Here are some different versions of the table to be voted.

## Discussion

The issues raised include: how to check and define short term results of exercises; how to define End of treatment; the usefulness of 1 versus 2 years after end of treatment.

Bettany-Saltikov Josette none if you mention braces then you should also mention PSSE you could also have during the PSSE

Brox Jens Ivar Baseline - in brace correction, short term, end of treatment or growth, follow-ups

Hresko Timothy Sanders digital staging 7 as end of growth instead of ring apophysis

Marcotte Louise Final results: at least 2 years after discontinuation

Parent Eric The table refers to discontinuation of treatment independent of skeletal maturity. This should be clarified. For example a 9 month exercise program ending before RISSER 3 would appear to be after medium term in the table but in fact would be ending before the medium term as defined by a Risser 3+ I believe adequately powered short term studies may be necessary proofs of concept. A student in the short term not providing any postulated positive effects may not justify long term investigation.

Stępień Agnieszka Why "Very short term" is proposed only for bracing? Sometimes we can achieve effects (in life quality, pain reduction) also without brace in the period shorter than 4-6 months.

Tomasz Karski Look my explanation - we all in world shout check / control the "biomechanical aetiology of so called idiopathic scoliosis" and introduce in the world the causal prophylaxis

Voets Helma Bending X-rays in combination with X-ray with brace 1 month after start brace treatment to evaluate the effect of brace treatment

## Suggestions for new versions

Brox Jens Ivar Too detailed

Recommendation 14. We recommend in research on non-operative treatment this table, from the Oxford Centre for Evidence-Based Medicine 2011 Levels of Evidence ([www.cebm.net/index.aspx?o=5653](http://www.cebm.net/index.aspx?o=5653)) (B2)

| **Type of research** | **Treatment**  **benefits / harms** | **Diagnosis** | **Prognosis** | **Screening** |
| --- | --- | --- | --- | --- |
| ***The question*** | *Does this intervention*  *help / harm?* | *Is this diagnostic-monitoring test accurate?* | *What will happen if we do not add a therapy?* | *Is this early detection test worthwhile?* |
| **Level I** | Systematic review of RCTs | Systematic review of cross-sectional studies with consistently applied gold standard and blinding | Systematic review of inception cohort studies | Systematic review of RCTs |
| **Level II** | RCT  Prospective controlled cohort study | Cross-sectional study with consistently applied gold standard and blinding | Inception cohort study (patients enrolled at same stage of their disease) | RCT |
| **Level III** | Retrospective controlled cohort study  Follow-up study | Study of non-consecutive patients  Study without consistently applied gold standard | Cohort study  Control arm of RCT | Controlled cohort study  Follow-up study |
| **Level IV** | Case-series  Case-control study  Historically controlled study | Case-control studies  Poor or non-independent gold standard | Case-series  Case-control study  Poor quality prognostic cohort study | Case-series  Case-control study  Historically controlled study |
| **Level V** | Mechanism-based reasoning | Mechanism-based reasoning | --- | Mechanism-based reasoning |

## Results

| **Stage of Delphi Survey** | **Agreement** | | **Importance** | | |
| --- | --- | --- | --- | --- | --- |
|  | *Rating* | *%* | *Rating* | *Average* | *Median* |
| **3** | Good | 93% | High | 4.08 | 4 |
| **4** | High | 95.67% | High | 3.89 | 4 |

## Discussion 3

Bettany-Saltikov. I don’t see how a prospective comparative study and a retrospective study can be considered to be the same level of evidence. The Cochrane back group would not allow us to include retrospective studies as they consider them too biased and much more prone to sources of error. Retrospective studies are considered to have a much weaker research design leading to a much poorer quality research study. Whilst these studies can maintain the sequence of they are less expensive and of shorter duration (than prospective cohort) but require large sample sizes. Rare outcomes are difficult to study and there is much less control over subject selection and measurements. The risk for confounding variables is also very high in this type of research design. Within the current the hierarchy of evidence model prospective studies are considered to be a much more powerful research design than retrospective studies as they can establish the sequence (or temporal ordering) of events, assess several outcomes, and crucially researchers can have much more control over subject selection, measurement of variables and end points. The weaknesses include the requirement of large sample sizes. They can also be very expensive and time-consuming to conduct.. They are also impractical for rare outcomes and the risk for confounding is still present.

Stikeleather. Fine tune the wording

Zaina. I suggest to remove the expert opinion, since this would leave to anyone to use and propose any device without any data!! I think in this case expert opinion in no evidence

Negrini. Discussion considerations are perfectly correct. After the first round, this proposal was introduced from the table proposed by journal dealing with spinal disorders (for uniformity) but without adequate corrections. Anyway, we cannot base on the Cochrane criteria that are too strict (only RCTs). What presented here is the correct version coming from spinal disorders journals.

## Discussion 4

The problem of external reference sources is raised. All scales of evidence have some limits: the one proposed here comes from the other Spine Journals, that are an adaptation to Oxford levels of evidence. Moreover, the problem of difficulties in making research on exercises and bracing, as well as out of Universities and/or in small practices is raised. In this respect other designs like long term observational not controlled studies, or case studies, or clinical databases is raised.

Betz Joseph Level IV: Case series and multiple case reports

Brox Jens Ivar A prospective long-term study is not mentioned, does not need to be comparative

Cohen Larry RCT would be almost impossible to perform correctly. Aim for level II

de Ru Esther I am very sceptical of RCT, and all trails, we know not everything is published so for me even a case study is evidence

Henning Susan Small clinics, like mine, do not have large volumes of patients for large scale research studies. Nevertheless, we are implementing rehab programs to treat patients with scoliosis using techniques not yet recognized by the US medical community, ie: Schroth Method & Postural Restoration. We would like to share our experiences and findings with the Scoliosis community in a meaningful way, but do not have the resources for formal research projects.

Matthews Martin Add retrospective audits providing they use detailed framework databases to reduce researcher and commercial bias. The use of audits report on current practice and provide a route for further research.

Parent Eric I strongly disagree with introducing a new hierarchy for our research. I would want us to align with the well accepted hierarchy such as the Cochrane collaboration but my preference and what I teach is usually the Oxford levels of evidence revision 2 from 2011.

Tomasz Karski Is presented in other earlier points

Van Loon Piet More emphasis on the cultural external factors that causes deformation. It can be taken for granted, that a complex set of treatment modalities all add to success or failure.

Voets Helma if possible

## Suggestions for new versions

Parent Eric I would refer to the Cochrane foundation. or Oxford levels of evidence.

Tomasz Karski In discusion

Recommendation 15. In the introduction of a new brace, we recommend to focus research on the following SRS inclusion criteria [13]: above 10 years of age, Risser 0-2, curves 25-40° Cobb. (D2)

## Results

| **Stage of Delphi Survey** | **Agreement** | | **Importance** | | |
| --- | --- | --- | --- | --- | --- |
|  | *Rating* | *%* | *Rating* | *Average* | *Median* |
| **3** | Weak | 87.00% | High | 4.00 | 4 |
| **4** | Weak | 89.66% | High | 3.77 | 4 |

## Results of the Consensus

| **Versions voted** | **Percentage of preferences** |
| --- | --- |
| In the introduction of a new brace, we recommend to focus research on the following SRS inclusion criteria [13]: above 10 years of age, Risser 0-2, curves 25-40° Cobb. | 58.3% |
| In the introduction of a new brace, we recommend to focus research on the SRS inclusion criteria [13]: above 10 years of age, Risser 0-2, curves 25-40° Cobb, no previous treatment; for females: maximum 1 year post-menarche. | 27.8% |
| In the introduction of a new brace, we recommend to focus research on the following SRS inclusion criteria [13]: above 10 years of age, Risser 0-2, curves 25-40° Cobb, no previous treatment. | 13.9% |

## Discussion 3

De Mauroy. Is it possible to publish on the SOSORT website a pdf of the reference? In case of copyright problems, can the authors of consensus write a note for the website?

Durmala. Why SRS only and not SRS and SOSORT ?

Maruyama. Richards’ paper proposed the inclusion criteria for the brace study, not the indication of brace treatment.

Stikeleather. Include a summary of the SRS indications as not everyone will be familiar with them.

Negrini. According to the comments, probably the recommendation was not self-explaining. Moreover, in this document we changed some parameters that are part of the actual SRS criteria

## Discussion 4

The SRS indications are not well known and some points of them have already been discussed during SOSORT Meetings showing that they are not adequate. Moreover some points have been already considered in other recommendations of this set.

Bettany-Saltikov Josette i would recommend to specify what the SRS Indications are. it would also be good to include specifics of the actual strength and thickness of the brace material.

de Ru Esther but have not read this bit... i am presuming people know what they are talking about

De Seze Mathieu SRS criteria propose to focus on a population for studies but not to indicate or not a brace, moreover these criteria could induce a too late treatment?

Grivas Theodoros add the SOSORT recommendations

Kerstholt Janine and growth factors

Parent Eric List the indication provided by SRS. Refer to Richard's paper?

Tomasz Karski just presented

Van Loon Piet There is no attention to the form of non bracing treatment that should be followed. And it prevents the urge to get to real early diagnosis at schools, that can prevent bracing by other treatments like dedicated exercises, advises in lifestyle , like active sitting etc. The SRS do not differentiate for different countries, where complete different school screening, availability of excersise therapy and awareness under doctors can exist

## Suggestions for new versions

Betts Tony with or without previous treatment

Marti Cindy I think consideration should be given for curves at 20 deg in Risser 0. I don’t think Maximum 1 yr post menarche makes sense due to literature stating growth can continue past this. I think treatment should continue to Risser 4 not 3.

Parent Eric I would delete the reference to the end of treatment which could become another recommendation and the outcome which is already covered in another item.

Tomasz Karski In discussion

Van Loon Piet Focus on the existence of the thoracolumbar kyphosis and the presence of increasing neuromuscular tightness in growth spurts. The clinical part. In females good active bracing and intensive other measurements will give improvement till Risser IV

Recommendation 16. In presenting research results on bracing, we recommend to answer to the questionnaire in Appendix of the SOSORT Guidelines for Management of braced patients [14] to understand how team managed patients. (B3)

## Results

| **Stage of Delphi Survey** | **Agreement** | | **Importance** | | |
| --- | --- | --- | --- | --- | --- |
|  | *Rating* | *%* | *Rating* | *Average* | *Median* |
| **3** | Good | 93.00% | Medium | 3.20 | 3 |
| **4** | High | 95.65% | Medium | 3.48 | 4 |

## Results of the Consensus

| **Versions voted** | **Percentage of preferences** |
| --- | --- |
| In presenting research results on bracing, we recommend to answer to the questionnaire in Appendix of the SOSORT Guidelines for Management of braced patients [14] to understand how team managed patients | 57.6% |
| We recommend to state in clinical research studies if patients were managed by single professionals or by a team working together. The team work and professional composition should be explained. With this aim, in bracing studies we recommend to answer to the questionnaire in Appendix of the SOSORT Guidelines for Management of braced patients [14] to understand how team managed patients | 42.4% |

## Discussion 3

De Mauroy. Is it possible to publish on the SOSORT website a pdf of the reference? In case of copyright problems, can the authors of consensus write a note for the website?

Stikeleather. Editing this sentence will improve its clarity

## Discussion 4

The generalizability of this recommendation to exercises studies is raised.

Bettany-Saltikov Josette could we have a similar one for PSSE?

Henning Susan In my experience, once most adolescents have been given a brace, they are less motivated to work on scoliosis specific exercises. In this case, correction is passive. They do not develop the muscle control, positional self awareness and breathing ability necessary to maintain over their lifetime. Strong encouragement should be given by rehab team members regarding the crucial importance of continuing scoliosis specific therapeutic activities during the period of bracing, transitioning out of the brace and long term.

Parent Eric OR suggest that a study protocol complete with the info about the questionnaire items be published.

Stikeleather Luke the question/statement is a bit confusing and should be edited for clear meaning.

Tomasz Karski In discussion

## Suggestions for new versions

Bettany-Saltikov Josette could we add in presenting results for PSSE can you please state whether patients were only managed by a sole PT or managed as a MDT team?

Knott Patrick "to understand the importance of interprofessional team management."

Tomasz Karski In discussion

Recommendation 17. In presenting results on bracing, we recommend to specify results according to the dosage of bracing in terms of impact on patients’ social life. (B2)

| **Nightime** | In bed only |
| --- | --- |
| **Home-time** | At home only (up to 14h) |
| **Part-time** | At least half a day without the brace (15-18h) |
| **Full-time** | Less than half a day without the brace (19-22h) |
| **Total time** | Almost no pauses (23-24h). (B2) |

## Results

| **Stage of Delphi Survey** | **Agreement** | | **Importance** | | |
| --- | --- | --- | --- | --- | --- |
|  | *Rating* | *%* | *Rating* | *Average* | *Median* |
| **3** | No | 73% | High | 3.43 | 4 |
| **4** | High | 96.52% | High | 3.96 | 4 |

## Results of the Consensus

| **Versions voted** | **Percentage of preferences** |
| --- | --- |
| Like it is now: Night-time: in bed - Home-time: up to 14h – Part-time: 15-18h – Full-time: 19-22h - Total time: 23-24h | 54.5% |
| Night-time: in bed - Home-time: up to 16h – Part-time: 17-20h – Full-time: 21-24h | 27.3% |
| Night-time: up to 10h - Part-time 11-16h – Full-time is 17-24h | 18.2% |

## Discussion 3

Akin Ugras. Half time and total time are unnecessary

Bettany-Saltikov. Can we also do this in presenting results from studies on PSSE? I mean regarding dosage?

De Mauroy. Night time is 8h/24. Full time is 23h/24. Total time is 24h/24 (only 10’ for shower) like a plaster cast

Durmala. Nighttime 6-10. Home-time= Half daytime 10-18. Full time= Total time 19-21. In practice (by me) maximun dosage is 20 hours

Grivas. Night time should not be 0 but at least 6 to 8 hours

Hresko. Katz study implies that night brace wear did not improve results if patient wore for greater than 12 in day time. So consider to replace time segments to( a.)non compliant( less than 6 hour during the day) b. Partial day time ( 6 -10 hours) c, Full time day time_greater than 10 hours daytime (d) fulltime day time and night ( e) night time only

Kotwicki. Night time is 8h/24. Part time is 12h/24h = out of school. Full time is 20h/24 or more

O'Brien. Night time and home time would be same, wouldn’t it? Should use term Day Time?

Price. Not sure how the splitting helps... perhaps a threshold number would be more evidence based

Rigo. I think night time is ‘night time’ (brace only when lying) so hours is a not so important concept here. Home time could be defined as night plus all the hours at home, which could be considered until 16 hours. From 16 to 20 hours could be defined as a ‘partial time’ and over 20 H ‘full time’ with a subdivision here taking a part those really wearing the brace 23-24 H (from objective measurement). This division and definition of dosage looks easier to define from self reporting.

Stikeleather. Night time should be minimum of 6 to 8 hours, not zero. Maybe….”When presenting results on bracing, we recommend the dosage of wearing time be reported as follows”

Negrini. A classification of the number of hours of bracing is somehow desirable, but the discussion clearly shows how far we are from any agreement. Studies are very few and sparse. If we will be able to reach at least a low degree of agreement, we will introduce the recommendation otherwise we will avoid it. Below some different versions of the table are proposed for voting in the Meeting

## Discussion 4

It is proposed to reduce groups, but proposals are very different according to the basic ideas of each participant. Moreover, there are some pre-concepts according to own experience, such as that nighttime bracing is not effective, or that braces are not used more than 18-20 hours: studies exists stating different results. In any case recommendations have to be comprehensive, not to exclude future studies.

Aulisa Lorenzo Is a nighttime bracing still considered as a conservative treatment of scoliosis?

Brox Jens Ivar Does not make sense to go beyond 19 hours according to the recently published RCT

Durmala Jacek Nighttime 6-10. Home-time= Half daytime 10-18. Full time= Total time 19-21. In practice (by me) maximun dosage is 20 hours

Espinoza Pamela I agree with Bettany-Saltikov at this point too.

Fabris Monterumici Daniele less subgroups

Hennes Axel abolition of home-time

Henning Susan It would be important to me to distinguish whether the patient participated in an active therapeutic activity program as well, or whether the bracing was the sole treatment.

Marcotte Louise Except for night time which should only be during night time

Matthews Martin In neurological onset scoliosis it is common to not wear bracing at night time due to neurological proprioceptive reasons so we should also add day time up to 12 hours per day as an option as well.

Neuhaus Sulam Lior nighttime 10 hours per day, home time 11-16 hours per day, full time 19-23 hours per day

Papadopoulos Dimitris exclude nighttime - home time - total time

Parent Eric We should suggest deposition of outcome data with compliance data into a repository to allow future meta analysis. each category should be based on quantities known to be threshold of effectiveness in previous research. EG. night, vs 13h vs 18 hr vs 22-23+ I do not see the relevance of the distinction between home-time and half-daytime.

Price Nigel too many categories

Stikeleather Luke Simple is better! too many categories makes it more complicated.

Tomasz Karski Important "NEW PROPHYLAXIS"

Van Loon Piet give a lifestylescore for the other hours. If only hours are spent slumping and sloughing , the effect of the brace can be disappointing.

Voets Helma nightime: 12 hours per day full/total time: 19-23 hours

## Suggestions for new versions

Czaprowski Dariusz Night time is 8-10h/24. Part time is 12-16h/24h = out of school. Full time is 20h/24 or more

Donzelli Sabrina I think that part time is better than half time

Durmala Jacek Nighttime 6-10. Home-time= Half daytime 10-18. Full time= Total time 19-21. In practice (by me) maximun dosage is 20 hours

Hennes Axel it doesn´t make sense to have home-time and part-time

Kotwicki Tomasz None version approved.

Matthews Martin Add daytime up to 12 hours a day We should be aware that not all scoliosis treated with bracing is idiopathic in origin and therefore intervention wear prescription will be different to the standard concept of current treatment modalities.

Papadopoulos Dimitris Nighttime after exercises 10 hours per day - full time 20-21 hours per day

Parent Eric To version 4 I would add 13hr + to account for effect found in Braist2 and maybe change from 20 to 22-23 cut-off for full time.

Satyawati Rwahita : We evaluate hours of bracing simpler, every 6 hours. < 6 hrs; 6-11 hrs; 12-17 hrs; > 18 hrs based on minimum time of brace wearing should be > 18 hours. Usually our client choose to use rigid brace during day time at beginning of treatment

Tomasz Karski Point 80 is in my opinion (in every page of this questionnaire) in not important - but I had to be force to "write numbers")

Recommendation 18. At this stage of research on non-operative approaches during growth other than bracing, we strongly recommend to present radiographic results (C2)

## Results

| **Stage of Delphi Survey** | **Agreement** | | **Importance** | | |
| --- | --- | --- | --- | --- | --- |
|  | *Rating* | *%* | *Rating* | *Average* | *Median* |
| **3** | Good | 93% | High | 4.00 | 4 |
| **4** | Good | 92.17% | High | 3.91 | 4 |

## Results of the Consensus

| **Versions voted** | **Percentage of preferences** | **Preferences in the Boards** |
| --- | --- | --- |
| At this stage of research on non-operative approaches during growth other than bracing, we strongly recommend to present radiographic results | 42.4% | 41.2% |
| At this stage of research on non-operative approaches during growth other than bracing, we strongly recommend to present also radiographic results | 42.4% | 23.5% |
| At this stage of research on non-operative approaches during growth other than bracing, we strongly recommend to present radiographic results (mandatory) | 15.2% | 35.3% |

## Discussion 3

Bettany-Saltikov. I would strongly recommend a form of measurement like surface topography that has been shown to be reliable, at least for physical therapists. Doing this suggests we want to give patients more radiation. Beside many PTs in private practice do not have access to radiographs. Also x-rays are not 3D. This suggestion is very biased towards medics and against PTs I think. It is amazing that after over 100 years of using x-rays and despite all the new technology available that does not include putting x-rays into kids they are still considered to be the most important outcomes by many. Cochrane does not consider x-rays to be primary patient oriented outcomes either.

Stikeleather. We strongly recommend that all research results on non-operative patients_during growth be substantiated by radiographic evidence.

Zaina. At least one year of treatment during adolescence. Avoid mixed groups with juveniles, adolescents and young adults as frequently happen

Negrini. The problem of consistency of results in non-operative treatments has been considered in other recommendations. The concept could eventually be added also to this recommendation. Rephrasing for English still to be done.

## Discussion 4

There are perplexities for different reasons on this recommendation, since radiographies are not considered the best outcome for exercises, and also the radiation exposure is considered a limit.

Axel Hennes the focus is on patient related factors like HQL, cosmesis, coping strategies

Eric Parent This would limit research on some therapies where therapists would have access to radiograph results. I object to the mandatory. we should also refer to what is deemed adequate Radiographic follow-up.

Helma Voets X-rays every 8 months with a stable scoliosis

Helmut Diers Radiograhs are only 2-dimensional (except IOS) and never precise. IOS and ST deliver 3D information.

Josette Bettany-Saltikov to present radiographic results if possible there are instances where parents do not want their kids x-rayed or x-rayed many times

Karski Tomasz Important NEW prophylaxis

Patrick Knott As long as radiographs are medically indicated. Children should not be exposed to unnecessary radiation.

Piet Van Loon improvement posture, strength, range of motion and lifestyle are not measured on a X ray. Most of prehospital care should be measured by other means . For the pure morphologic part of scoliosis this can be the scoliometer or surface topography.

## Suggestions for new versions

Axel Hennes we should recommend on modern radiographics like 3D x-rax, MRI in standing, surface topography

Eric Parent We should delete mandatory. There should be a discussion on the risk of radiograph and adequate frequency.

Josette Bettany-Saltikov remove the word mandatory and replace with the word if possible

Karski Tomasz In discussion like in other points of paper

Tomasz Kotwicki This recommendation is not necessary

Title: Recommendations for research studies on Treatment of Idiopathic Scoliosis

The discussion focused on the definition “non-operative treatment”. There was quite big disagreement. The vote during Consensus will serve for future discussions.

## Results of the Consensus

| **Versions voted** | **Percentage of preferences** | **Preferences in the Boards** |
| --- | --- | --- |
| Recommendations for research studies on Treatment of Idiopathic Scoliosis | 40.6% | 50.0% |
| Recommendations for research studies on Idiopathic Scoliosis: Bracing, Specific Physiotherapeutic Scoliosis Exercises, or other fusion-less treatments. | 40.6% | 35.7% |
| Recommendations for research studies on Idiopathic Scoliosis: Bracing, Specific Physiotherapeutic Scoliosis Exercises, or other Functional fusion-less treatments. | 18.8% | 14.3% |

## Results at stage 6

| **Rank** | **Proposed definition** | **Total** | **Number of “Perfect”** | **Number of**  **“Totally not acceptable”** |
| --- | --- | --- | --- | --- |
| **1** | *Bracing and Scoliosis Physiotherapeutic Specific Exercises (SPSE)* | 36 | 15 | 0 |
| **2** | *Conservative* | 34 | 11 | 0 |
| **3** | *Non­operative* | 30 | 8 | 0 |
| **4** | *Bracing and Exercises* | 27 | 8 | 0 |
| **5** | *Non­surgical* | 18 | 7 | 1 |
| **6** | *Orthopedic and Rehabilitation Medicine* | 8 | 4 | 0 |
| **7** | *Physical and Rehabilitation Medicine* | 4 | 3 | 1 |
| **8** | *Orthopedic and Rehabilitation* | 3 | 3 | 0 |
| **9** | *Rehabilitation Medicine* | 2 | 4 | 1 |
| **10** | *Rehabilitation* | -6 | 2 | 2 |
| **11** | *Functional* | -7 | 4 | 2 |
| **12** | *Medical* | -15 | 3 | 6 |

## Results at stage 4

| **Rank** | **Proposed definition** | **Average** | **SD** | **Median** | **Preference 1-5** |
| --- | --- | --- | --- | --- | --- |
| **1** | *Bracing and Exercises* | 2.62 | 1.35 | 2 | 114 |
| **2** | *Bracing and Scoliosis Physiotherapeutic Specific Exercises (SPSE* | 2.42 | 1.39 | 2 | 121 |
| **3** | *Orthopedic and Rehabilitation Medicine* | 2.75 | 1.27 | 3 | 36 |
| **4** | *Rehabilitation Medicine* | 2.92 | 1.54 | 2 | 14 |
| **5** | *Non­operative* | 2.93 | 1.58 | 3 | 77 |
| **6** | *Conservative* | 3.08 | 1.22 | 3 | 105 |
| **7** | *Non­surgical* | 3.14 | 1.52 | 3 | 66 |
| **8** | *Physical and Rehabilitation Medicine* | 3.21 | 1.28 | 3 | 38 |
| **9** | *Rehabilitation* | 3.23 | 1.52 | 4 | 17 |
| **10** | *Orthopedic and Rehabilitation* | 3.49 | 1.29 | 4 | 45 |
| **11** | *Functional* | 3.71 | 1.02 | 4 | 63 |
| **12** | *Medical* | 4.33 | 1.13 | 5 | 24 |

## Question at stage 6 (Consensus)

The issue of giving the name to what we do is really hard. At this point there are at least 6-7 good choices, but no one prevails, nor can definitively be eliminated.

Presumably the issue will not be solved in this Consensus, and perhaps we will apply the title without any real specification (i.e.: Recommendations for research studies on treatment of Idiopathic Scoliosis). Anyway, we think it wise to continue the discussion at the end of the Consensus Session, so to deepen the topic and prepare future possible steps forward also on this issue.

Before voting again, please look at the following table, and carefully consider pros and cons in a wide perspective: what we need is a definition not excluding other people and/or future possible treatments, but possibly inclusive for other specialists and professionals, as well as future therapies.

(+ Advantage; - Limit; ±Intermediate; NA Not applicable)

|  | **Appropriateness** | **Future generalizability** | **Specialty inclusive** | **SRS use** | **SOSORT use** | **Definition by inclusion** | **Tradition** | **Other** |
| --- | --- | --- | --- | --- | --- | --- | --- | --- |
|  | what we do today in this field | it will include future developments | Independency from a specific specialty |  |  | What we do, not what we don’t do |  |  |
| **Bracing and Exercises** | + | - | - | - | + | + | ± |  |
| **Bracing and Physiotherapeutic Scoliosis Specific Exercises (PSSE)** | + | - | - | - | + | + | - |  |
| **Orthopedic and Rehabilitation Medicine** | + | + | - | ± | + | + | + |  |
| **Rehabilitation Medicine** | ± | ± | - | - | ± | + | - |  |
| **Non­operative** | + | ± | - | + | - | - | - |  |
| **Conservative** | + | ± | + | ± | ± | - | + | Today there is also “conservative surgery” |
| **Non­surgical** | + | ± | - | + | - | - | + |  |
| **Physical and Rehabilitation Medicine** | ± | ± | - | - | - | + | - |  |
| **Rehabilitation** | ± | ± | - | - | - | + | - |  |
| **Orthopedic and Rehabilitation** | + | ± | - | - | + | + | + |  |
| **Functional** | ± | ± | + | - | - | ± | - | Related to ICF classification Not well known |
| **Medical** | + | + | + | - | - | + | - | The opposite of surgical |

Vote have been given in this way:

1. Totally acceptable 2 points
2. Acceptable 1 point
3. Indifferent 0 points
4. Unacceptable -1 point
5. Totally unacceptable -2 points

## Question at stage 4

Rank the following terms according to your preference (to be used in the title and througout the document) Only the first 5 ranked answers will be retained for further analysis 1. Best 5. Worst From 6 to 12. Eliminated

## Discussion 1-2

**Hresko.** As mentioned elsewhere, clear definitions of terms is mandatory. “non-operative” is a very explicit term.. “Conservative” is a value judgment , subjective term. Many people, including my mentor Dr John Hall, would say “conservative to what”. Bracing is very radical , not conservative, to some of my patients. I think the term conservative is not precise. 2^nd^ point: Miniamlly invasive surgery is coming. Some would call MIS conservative relative to spinal fusion—I may be one of those persons. Very soon, MIS surgery will be compared to bracing and other non operative treatments. The same inclusion criteria we develop should be used by the MIS tethering proponents . So , I would propose that we consider terms such as “non- fusion”, “motion preservation” “ Functional treatment” though out the process. We then strictly define the terms and the rational for the terms.

**Negrini**. My preferred term, according to the name of our Society, would be “orthopedic and rehabilitation treatment”, since either “conservative” and “non-operative” define by exclusion. To me, a definition should define what is done, and not “everything apart another thing” (I mean, everything apart surgery). The old term “conservative” include all what is today in the field of orthopedic medicine (not-surgical) but also “rehabilitation medicine”. That’s the reason for my proposal.

**O’Brien**. I do envision a day when a minimally invasive apical surgical procedure may be deemed to be the optimal treatment for spinal deformity and even “conservative” depending on the defined judgement criteria. In this regard, “Non Operative” is a clearer and generally a more widely accepted term to describe the forms of treatment we are addressing in this current Consensus. Contrary to your point however I believe this term is too inclusionary rather than exclusionary and further definition and classifications may be helpful. To SOSORT members “Non Operative” primarily refers to various Bracing and Scoliosis Specific Exercises (schools), while outside reference may be towards a far broader range with different meaning – ie, Spine (Phila Pa 1976). 2010 Mar 1;35(5):578-82. doi: 10.1097/BRS.0b013e3181b0f2f8. The costs and benefits of nonoperative management for adult scoliosis. Glassman SD1, Carreon LY, Shaffrey CI, Polly DW, Ondra SL, Berven SH, Bridwell KH. Duration of use and frequency of visits were collected for 8 specific treatment methods: medication, physical therapy, exercise, injections/blocks, chiropractic care, pain management, bracing, and bed rest. Other studies refer to Acupuncture, Yoga, Tai Chi, and a host of other medical, alternative, or complentary active or passive treatments. I think this is confusing and we may be better served in the future by developing sub class categories to differentiate these forms of treatment rather than rely (and be judged) on one broad term.

**Negrini**. After reflection, starting from the main idea that defining by exclusion is not correct, and that also in our basic medical studies we use the categories “surgical” and “medical”, I propose “medical approach” instead of “non-operative” or “conservative”

**Knott**. I would INCLUDE rehabilitation exercises and bracing for now, and then ADD more treatments to this list as we wish to study them. This way we exclude other treatments being lumped in with them (eg. chiropractic, acupuncture, etc.)

**Kotwicki**. non-surgical treatment

**Stikeleather**. SRS SOSORT initiative, we are attempting to establish a well defined inclusion criteria that will be universally accepted.” Non –operative” leaves little room for confusion.

**Zaina**. Rehabilitation treatment for scoliosis

## Discussion 3

**Akin Ugras.** Non-operative treatment is a clearer term for surgeons

**Bettany-Saltikov.** Recommendations for research studies on bracing & physiotherapeutic scoliosis-specific exercise treatment (or rehabilitation exercises) of Idiopathic Scoliosis. I would suggest either Bracing and Physiotherapeutic Scoliosis Specific exercises PSSE (or rehabilitation exercises) or non-surgical interventions. “non- fusion”, “motion preservation” or “ Functional treatment” could also refer to some innovative surgical techniques that are currently being developed.

**De Mauroy.** Recommendations for research studies on bracing & exercises treatment of Idiopathic Scoliosis. I recognize that the term "conservative" is not appropriate because it means “designed to preserve parts or restore or preserve function” and can be applied as well to surgery than non-operative. Medical is not pertinent as surgery is a branch of medicine requiring operative procedure. The ideal would be to combine "bracing & exercises" why not: [ Bracing & Exercises treatment ]

**Durmala**. Bracing and exercises

**Kotwicki.** Recommendations for research studies on bracing & exercises treatment of Idiopathic Scoliosis

**Negrini**. I continue to stress the importance of a positive and comprehensive definition of what we do. Moreover, it must be comprehensive and open to new future possibilities when eventual new treatment will show their effectiveness. For sure what we do is all what is not surgery. At the University during medicine, we study medical pathology and surgical pathology, then a couple of years later medical therapy and surgical therapy, at least in Italy. If the same is true in the rest of the world I continue to think that “medical treatment is the most appropriate terms since it remains open to new future options for treatment, is not linked to a specific specialty, and define what is not surgical by inclusion and not exclusion.

**Rigo**. Non-Surgical is OK with me

**Romano**. Conservative treatment

## Discussion 5

**Hresko Timothy.** A suggestion on a possible alternative title: Recommendations for research studies on treatment of Idiopathic Scoliosis.

**Stefano Negrini.** On one side I think that raising the issue and discussion is important, since we cannot continue with a name that we do not feel as representative for all people involved (specifically inside our Society, that is exclusively devoted to this). I did not perform the analysis according to specialty and/or profession and/or Society, but I think there will be clear differences on the choices.

I suggest to discuss (and vote) in any case the issue of the name, but leaving your wise proposal as the possible last decision if we will no reach anough agreement and we will decide to continue the discussion in the next future.

**Tomasz Kotwicki.** My opinion about the title: evidently it will be extremely difficult to

reach consensus. So, we'd better stay simple: non-operative treatment. Since the word "conservative" (which is good and neutral for me) can be felt by our English native speaking collegues as emotionally marked, we'd better avoid it. For me the "fusion-less" , "functional" etc. are also very attractive. In the near future the paper would be used also by surgeons reporting results of minimal fusion or fusion-less procedures. In fact, the recommendations stay valid for both non-operative and surgical fusion-less procedures. This can be at least mentioned in the final paper.

**Patrick Knott.** My Ranking: 1. Conservative 2. Non-Operative 3. Non-Surgical 4. Bracing and Exercises 5. Bracing and Physiotherapeutic Scoliosis Specific Exercises

**Theodoros B Grivas.** Non-operative or Medical

**Steve Glassman.** I also agree with Non-operative as the best choice.

**Joe O’Brien.** The selection of a term, on the surface, seems so simple, yet the underlying issues are varied, somewhat territorial and complicated. In reality, every term listed has their purpose and rationale as well as their shortfalls and limitations, depending on the focus. I find your last column in the first table showing the number of people who ranked the term within the 1-5 range to be the most telling. It would be interesting to see this data further broken out by profession. To me, Non Operative and Non Surgical are synonymous terms and clearly define a top level form of treatment easily categorized and understood by all.....it is either surgery or not. For this reason it is my overall choice. However I do have two concerns, which may be more political than scientific; 1. The term is too broad and lumps together everything from raindrop therapy, to chiropractic, to electric stimulation, and beyond which creates further confusion and challenges to develop an accepted standard of care. Several studies are critical of the cost or effectiveness of "Non Operative" treatment that seemed to include everything except bracing and scoliosis specific exercises. Thus, I understand the desire to use the more specific term, "Bracing & SSE". 2. The term tends to reinforce a we versus us mentality rather than reflect a collaborative focus on optimal patient care. Similarly, I like the original term Conservative, not for the purposes at hand, but rather in reference to its full meaning as embodied in SOSORT's statutes: to foster the best conservative management.......early detection, prevention, patient care, education, information, and multidisciplinary team work. "Beauty is in the eye of the beholder" so it seems to me that it is critical in this consensus to constantly remind everyone what the goal is and try to keep them all focused with the same view.

**Hresko Timothy.** A title that is broad based yet gives specific emphasis on the 2 main issues for consideration might attractive. We can combine several of the top choices into one title, my suggestions. 1. Title: Recommendations for research studies on Idiopathic Scoliosis: Bracing, Exercise , or other Functional fusion-less treatments. 2. Title: Recommendations for research studies on Idiopathic Scoliosis: Bracing, Scoliosis Specifc Exercise , or other fusion-less treatments. Or simply 3. Title: Recommendations for research studies on Treatment of Idiopathic Scoliosis

**Nigle Price.** As a doctor who has cared for scoliosis for 20 years I can tell you that surgery is often the conservative thing to do for some patients when all else fails, so it should probably be changed to something different. It does mean a lot of different things to different people.

**Josette Bettany-Saltikov.** I agree with the two statements or recommendations you suggested and I suggest the terms1. non-surgical or 2. non-operative (more inclusive) 3.braces and SSE

# Last comments

Suggestions for focusing more and better on exercises, and the need for more balanced outcome reports.

Bettany-Saltikov Josette Some of the questions at the end are mainly for bracing. Could we add the equivalent quesions for PSSE?

Betz Joseph You guys are awesome!!!!!!

Brox Jens Ivar Too many questions

Hennes Axel There should be more efforts taken in the improvement of exercise based approaches rather than focus on competitive interests between the scoliosis schools. Therefore we need more PT´s trained in scoliosis specific exercises and inspire them to document their treatment results.

Kerstholt Janine I agree with Bettany-Saltikov in presenting results from studies on PSSE like in discussion point 16

Lebel Andrea define the "Wait and See" approach criteria in scoliosis care, which it is still widely used as a non-surgical method. Set Risser and Cobb baseline when "Wait and See" would be appropriate: example: Risser 0 - Cobb 10, Risser 1- Cobb 10-15 Risser 2 - Cobb 15-20, Risser 3 - Cobb 20-25 degrees (30+ any Risser should be benefitting of Non-surgical method in preventing future pain and progression)

Marti Cindy I would also consider if doing exercise intervention as part of research, that it be reported the years of experience of the PT (delineating x hrs per week avg of scoliosis care to be considered a qualifying year of experience or x patients treated prior to being in the study). Also, when reporting compliance for exercise, report quality of exercise execution as graded regularly by the PT and compliance not only with home program but with PT. I also really strongly recommend a balanced outcome reporting of aesthetic, respiratory, and radiographic in outcomes.

Monroe Marcia I am looking forward to the meeting and learning further how to best understand and treat the pathology.

Muccio Marissa Thank YOU!!!!!!

Parent Eric There should be recommendation on reporting at minimum some therapy description elements: EG frequency of therapy visits, duration, EG home program parameters, Duration, number of exercises EG. recommendation of dosages... EG. progression or guidelines to select intensity of therapy EG. certification of the therapists We should recommend publishing protocols, registering trials We should recommend reporting following CONSORT recommendations... We should emphasize need to blind evaluators We should emphasize the need to report intent-to-treat analysis and quantify drop outs. We should request reasons for drop outs. We should request subgroup data reporting based on curve types (anatomical location if not school specific.) I think recommendations should be separate for bracing and exercises and then any other therapies. Therefore The choice of non-operative/ conservative... should be moot. we should name the therapies where this applies.

Sanchez Judith Thanks you for your great work

Tomasz Karski Yes - aetiology of the so called idiopathic scoliosis in biomechanical (publications 1995 - 2007 / 2014). Deformity of spine and trunk start to develop in age of 2 - 3 years in every type in new classification. When the child start to "stand - on the right leg" and start to walk. The children on world need CAUSAL PROPHYLAXIS. Look www.ortopedia.karski.lublin.pl

Van Loon Piet Lifestyle and culture of the youth will not change by the cause of high level evidence of some , more late stage treatment modalities. Prevention has to be the goal and broad awareness by parents and school employees is needed.

Wong M. S. None. Thanks for your great coordination!

# References

1. Negrini S, Aulisa AG, Aulisa L, Circo AB, de Mauroy JC, Durmala J, Grivas TB, Knott P, Kotwicki T, Maruyama T *et al*: **2011 SOSORT guidelines: Orthopaedic and Rehabilitation treatment of idiopathic scoliosis during growth**. *Scoliosis* 2012, **7**(1):3.

2. Hresko MT: **Clinical practice. Idiopathic scoliosis in adolescents**. *N Engl J Med* 2013, **368**(9):834-841.

3. Negrini S, Grivas TB, Kotwicki T, Maruyama T, Rigo M, Weiss HR: **Why do we treat adolescent idiopathic scoliosis? What we want to obtain and to avoid for our patients. SOSORT 2005 Consensus paper**. *Scoliosis* 2006, **1**:4.

4. Mayo NE, Goldberg MS, Poitras B, Scott S, Hanley J: **The Ste-Justine Adolescent Idiopathic Scoliosis Cohort Study. Part III: Back pain**. *Spine* 1994, **19**(14):1573-1581.

5. Weinstein SL, Dolan LA, Spratt KF, Peterson KK, Spoonamore MJ, Ponseti IV: **Health and function of patients with untreated idiopathic scoliosis: a 50-year natural history study**. *Jama* 2003, **289**(5):559-567.

6. Weinstein SL, Ponseti IV: **Curve progression in idiopathic scoliosis**. *J Bone Joint Surg Am* 1983, **65**(4):447-455.

7. Weinstein SL, Dolan LA, Wright JG, Dobbs MB: **Effects of bracing in adolescents with idiopathic scoliosis**. *N Engl J Med* 2013, **369**(16):1512-1521.

8. Weinstein SL, Dolan LA, Wright JG, Dobbs MB: **Design of the Bracing in Adolescent Idiopathic Scoliosis Trial (BrAIST)**. *Spine (Phila Pa 1976)* 2013, **38**(21):1832-1841.

9. Weinstein SL, Dolan LA, Cheng JC, Danielsson A, Morcuende JA: **Adolescent idiopathic scoliosis**. *Lancet* 2008, **371**(9623):1527-1537.

10. Stagnara P: **Les deformations du rachis**. Paris: Masson; 1985.

11. Kotwicki T: **Improved accuracy in Risser sign grading with lateral spinal radiography**. *Eur Spine J* 2008, **17**(12):1676-1685.

12. Nault ML, Parent S, Phan P, Roy-Beaudry M, Labelle H, Rivard M: **A modified Risser grading system predicts the curve acceleration phase of female adolescent idiopathic scoliosis**. *J Bone Joint Surg Am* 2010, **92**(5):1073-1081.

13. Richards BS, Bernstein RM, D'Amato CR, Thompson GH: **Standardization of criteria for adolescent idiopathic scoliosis brace studies: SRS Committee on Bracing and Nonoperative Management**. *Spine* 2005, **30**(18):2068-2075; discussion 2076-2067.

14. Negrini S, Grivas TB, Kotwicki T, Rigo M, Zaina F: **Guidelines on "Standards of management of idiopathic scoliosis with corrective braces in everyday clinics and in clinical research": SOSORT Consensus 2008**. *Scoliosis* 2009, **4**(1):2.
